# Variation in the Intensity of Selection on Codon Bias over Time Causes Contrasting Patterns of Base Composition Evolution in *Drosophila*

**DOI:** 10.1093/gbe/evw291

**Published:** 2017-01-12

**Authors:** Benjamin C. Jackson, José L. Campos, Penelope R. Haddrill, Brian Charlesworth, Kai Zeng

**Affiliations:** 1 Department of Animal and Plant Sciences, University of Sheffield, Sheffield, United Kingdom; 2 Institute of Evolutionary Biology, School of Biological Sciences, University of Edinburgh, Edinburgh, United Kingdom; 3 Centre for Forensic Science, Department of Pure and Applied Chemistry, University of Strathclyde, Glasgow, United Kingdom

**Keywords:** codon usage bias, nonequilibrium behavior, selection, short introns, *Drosophila*

## Abstract

Four-fold degenerate coding sites form a major component of the genome, and are often used to make inferences about selection and demography, so that understanding their evolution is important. Despite previous efforts, many questions regarding the causes of base composition changes at these sites in *Drosophila* remain unanswered. To shed further light on this issue, we obtained a new whole-genome polymorphism data set from *D. simulans*. We analyzed samples from the putatively ancestral range of *D. simulans*, as well as an existing polymorphism data set from an African population of *D. melanogaster*. By using *D. yakuba* as an outgroup, we found clear evidence for selection on 4-fold sites along both lineages over a substantial period, with the intensity of selection increasing with GC content. Based on an explicit model of base composition evolution, we suggest that the observed AT-biased substitution pattern in both lineages is probably due to an ancestral reduction in selection intensity, and is unlikely to be the result of an increase in mutational bias towards AT alone. By using two polymorphism-based methods for estimating selection coefficients over different timescales, we show that the selection intensity on codon usage has been rather stable in *D. simulans* in the recent past, but the long-term estimates in *D. melanogaster* are much higher than the short-term ones, indicating a continuing decline in selection intensity, to such an extent that the short-term estimates suggest that selection is only active in the most GC-rich parts of the genome. Finally, we provide evidence for complex evolutionary patterns in the putatively neutral short introns, which cannot be explained by the standard GC-biased gene conversion model. These results reveal a dynamic picture of base composition evolution.

## Introduction

Here, we investigate the forces that affect evolution at 4-fold degenerate coding sites in *Drosophila simulans* and *D*. *melanogaster*. These sites represent a substantial part of the genome and are often used as references against which selection at other sites, for example, nonsynonymous sites, is tested (McDonald and Kreitman 1991; Rand and Kann 1996; Parsch et al. 2010; Stoletzki and Eyre-Walker 2011). Quantifying the forces that affect their evolution is necessary both for a general understanding of genome evolution and for making robust inferences about the influences of demographic factors and selection elsewhere in the genome (Matsumoto et al. 2016).

Codon usage bias (CUB) is a key feature of 4-fold sites, since it involves the disproportionate use of certain codons among the set of codons that code for a given amino acid. There is evidence for CUB in a wide range of organisms, including both prokaryotes and eukaryotes (Drummond and Wilke 2008; Hershberg and Petrov 2008). The most common explanation for CUB is that this maximizes translational efficiency and/or accuracy (Hershberg and Petrov 2008). Avoidance of the toxicity of misfolded proteins generated by translational errors has also been proposed as an explanation of CUB (Drummond and Wilke 2008). Recent work has also suggested the possibility that stabilizing, as opposed to directional, selection maintains the frequencies of synonymous codons, because CUB has been found to be unrelated to recombination rate in *D. pseudoobscura*, in line with theoretical predictions about the action of stabilizing selection (Charlesworth 2013; Fuller et al. 2014; Kliman 2014).

In most species of *Drosophila* for which data are available, including *D. melanogaster* and *D. simulans*, all the preferred codons are GC-ending (Vicario et al. 2007; Zeng 2010). Selection for preferred codons thus acts to increase the GC content of third position sites in coding sequences (CDSs), and GC-ending and AT-ending codons have been often used as proxies for preferred and unpreferred codons, respectively. As in other species, evidence for selection for preferred codons in *D. melanogaster* comes from the fact that the level of codon bias is related to expression level (e.g., Duret and Mouchiroud 1999; Hey and Kliman 2002; Campos et al. 2013). There is also a negative relationship between the level of CUB and synonymous site divergence in the *Drosophila melanogaster* subgroup, consistent with selection for preferred codons (Shields et al. 1988; Powell and Moriyama 1997; Dunn et al. 2001; Bierne and Eyre-Walker 2006).

However, analyses based on between-species sequence divergence have consistently revealed an excess of substitutions towards AT-ending codons in the *D. melanogaster* lineage (Akashi 1995, 1996; McVean and Vieira 2001; Poh et al. 2012). Two hypotheses have been proposed for this observation. These are, firstly, that *D. melanogaster* has undergone a reduction in the population-scaled strength of selection for preferred codons, $4N_{e}s$, where $N_{e}$ is the effective population size and s is the selection coefficient favoring preferred codons in heterozygotes for the preferred allele. This reduction in selection could be caused either by a reduction in $N_{e}$ (Akashi 1996), or a reduction in s, perhaps due to changed ecological conditions (Clemente and Vogl 2012a, 2012b). The second explanation is that *D. melanogaster* has undergone a shift in mutational bias towards AT alleles (Takano-Shimizu 2001; Kern and Begun 2005; Zeng and Charlesworth 2010a; Clemente and Vogl 2012b). It has also been argued that both factors must be invoked to explain patterns of variation and evolution in the *D. melanogaster* lineage (Nielsen et al. 2007; Clemente and Vogl 2012a, 2012b).

Several attempts to detect selection on codon bias in *D. melanogaster* have come to conflicting conclusions. For instance, some polymorphism-based studies managed to detect evidence for selection favoring GC-ending codons (Zeng and Charlesworth 2009; Campos et al. 2013), although the intensity of selection may be weak relative to other *Drosophila* species (Kliman 1999; Andolfatto et al. 2011). However, other studies did not find support for such ongoing selection (Clemente and Vogl 2012a; Vogl and Clemente 2012; Poh et al. 2012). Thus, there is a pressing need to gain a better understanding of the dynamics of selection on codon bias and understand the sources of these conflicting results.

Much less is known about *D. simulans*. Early studies based on a small number of loci suggest that this species may be at base composition equilibrium, with the number of substitutions from AT-ending codons to GC-ending codons not statistically different from that in the opposite direction (e.g., Akashi 1995, 1996; Kern and Begun 2005; Akashi et al. 2006; Haddrill and Charlesworth 2008). However, more recent analyses have revealed AT-biased substitution patterns (Begun et al. 2007; Poh et al. 2012), suggesting a possible reduction in selection intensity in this lineage, although the reduction may be less severe compared with that in *D. melanogaster* (McVean and Vieira 2001). In contrast to the situation in *D. melanogaster*, the few polymorphism-based studies in *D. simulans* generally point to evidence for selection for preferred codons (Akashi 1997, 1999; Kliman 1999; Andolfatto et al. 2011). It is therefore unclear whether/how selection intensity has changed over time in *D. simulans*, and how the dynamics of base composition evolution differ from those in *D. melanogaster*.

Irrespective of the reason(s) for the AT-biased substitution pattern in these two *Drosophila* lineages, these findings present a problem for ancestral state reconstruction, a process that is necessary for inferring substitution patterns along a lineage of interest and for polarising segregating sites into ancestral and derived variants to understand their more recent evolution. Use of maximum parsimony methods or maximum likelihood models that assume equilibrium base composition under such circumstances can lead to erroneous inferences although these two methods were used in many previous analyses of various *Drosophila* species (Akashi et al. 2007; Matsumoto et al. 2015). Departures from base composition equilibrium may also lead to complex polymorphism patterns (Zeng and Charlesworth 2009). Both of these sources of difficulties may contribute to the mixed evidence for the nature of the forces acting on synonymous sites in *Drosophila* (Zeng and Charlesworth 2010a; Clemente and Vogl 2012a).

A factor that may confound the study of CUB is GC-biased gene conversion (gBGC), which is a recombination-associated process, and acts to increase GC content at sites where recombination occurs (Duret and Galtier 2009). Most studies have found little or no evidence for gBGC in *D. melanogaster* (Clemente and Vogl 2012b; Comeron et al. 2012; Campos et al. 2013; Robinson et al. 2014), although there is some evidence either for the action of selection for GC basepairs or gBGC on the evolution of non-coding sequences in *D. simulans* (Haddrill and Charlesworth 2008). In order to control for gBGC, we have analyzed data on the 8–30-bp region of short introns (SIs), which are widely considered to be evolving near-neutrally in *Drosophila* (Halligan and Keightley 2006; Parsch et al. 2010; Clemente and Vogl 2012b).

To address the questions raised above, we need to look at both divergence and polymorphism data from both species; the analyses should explicitly take into account departures from equilibrium, so that signals of selection can be detected without biases. To this end, we have obtained new whole-genome data from *D. simulans* and used an existing high-quality data set for *D. melanogaster*. Using the reference genome of *D. yakuba* as an outgroup, we used state-of-the-art methods to reconstruct ancestral states. In addition, we employed methods that can infer selection intensity on different timescales, along the *D. melanogaster* and *D. simulans* lineages, with the aim of shedding further light on the evolutionary dynamics of genome composition in these two species.

## Materials and Methods

### Sequence Data Preparation

We first describe the sequencing of 22 new *D. simulans* isofemale lines, 11 of which were collected by William Ballard in 2002 from Madagascar (MD lines: MD03, MD146, MD197, MD201, MD224, MD225, MD235, MD238, MD243, MD255, and MD72); the other 11 were collected by Peter Andolfatto in 2006 from Kenya (NS lines: NS11, NS111, NS116, NS19, NS37, NS49, NS63, NS64, NS89, NS95, and NS96). We produced homozygous lines by full-sib inbreeding in the Charlesworth lab for nine generations; however, six lines (NS11, NS63, NS116, MD224, MD243, and MD255) were lost early in the process of inbreeding. For these lines, we sequenced the initial stocks that we had received from the Andolfatto lab. Genomic DNA was prepared for each isofemale line by pooling 25 females, snap freezing them in liquid nitrogen, extracting DNA using a standard phenol-chloroform extraction protocol with ethanol, and ammonium acetate precipitation. These flies were sequenced by the Beijing Genomics Institute (BGI; http://bgi-international.com/; last accessed December 28, 2016). A 500-bp short-insert library was constructed for each sample, and the final data provided consisted of 90-bp paired-end Illumina sequencing (pipeline version 1.5), with an average coverage of 64×. We double-checked the quality of the filtered reads for each allele with FastQC (available at http://www.bioinformatics.babraham.ac.uk/projects/fastqc/; last accessed December 28, 2016), and no further trimming was necessary. The raw reads have been deposited in the European Nucleotide Archive, study accession number: PRJEB7673. 

We obtained sequence data for 20 further *D. simulans* isofemale lines from Rogers et al. (2014). These lines were from the same sampling localities in Kenya (10 lines: NS05, NS113, NS137, NS33, NS39, NS40, NS50, NS67, NS78, and NS79) and Madagascar (10 lines: MD06, MD105, MD106, MD15, MD199, MD221, MD233, MD251, MD63, and MD73) as above. Each line was sequenced on between 2 and 3 lanes of paired-end Illumina sequencing at the UCI Genomics High-Throughput Facility (http://ghtf.biochem.uci.edu/; last accessed December 28, 2016) per line. Further information about these lines and their sequencing is available in the study by Rogers et al. (2014). After examining FastQC files for these 20 lines, we trimmed two lines with apparently lower quality scores (MD233 and MD15) using the trim-fastq.pl script from Popoolation 1.2.2 (Kofler et al. 2011) with the (minimum average per base quality score) quality-threshold flag set to 20.

Downstream of sequencing, we combined both data sets and used a BWA/SAMtools/GATK pipeline, previously described in Campos et al. (2014) and Jackson et al. (2015), to generate genotype calls. Briefly, we aligned and mapped reads for each *D. simulans* line to the second-generation assembly of the *D. simulans* reference sequence (Hu et al. 2013) using BWA 0.7.10 (Li and Durbin 2009). We used SAMtools 1.1 (Li et al. 2009) to filter alignments with a mapping quality <20, and to sort and index the resulting alignments. To combine reads from one sample across multiple lanes, we used Picard tools 1.119(http://broadinstitute.github.io/picard/; last accessed December 28, 2016) to edit BAM file headers and SAMtools 1.1 to merge, resort and index BAM files per sample. We then used Picard tools 1.119 to fix mate information, sort the resulting BAM files and mark duplicates. We performed local realignment using the RealignerTargetCreator and IndelRealigner tools of GATK 3.3 (https://www.broadinstitute.org/gatk/; last accessed December 28, 2016).

For single nucleotide polymorphism (SNP) calling, we used the UnifiedGenotyper for diploid genomes (parameter: sample_ploidy 2) and generated a multisample VCF file (Danecek et al. 2011). Subsequently, we performed variant quality score recalibration (VQSR) to separate true variation from machine artefacts (DePristo et al. 2011). We used biallelic and homozygous (for a given individual) SNPs detected at 4-fold sites at a frequency equal to or higher than seven sequenced individuals as the training set. Six SNP call annotations were considered by the VQSR model: QD, HaplotypeScore, MQRankSum, ReadPosRankSum, FS, and MQ, as suggested by GATK (see http://www.broadinstitute.org/gatk/; last accessed December 28, 2016; DePristo et al. 2011). The SNPs were allocated to tranches according to the recalibrated score, so that a given proportion of the true sites were recovered. We retained variants that passed a cutoff of 95%, the variant score limit that recovers 95% of the variants in the true data set. We refer to this data set as “filtered.” From the multisample recalibrated VCF file, we made a consensus sequence FASTA file for each individual using a custom Perl script. The variant calls that did not pass the filter were called N (missing data) at the sites in question. We also generated an unfiltered data set, where we did not implement any form of variant score recalibration. We refer to this data set as “unfiltered.” The VCF files and the scripts used to produce them can be downloaded by following the hyperlink provided in http://zeng-lab.group.shef.ac.uk; last accessed December 28, 2016.

### Annotation of the *D. s* *imulans* Data Set

Using annotations from the *D. simulans* reference (Hu et al. 2013), we extracted CDSs for each gene and made FASTA alignments. We included the *D. simulans* reference sequence and the 1:1 FlyBase orthologous genes of *D. melanogaster* (release version 5.33) and *D. yakuba* (release version 1.3). We then performed amino acid sequence alignments using MAFFT (Katoh et al. 2002). These amino acid sequence alignments were translated back to nucleotides using custom scripts in PERL to produce in-frame CDS alignments that included the 42 *D. simulans* alleles and the *D. melanogaster* and the *D. yakuba* outgroups. We extracted 4-fold (and 0-fold) degenerate sites from CDS alignments which were 4-fold (0-fold) degenerate in all lines, with the condition that there was at most one segregating site in the codon to which the 4-fold (0-fold) site belonged. We retained the 4-fold (0-fold) sites from an alignment only if there were at least ten 4-fold (0-fold) sites in that alignment in total. For the polymorphism and substitution analyses on 4-fold sites reported in the Results, we carried out the same procedure with the added condition that sites must also be 4-fold degenerate in the three reference sequences.

We also extracted the intron coordinates from the *D. simulans* reference genome sequence. Genomes were masked for any possible exons. For each *D. simulans* intron, we obtained the corresponding orthologous intron of *D. melanogaster* (Hu et al. 2013). For *D. yakuba*, for each orthologous gene, we obtained all its annotated introns and blasted them against the *D. melanogaster* introns (of the same ortholog) with an e-value of <10^−^^5^ and selected the reciprocal best hit (because introns are generally short, the threshold e-value was conservative; see Results). We used RepeatMasker (http://www.repeatmasker.org) to mask repetitive elements in our intron data set, using the library of repeats for *D. melanogaster* and the default settings. We produced a final alignment of each intronic polymorphism data set of *D. simulans* with the corresponding *D. melanogaster* and *D. yakuba* orthologs using MAFFT.

We extracted positions 8–30 bp of all introns <66-bp long, based on the *D. melanogaster* reference alignment for each intron, as we considered the *D. melanogaster* reference to be the best annotated of the three species. To do this, we scanned the *D. melanogaster* reference sequence for each intronic alignment. We retained the alignment if the *D. melanogaster* reference sequence was <66-bp long (not including alignment gaps), and then further obtained the coordinates of the 8-bp position and the 30-bp position in the *D. melanogaster* reference sequence after discarding any gaps introduced by the alignment program. We then cut the whole alignment at these coordinates. These SI sites are thought to be close to neutrally evolving in *Drosophila*, based on their patterns of polymorphism and substitution (Halligan and Keightley 2006; Parsch et al. 2010; Clemente and Vogl 2012b).

### The *D. m* *elanogaster* Data Set

Similar analyses were performed using a *D. melanogaster* polymorphism data set, described in Jackson et al. (2015), which consists of 17 Rwandan *D. melanogaster* samples (RG18N, RG19, RG2, RG22, RG24, RG25, RG28, RG3, RG32N, RG33, RG34, RG36, RG38N, RG4N, RG5, RG7, and RG9) made available by the *Drosophila* Population Genomics Project 2 (Pool et al. 2012).

### Quality Control of *D. s* *imulans* Genotypes

The lines that were inbred successfully for nine generations to produce homozygous samples still retained low levels of residual heterozygosity, which may have been due to a failure to purge our lines of natural variation (Stone 2012), or to SNP calling errors (the latter should be less likely given the high coverage [64×] and our stringent SNP calling regime). We quantified the amount of residual heterozygosity per sample for each of the unfiltered and filtered data sets (supplementary fig. S1, Supplementary Material online). As expected, the filtered data set exhibited lower levels of residual heterozygosity (ND samples: mean value = 0.0616%, all values <0.5%; MD samples: mean value = 0.0168%, all values <0.15%). The six lines that were not subject to the inbreeding procedure (see above) did not have substantially higher levels of residual heterozygosity than the remaining samples, presumably because they were already considerably inbred after being kept as laboratory stocks for several years. For downstream analyses we treated heterozygous sites as follows: at each heterozygous site within a sample, one allele was chosen as the haploid genotype call at that site with a probability proportional to its coverage in the sample. The alternative allele was discarded. Because our samples are from partially inbred lines that originated from a mating between at least one wild male and only one wild female, heterozygosity at a site implies that the site is segregating in the wild population. By sampling one allele at random, we attempted to replicate the inbreeding process, which aimed to remove heterozygosity from within the lines.

Pairwise *π_S_* values (synonymous site diversity) for all 42 *D. simulans* lines showed three pairs of samples which deviated substantially from the distribution of pairwise *π_S_* between samples (mean *π_S_* for all samples = 0.030, SD = 0.0018). These pairs were MD201–NS116 (*π_S_* = 7.28 × 10^−^^5^); NS137–NS37 (*π_S_* = 0.0034) and NS49–NS96 (*π_S_* = 0.0097). A principal component analysis (PCA) of binary genotypes placed NS116 within the cluster of MD samples, and NS116 exhibited a more MD-like genetic distance to the *D. simulans* reference sequence. These results were based on the filtered data set, but the unfiltered data set returned qualitatively identical patterns (data not shown). We therefore excluded NS116 from all downstream analyses based on the likelihood of its representing labeling error. We also excluded NS37 and NS96 as these individuals had the highest levels of residual heterozygosity out of the remaining two pairs of closely related samples (supplementary fig. S1, Supplementary Material online).

To further assess the quality of our data sets, we compared polymorphism and divergence statistics to data previously published in the literature on *D. simulans* (see Results). In particular, we calculated a range of summary statistics per gene: *F_ST_*between NS and MD samples; *π*, Tajima’s *D*, *Δ_π_*, and *θ_W_* within the NS sample, within the MD sample, and for both samples combined. *Δ_π_* for a given gene (Langley et al. 2014) is defined as

(1) $$\Delta_{\pi }=\frac{\hat{k}}{S}-\frac{1}{\sum_{i=1}^{n-1}\left(1/i\right)}$$

where *k* represents the mean number of pairwise differences among the *n* alleles in the sample, and *S* is the number of segregating sites (Langley et al. 2014). We calculated this statistic using a modified version of the tajima.test() function from the pegas package (Paradis 2010) in R. *Δ_π_* is similar to Tajima’s *D* (Tajima 1989), but is normalized by the total amount of diversity. Its advantage over Tajima’s *D* is that it is less dependent on the total diversity for the sample (Langley et al. 2014). We also compared *K_A_* and *K_S_* between the three reference sequences (*D. melanogaster, D. simulans* and *D. yakuba*) in all CDS alignments using the kaks() function from the seqinr package in R, and *K_SI_*between the reference sequences in all our SI alignments using the dist.dna() function from the pegas package in R, based on the K80 method (Kimura 1980). These analyses are presented in the first section of the Results.

### Divergence-Based Analyses

We used three methods to determine the ancestral state at the *melanogaster-simulans* (*ms*) node, all of which used only the three reference sequences. First, we used parsimony, implemented in custom scripts in R. Second, we used the nonhomogeneous general time-reversible (GTR-NH_b_) substitution model, implemented in the baseml package of PAML v4.8 (Yang 2007), after checking that GTR-NH_b_ fitted the data better than the stationary GTR model using chi-squared tests (see Results). The use of this method to reconstruct ancestral sites when nucleotide composition is nonstationary is described in the study by Matsumoto et al. (2015) and has been shown to produce highly accurate results in the presence of nonequilibrium base composition, whereas the parsimony method is likely to be biased. Under the GTR-NH_b_ method, we implemented two ways of determining the ancestral state at the *ms* node, by either using the single best reconstruction (SBR) of the ancestral sequence at the *ms* node, or by weighting the four possible nucleotides at the *ms* node by the posterior probability of each. Instead of ignoring suboptimal reconstructions, as the parsimony and SBR methods do, the last option weights all the possible ancestral states by their respective posterior probabilities. Following Matsumoto et al. (2015), we refer to these two GTR-NH_b_-based methods as “SBR” and “AWP,” respectively. The AWP method should be more reliable than either parsimony or SBR when base composition is not at equilibrium (Matsumoto et al. 2015).

Since some of the models we used are very parameter-rich (e.g., the GTR-NH_b_ model has 39 parameters for three species, and the M1* model described more fully below has 25 parameters for *D. simulans* and 21 parameters for *D. melanogaster*, given the sample sizes), we had to group genes into bins to avoid overfitting. To investigate the relationship between selection and GC content at 4-fold sites (a proxy for the extent of CUB), we binned 4-fold sites by the GC content in the *D. melanogaster* reference sequence, which we used as a proxy for the historic strength of selection favoring GC alleles. GC content evolves very slowly over time (Marais et al. 2004), and is highly correlated between *D. simulans* and *D. melanogaster* CDS (Pearson’s correlation coefficient *r* = 0.97, *P* < 2.2 × 10^−^^16^), so this strategy should accurately represent GC content at the *ms* node. We binned 4-fold degenerate sites into 20 autosomal and four X-linked bins. Bins were chosen to maintain approximately the same number of genes per bin. The autosomal and X-linked SI sites were always treated as two separate bins. We also followed this binning convention for other analyses. When carrying out correlation analyses between GC content bins and other variables (e.g., substitution rate and estimates of the selection coefficient), we included only the 4-fold degenerate site GC bins, but not the SI bin. We also restricted the correlation analysis to the autosomal bins only. Given the small number of bins on the X chromosome, this type of analysis is underpowered; in fact, the smallest *P* value that Kendall’s *τ* can achieve with four data points is 0.08.

To determine whether or not *D. melanogaster* and *D. simulans* are in base composition equilibrium, for each bin we counted the numbers of S→W ($N_{S\to W}$), W→S ($N_{W\to S}$), and putatively neutral ($N_{neu}$) substitutions (i.e., S→S and W→W), where S represents G or C, the strong (potentially preferred) allele, and W represents A or T, the weak (potentially unpreferred) allele. We did this along each of the *D. melanogaster* and *D. simulans* lineages by (probabilistically) comparing the reconstructed ancestral states at the *ms* node with the reference genomes. This is reasonable because the branch length is much higher than the level of within-species polymorphism (see Results). For the AWP method, we rounded our results to the nearest integer. Where possible, we compared our results to those published in the literature, and to equivalent results kindly provided by Juraj Bergman and Claus Vogl (pers. comm.; supplementary table S2, Supplementary Material online). To obtain the W→S substitution rate ($r_{W\to S}$) per bin, we divided $N_{W\to S}$ by the total number of AT sites ($L_{W}$) at the *ms* node in that bin. Similarly, $r_{S\to W}=N_{S\to W}/L_{S}$.

### Polymorphism-Based Analyses

For each bin, we estimated the derived allele frequency (DAF) at segregating sites, using the three methods described above to infer ancestral states at the *ms* node, which should be a reasonable approximation given the rarity of shared polymorphism for the two species (Clemente and Vogl 2012b). We classified these sites into segregating sites at which the ancestral allele was AT and the derived allele was GC (${DAF}_{W\to S}$), and segregating sites at which the ancestral allele was GC and the derived allele was AT (${DAF}_{S\to W}$), as well as segregating sites which had mutated from A to T, or *vice versa*, and from G to C or *vice versa* (${DAF}_{neu}$). We also calculated *Δ_π_* (Langley et al. 2014) for each bin. We mostly display results obtained from the AWP method in the Results section, because it is probably the most reliable of the three. Qualitatively, the results are generally insensitive to the choice of method for reconstructing ancestral sites. Thus, we present a set of figures in the supplement (supplementary figs. S6–S11, Supplementary Material online) that are parallel to those shown in the main text, but were obtained using either parsimony or SBR, respectively.

We used two polymorphism-based methods for estimating the population-scaled strength of the force favoring GC alleles, $\gamma =4N_{e}s$, where $N_{e}$ is the effective population size and *s* is the selection coefficient against heterozygous carriers of the AT allele. The first is the method of Glémin et al. (2015), which uses three different classes of polarized unfolded site frequency spectra (SFS) for sites that are segregating in the present day: S→W, W→S, and putatively neutral (see above). This method is capable of taking into account polarization errors, which, if untreated, may lead to upwardly biases estimates of γ (Hernandez et al. 2007), by incorporating them into the model and estimating them jointly with the parameters of interest. It is also capable of correcting for demographic effects, by introducing nuisance parameters to correct for distortions in the SFS due to demography (after Eyre-Walker et al. 2006). Because it only considers the SFS of derived alleles, we expect this method to recover signatures of selection on a relatively recent time scale ($∼{4N}_{e}$ generations if we conservatively assume neutrality). We generated unfolded SFSs for this model using the AWP method to infer the ancestral state at the *ms* node and estimated the strength of γ using R code provided in the supplementary material of Glémin et al. (2015). We refer to the models using this method with the same notation as Glémin et al. (2015). These are model M0, where γ=0 and polarization errors are not taken into account; M1, where γ≠0 and polarization errors are not taken into account; and M0* and M1*, which are the equivalent models after correcting for polarization errors. Note that the method for controlling for demography drastically increases the number of model parameters. For instance, for M1, in addition to γ and the three mutational parameters for each of the three SFSs ($\theta =4N_{e}\mu$), it requires an additional *n* – 2 nuisance parameters, where *n* is the number of frequency classes (in our case, this is the same as the sample size). Given the dearth of SNPs relative to substitutions, and in particular the lower diversity level in *D. melanogaster*, we repeated some of these analyses by pooling SNP data across several nearby GC content bins (see Results).

Second, we used the method of Zeng and Charlesworth (2009), modified as described by Evans et al. (2014), which uses the unpolarized SFS (including fixed sites) to infer parameters of a two-allele model with reversible mutation between Wand S alleles, selection and/or gBGC, and changes in population size (see Zeng (2012) for a discussion of the differences between the reversible mutation model and the infinite-sites model on which the method of Glémin et al. (2015) is based). Because this method uses the unpolarized SFS, no outgroup is required. This method can recover signals of selection (and other population genetic parameters) over a longer time scale than the methods of Glémin et al. (2015) because it uses information on the base composition of the species to estimate the parameters (see Zeng and Charlesworth 2009; supplementary fig. S8–S11). As above, we defined W (AT) and S (GC) as our two alleles. We define u as the rate at which S alleles mutate to W alleles, and v as the mutation rate in the opposite direction, and κ=u/v as the mutation bias parameter. To incorporate a change in population size, we assume that the population in the past is at equilibrium with population size $N_{1}$, which then changes instantaneously to $N_{0}$ (this can be either an increase or a reduction in size) and remains in this state for t generations until a sample is taken from the population in the present day (Zeng and Charlesworth 2009; Haddrill et al. 2011; Evans et al. 2014). As with M1* and M1, we also tested the equivalent models where γ=0. For each model, in order to ensure that the true MLE was found, we ran the search algorithm multiple times (typically 500), each initialized from a random starting point. All the results reported above were found by multiple searches with different starting conditions. Chi-squared tests were used to evaluate statistical support for different models. We refer to these models as ZC0 (γ=0) and ZC1 (γ≠0) below. A software package implementing this approach is available at http://zeng-lab.group.shef.ac.uk. For all methods (Zeng and Charlesworth 2009; Glémin et al. 2015), we fitted independent models for each SI and 4-fold bin (Zeng and Charlesworth 2010b; Messer and Petrov 2013).

## Results

### Patterns of Polymorphism and Divergence in the *D. simulans* and *D. m* *elanogaster* Data Sets

For *D. simulans*, after extracting 4-fold degenerate sites and SI (positions 8–30 bp of introns <66-bp long), we retained 7,551 autosomal CDS alignments and 1,226 X-linked CDS alignments, as well as 5,578 autosomal SI alignments and 516 X-linked SI alignments. The final data set contained the reference sequences of *D. simulans, D. melanogaster*, and *D. yakuba*, as well as polymorphism data from 39 *D. simulans* lines, including 21 Madagascan (MD) lines and 18 Kenyan (NS) lines, with 22 of the 39 lines being described for the first time in this article (see Materials and Methods). For *D. melanogaster*, we retained 5,550 autosomal CDS alignments and 888 X-linked CDS alignments, as well as 7397 autosomal SI alignments and 738 X-linked SI alignments, containing polymorphism data from 17 Rwandan (RG) lines, as well as the three reference sequences.

Summary statistics calculated using a *D. simulans* data set that was filtered to separate true genetic variation from variant-calling artefacts are presented in table 1 (see supplementary table S1, Supplementary Material online for the unfiltered data). Consider first the MD lines (*n* = 21) collected from the putatively ancestral range of the species in Madagascar (Dean and Ballard 2004). Autosomal *π* at 4-fold sites (referred to as *π*_4_) was 0.0329 and 0.0317 for the unfiltered and filtered data sets, respectively, similar to the value of 0.035 reported by Begun et al. (2007). On the X, *π*_4_ was 0.0191 and 0.0182 for the two data sets; the Begun et al. (2007) value was 0.02. Tajima’s *D* and $\Delta_{\pi }$ at 4-fold sites are both negative, implying that there may have been a substantial recent population size expansion. Again, values obtained from the filtered and unfiltered data are very similar (cf. table 1 and supplementary table S1, Supplementary Material online). Overall, diversity was slightly reduced for our filtered data set, which may have been a result of more conservative variant filtering criteria, but the differences are minimal. In what follows, we only present results obtained from the filtered data set. SI sites, which we only obtained from our filtered data set, are more diverse than 0-fold and 4-fold sites in the MD population, for both the autosomes (A) (*π*_SI_ = 0.0321) and the X (*π*_SI_ = 0.0208) (table 1).

**Table 1:** Summary statistics for the filtered *D. simulans* data set

Chr.^a^	Site	Within-Population Statistics					Population Differentiation
		Pop.^b^	*π* ^c^	*θ_W_* ^d^	*Δ_π_* ^e^	*D* ^f^	*F_ST_*
A	0-fold^g^	MD	0.0016	0.00269	-0.12	-1.29	0.0202
		NS	0.00148	0.00206	-0.0882	-0.903	
	4-fold^h^	MD	0.0317	0.0434	-0.0784	-1.03	0.0252
		NS	0.0294	0.0347	-0.0457	-0.579	
	SI^i^	MD	0.0321	0.0417	-0.065	-0.603	0.0174
		NS	0.0297	0.0340	-0.036	-0.326	
X	0-fold	MD	0.00119	0.00207	-0.125	-1.27	0.0178
		NS	0.00113	0.00163	-0.0942	-0.924	
	4-fold	MD	0.0182	0.0282	-0.104	-1.31	0.0246
		NS	0.0173	0.0225	-0.0706	-0.847	
	SI	MD	0.0208	0.0298	-0.0924	-0.785	0.0194
		NS	0.0195	0.0248	-0.0591	-0.509	

Note.—All statistics were calculated per gene, and the means are presented here.

a Chromosome.

b Population sample: MD – Madagascar; NS – Kenya.

c Average number of pairwise differences per site between lines.

d Watterson’s estimator of θ, the scaled mutation rate.

e See eq. (1).

f Tajima’s *D*.

g 0-fold degenerate sites.

h 4-fold degenerate sites.

i Sites 8–30 bp of introns <66 bp in length.

The samples collected from Kenya (the NS lines; *n* = 18) have consistently lower diversity levels at 0-fold, 4-fold, and SI sites, and less negative Tajima’s *D* and $\Delta_{\pi }$, probably caused by bottlenecks associated with the colonization process (Dean and Ballard 2004). Nonetheless, *F_ST_* between the two populations at 4-fold sites is rather low: ∼2.5% between NS and MD (table 1), suggesting that there is relatively little genetic differentiation between the ancestral and derived populations. There is also little difference in *F_ST_* at 4-fold sites between the X and A. Similar to the MD population, SI sites are the most diverse class of site as measured by *π* (table 1).

The patterns reported above contrast with those observed in *D. melanogaster* (see table 1 of Jackson et al. 2015). We focus first on samples from the putatively ancestral ranges of both species (i.e., the RG lines for *D. melanogaster*, and the MD lines for *D. simulans*). Autosomal *π*_4_ is ∼2.06 times higher in *D. simulans*, suggestive of higher *N_e_*, which may lead to more effective selection (see Discussion). Tajima’s *D* is also less negative in *D. melanogaster*, with the differences at 4-fold sites being the most noticeable (−0.11 vs. −1.03 for A, and −0.47 vs. −1.31 for the X), suggesting a more stable recent population size in *D. melanogaster*, which is supported by the fits of the Zeng and Charlesworth (ZC) method to the data (see below). The X:A ratio of *π*_4_ in *D. melanogaster* was 1.08, much higher than the expected value of 0.75 under the standard neutral model, whereas it was 0.57 in *D. simulans*. Furthermore, *F_ST_* at 4-fold sites between RG and a sample from France (Jackson et al. 2015) in *D. melanogaster* is ∼10 times higher than that between the MD and NS populations in *D. simulans.* Interestingly, the difference in *F_ST_* between the X and A is much more marked in *D. melanogaster* (0.29 vs. 0.17 for the X and A, respectively) than in *D. simulans* (0.025 for both X and A). Various theories have been proposed to explain differences in diversity levels between X and A, which include sex-specific variance in reproductive success (Charlesworth 2001), demographic effects (Pool and Nielsen 2007; Singh et al. 2007; Pool and Nielsen 2008; Yukilevich et al. 2010), positive and negative selection (Singh et al. 2007; Charlesworth 2012), and differences in recombination rate (Charlesworth 2012). Detailed analyses of the factors underlying X-autosomal differences are outside the scope of this study; below we present results from X and the autosomes separately.

We also assayed divergence between the reference sequences in our alignments. Between *D. melanogaster* and *D. simulans*, *K_A_*, *K_S_* and *K_SI_* were 0.014, 0.109 and 0.130, respectively. These values are similar to those in Table 1 of Parsch et al. (2010) (*K_A_* = 0.019, *K_S_* = 0.106 and *K_SI_* = 0.123), and in Zhang et al. (2013; supplementary table S2 therein) (*K_A_* = 0.015 and *K_S_* = 0.12). In our data *K_A_*, *K_S_* and *K_SI_*, between *D. melanogaster* and *D. yakuba* were 0.036, 0.266 and 0.294, respectively; between *D. simulans* and *D. yakuba*, they were 0.036, 0.250 and 0.302, respectively. Note that divergence is always highest at the SI class of site, which is in agreement with these sites being relatively unconstrained (Halligan and Keightley 2006; Parsch et al. 2010; Clemente and Vogl 2012b). Overall, these patterns suggest that our alignments are of high quality.

In the following sections of this article, we first focus on analysing the forces that act on 4-fold sites. To investigate the relationship between selection and GC content at 4-fold sites (a proxy for the extent of CUB), we binned 4-fold sites by their GC content in the *D. melanogaster* reference sequence, which we used as a proxy for the historic strength of selection favoring GC alleles. In this part of the analysis, the putatively neutrally evolving SI sites are analyzed as a whole and presented alongside results from 4-fold sites for comparison. Later, to gain further insights into the evolution of the SI sites themselves, we binned them according their GC content, and analyzed the bins in the same manner as the 4-fold sites. Only data from the putatively ancestral populations (i.e., MD in *D. simulans* and RG in *D. melanogaster*) are considered, in order to avoid complications introduced by population structure. For ease of notation, we use GC and S (the strong, potentially preferred allele) interchangeably below; the same applies to AT and W (the weak, potentially unpreferred allele).

### Excess of S→W substitutions at 4-Fold sites on both the *D. simulans* and the *D. melanogaster* Lineages

For all the 4-fold site bins and the SI bin (on both A and X), a nonhomogeneous (GTR-NH_b_) substitution model implemented in PAML always fitted the data significantly better than a stationary (GTR) substitution model in both species (min *χ^2 ^*=^* *^166.86, df = 28, *P* **= 1.05 × 10^−^^21^), which is indicative of a nonequilibrium base composition. Considering the genome as a whole, both the *D. melanogaster* and *D. simulans* lineages showed an excess of S→W changes at autosomal and X-linked 4-fold degenerate sites, regardless of which method was employed to infer ancestral states at the *melanogaster-simulans* (*ms*) node (table 2; supplementary table S2, Supplementary Material online; see Materials and Methods). It is evident that the excess is greater in *D. melanogaster* than *D. simulans*. For instance, based on autosomal data obtained by the AWP method, which we expect to be the most accurate method of the three (Matsumoto et al. 2015), the ratio $N_{W\to S}/N_{S\to W}$, where $N_{W\to S}$ and $N_{S\to W}$ are the numbers of substitutions between the S and W alleles along the lineage of interest, is 0.49 in *D. simulans*, but is only 0.26 in *D.* melanogaster (*χ^2 ^*=^* *^2145.8, df = 1, *P* < 0.001). Interestingly, the S→W bias is much more pronounced on the X of *D. melanogaster* with an $N_{W\to S}/N_{S\to W}$ ratio of 0.17, significantly different from the A value of 0.26 (*χ^2 ^*=^* *^212.8, df = 1, *P* < 0.001), whereas in *D. simulans* the ratios are much closer to one another, 0.53 and 0.49, respectively, although this difference is still significant (*χ^2 ^*=^* *^6.97, df  = 1, *P* = 0.008). These results are in line with previous findings of an excess of AT (or unpreferred codon) substitutions at silent sites in *D. melanogaster* (Akashi 1995, 1996; Takano-Shimizu 2001; Akashi et al. 2006). For *D. simulans*, our data are in agreement with a data set curated entirely independently by Juraj Bergman and Claus Vogl (personal communication; supplementary table S2, Supplementary Material online), and suggest that there is a much more pronounced S→W bias than was found in some previous studies (Akashi et al. 2006; Begun et al. 2007; Poh et al. 2012).

**Table 2:** Counts of Substitutions along the *Drosophila melanogaster* and *D. simulans* Lineages at 4-Fold Degenerate and SI Sites

		*D. simulans*				*D. melanogaster*			
		A		X		A		X	
Site^a^	Polarization Method^b^	AT→GC	GC→AT	AT→GC	GC→AT	AT→GC	GC→AT	AT→GC	GC→AT
4-fold	Parsimony	13607	25656	1962	3934	10588	40586	1140	7395
	SBR	14085	30524	2116	4528	11285	47894	1258	8670
	AWP	15219	30945	2450	4639	12399	48264	1425	8611
SI	Parsimony	1859	1598	206	152	1570	1884	131	229
	SBR	1930	2052	231	183	1658	2417	146	271
	AWP	2006	2158	217	206	1718	2506	141	303

a 4-fold – 4-fold degenerate sites; SI – Sites 8–30 bp of introns <66 bp in length.

b The ancestral state at the *melanogaster-simulans* node was determined using three methods: parsimony, the SBR under the GTR-NH_b_ model implemented in PAML, and the average weighted by posterior probability (AWP) under the GTR-NH_b_ model implemented in PAML.

The ratio $N_{W\to S}/N_{S\to W}$ is much closer to unity for SI sites than for 4-fold sites (table 2), which is also in agreement with the previous finding that SI are generally closer to equilibrium than 4-fold sites in both species (Kern and Begun 2005; Singh et al. 2009; Haddrill and Charlesworth 2008; Robinson et al. 2014). The three methods for inferring ancestral states in the *ms* ancestor consistently suggest an AT substitution bias at SI sites in the *D. melanogaster* lineage (table 2). The situation is somewhat more complex in *D. simulans.* For the X, all three methods suggest a mild GC bias, but the ratio based on AWP, which should be the most reliable method of the three (Matsumoto et al. 2015), is not significantly different from 1 (*χ^2 ^*=^* *^0.286, df  = 1, *P* = 0.59). For the autosomes, parsimony suggests a GC bias (*χ^2 ^*=^* *^19.7, df  = 1, *P *=* *0.01), but both SBR and AWP provide some support for a slight AT bias (SBR: *χ^2 ^*=^* *^3.73, df = 1, *P *=* *0.05; AWP: *χ^2 ^*=^* *^5.55, df = 1, *P *=* *0.019) (table 2). This may reflect the tendency for parsimony to overestimate changes from common to rare basepairs (Collins et al. 1994; Eyre-Walker 1998; Akashi et al. 2007; Matsumoto et al. 2015).

### Variation in 4-Fold Site Substitution Patterns across Regions with Different GC Content

**Fig. 1.—:**
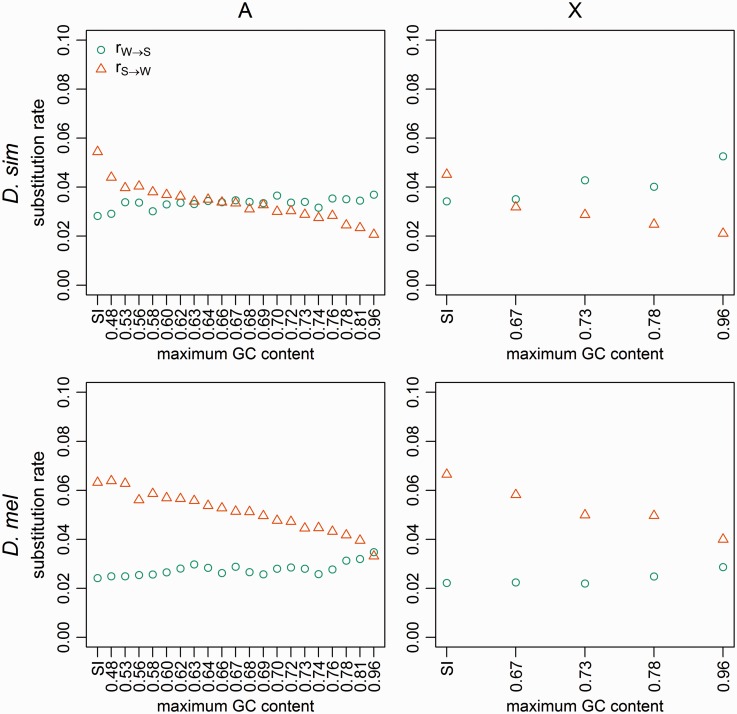
Substitution rates. The results are shown for positions 8–30 bp of introns <66-bp long (SI sites; leftmost points), and 4-fold degenerate sites (remaining points), binned according to the GC content of the extant *D. melanogaster* reference sequence. Rates were calculated for the *D. simulans* lineage (top row) and the *D. melanogaster* lineage (bottom row), for autosomes (left-hand column) and X-linked sites (right-hand column). Teal circles: AT→GC substitutions; orange triangles: GC→AT substitutions.

Under strict neutrality, the substitution rate per site is equal to the mutation rate per site (Kimura 1983). Thus, if 4-fold degenerate sites have never been affected by selection on CUB and/or gBGC, the two substitution rates per site, $r_{W\to S}$ and $r_{S\to W}$, should be uniform across the GC bins, unless there are systematic differences in mutation rates across bins. However, as can be seen from figure 1, in both species, on both the autosomes and the X chromosome, $r_{W\to S}$ is positively correlated with GC content (*D. simulans*, autosomes: Kendall’s *τ* = 0.45, *P *=* *0.006; *D. melanogaster*, autosomes: *τ* = 0.53, *P *=* *0.001). Here and in what follows, we refrain from conducting formal correlation tests of the X-linked data due to the dearth of data points; in addition, data from the SI bins are not included in correlations. In contrast, $r_{S\to W}$ shows a clearly negative relationship with GC content (Kendall’s *τ *= −0.95, *P* < 0.001 and *τ* = −0.96, *P* < 0.001 for *D. simulans* and *D. melanogaster* autosomes, respectively). These patterns are expected if GC alleles (i.e., preferred codons) were favored over AT alleles (i.e., unpreferred codons) for a substantial amount of time along these two lineages, and the intensity of the GC-favoring force increases with GC content (see the Discussion for an explicit model). Also of note is the marked increase in $r_{S\to W}$ relative to $r_{W\to S}$ with GC content in the *D. melanogaster* lineage, which is suggestive of mutations becoming more AT-biased. However, the arguments set out in the Discussion suggest that a change in mutational bias alone is unlikely to explain the data reported here.

**Fig. 2.—:**
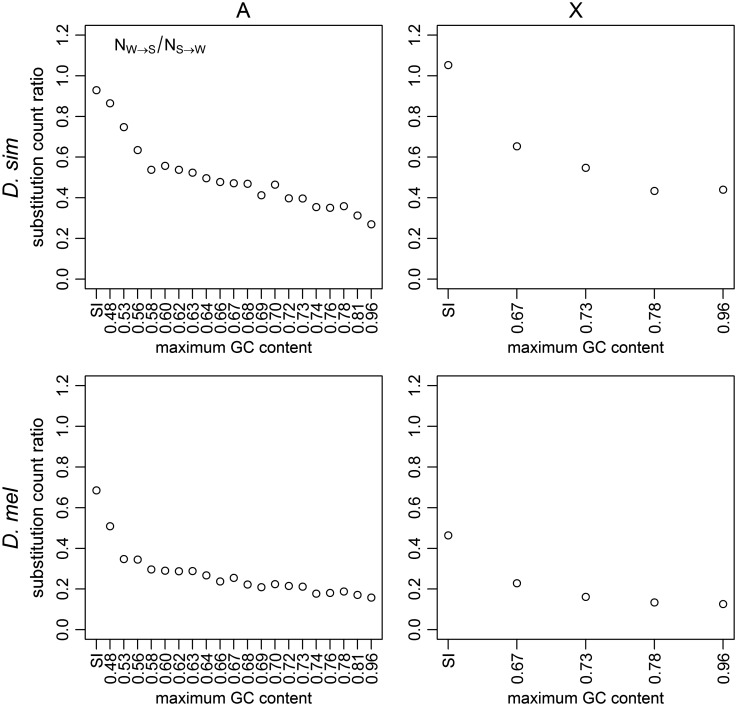
The ratios of substitution counts. The results are shown for positions 8–30 bp of introns <66-bp long (SI sites; leftmost point), and 4-fold degenerate sites (remaining points), binned as described in figure 1. A substitution count ratio of $N_{W\to S}/N_{S\to W}=1$ implies equilibrium base composition. Ratios were calculated for the *D. simulans* lineage (top row) and the *D. melanogaster* lineage (bottom row), for autosomes (left-hand column) and X (right-hand column).

As stated before, the $N_{W\to S}/N_{S\to W}$ ratio at SI sites, particularly in *D. simulans*, is close to unity, the value expected under equilibrium base composition. An investigation across the 4-fold site GC content bins suggests that all of the bins considered here are experiencing some level of AT fixation bias $\left(N_{W\to S}/N_{S\to W}<1\right)$, and that genomic regions with higher GC contents are evolving towards AT faster than regions with lower GC contents. This is clear from the negative correlations between GC content and the level of substitution bias $\left(N_{W\to S}/N_{S\to W}\right)$ calculated per 4-fold site bin in both species (Kendall’s *τ* = −0.96, *P* < 0.001 and* τ* = −0.91, *P* < 0.001 for *D. simulans* and *D. melanogaster* autosomes, respectively) (fig. 2). As explained in the Discussion, this negative correlation can readily be explained by a genome-wide reduction in the intensity of the GC-favoring force.

### DAF at 4-Fold Sites Provide Clear Evidence of Ongoing Selection for Preferred Codons

If selection/gBGC favors GC alleles over AT alleles, then the frequencies of derived GC alleles at AT/GC polymorphic sites (${DAF}_{W\to S}$) should on average be higher than the frequencies of derived AT alleles at AT/GC polymorphic sites (${DAF}_{S\to W}$). Furthermore, ${DAF}_{W\to S}$ should increase as the GC-favoring force becomes stronger (i.e., as 4-fold site GC content increases), whereas ${DAF}_{S\to W}$ should decrease with increasing GC content. In addition, we expect ${DAF}_{neu}$, the DAF for putatively neutral changes (i.e., segregating sites that had mutated from A to T, or vice versa, and from G to C or vice versa), to lie in a position intermediate between ${DAF}_{S\to W}$ and ${DAF}_{W\to S}$ (i.e., ${DAF}_{W\to S}>{DAF}_{neu}>{DAF}_{S\to W}$). In contrast, in a neutral model with a recent increase in mutational bias towards AT, the higher number of derived AT mutations entering the population, which tend to be young and segregate at low frequencies, will depress ${DAF}_{S\to W}$, leading to ${DAF}_{W\to S}$ > ${DAF}_{S\to W}$, but ${DAF}_{neu}$ should be comparable to ${DAF}_{W\to S}$. Moreover, GC content and ${DAF}_{W\to S}$ should be unrelated under this model.

**Fig. 3.—:**
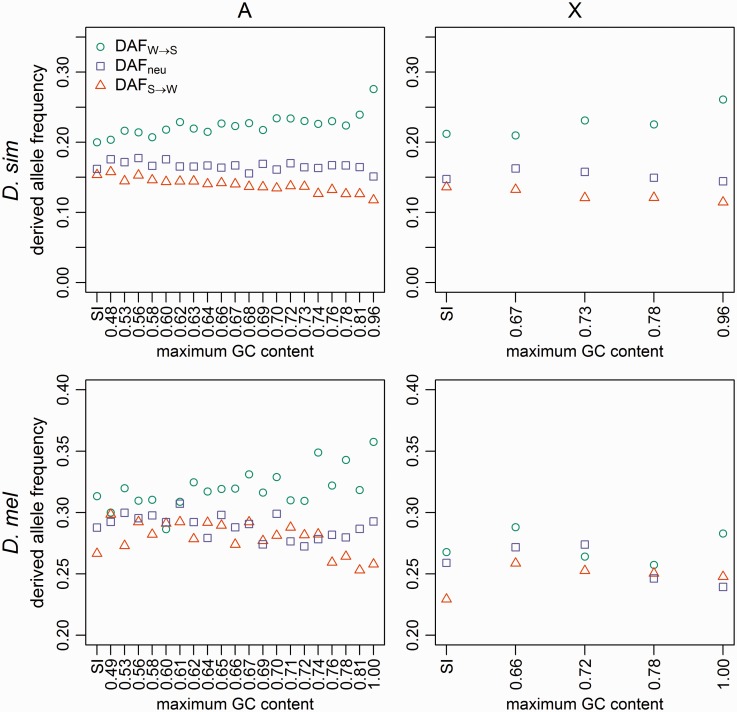
DAF. Mean DAFs are shown for positions 8–30 bp of introns <66-bp long (SI sites; leftmost points), and 4-fold degenerate sites (remaining points), binned as described in figure 1. Mean DAFs were calculated using the MD sample of *D. simulans* (top row) and the RG sample of *D. melanogaster* (bottom row), for autosomes (left-hand column) and X-linked sites (right-hand column). Teal circles: AT→GC mutations; orange triangles: GC→AT mutations; lilac squares: AT→AT mutations or GC→GC mutations.

*D. simulans* fits the expectations of the first model: ${DAF}_{W\to S}$ is greater than ${DAF}_{S\to W}$ in all autosomal and X-linked 4-fold bins, and ${DAF}_{neu}$ is always intermediate between ${DAF}_{W\to S}$ and ${DAF}_{S\to W}$ (fig. 3). Autosomal 4-fold site ${DAF}_{W\to S}$ correlates positively with GC content (Kendall’s *τ* = 0.6, *P* < 0.001; fig. 3), and autosomal 4-fold site ${DAF}_{S\to W}$ correlates negatively with GC content (Kendall’s *τ* = −0.85, *P* < 0.001; fig. 3); data from the X display similar trends. These patterns suggest the action of forces favoring GC over AT alleles in the recent past in this species (a time period of the order of $4N_{e}$ generations), with higher GC content bins experiencing a higher strength of recent selection favoring GC.

In *D. melanogaster*, the equivalent results are less clear. Autosomal ${DAF}_{W\to S}$ is higher than autosomal ${DAF}_{S\to W}$ for 19/20 4-fold bins (fig. 3). As in *D. simulans*, autosomal 4-fold ${DAF}_{W\to S}$ correlates positively with GC content (Kendall’s *τ* = 0.41, *P *=* *0.01; fig. 3), and autosomal 4-fold ${DAF}_{S\to W}$ correlates negatively with GC content (Kendall’s *τ* = −0.47, *P *=* *0.004; fig. 3). ${DAF}_{neu}$ falls between ${DAF}_{W\to S}$ and ${DAF}_{S\to W}$ in 14/20 autosomal 4-fold site bins, but only 1/4 X-linked 4-fold bins (fig. 3). Additionally, the difference between ${DAF}_{W\to S}$ and ${DAF}_{S\to W}$ seems less pronounced than in *D. simulans*, especially on the X chromosome, although on the autosomes the gap between ${DAF}_{W\to S}$ and ${DAF}_{S\to W}$ does tend to increase with GC content and is the largest and most comparable in magnitude to those seen in *D. simulans* in the bins with the highest GC content. Overall, these data provide some evidence of recent selection for GC at 4-fold sites in *D. melanogaster*, but its extent seems to be smaller than in *D. simulans*, and may be restricted to autosomal regions with high GC contents.

### Estimating γ and Other Parameters Using 4-Fold Site Polymorphism Data

To shed further light on the evolutionary dynamics of selection on CUB, we used two different methods for inferring the scaled strength of selection for GC alleles ($\gamma =4N_{e}s$) from polymorphism data. First, we applied the method of Glémin et al. (2015), which detects recent selection (timescale $∼4N_{e}$ generations). We refer to the different variants of this method using the same notation as Glémin et al. (2015). These are model M0, where γ=0 and polarization errors (with respect to inferring ancestral vs. derived alleles) are not taken into account; M1, where γ≠0 and polarization errors are not taken into account; and M0* and M1*, which are the equivalent models after correcting for polarization errors. Second, we used the method of Zeng and Charlesworth (2009), modified as described by Evans et al. (2014), which provides estimates over a longer period. We used two variants of this method, which are referred to as ZC0 (γ=0) and ZC1 (γ≠0).

**Fig. 4.—:**
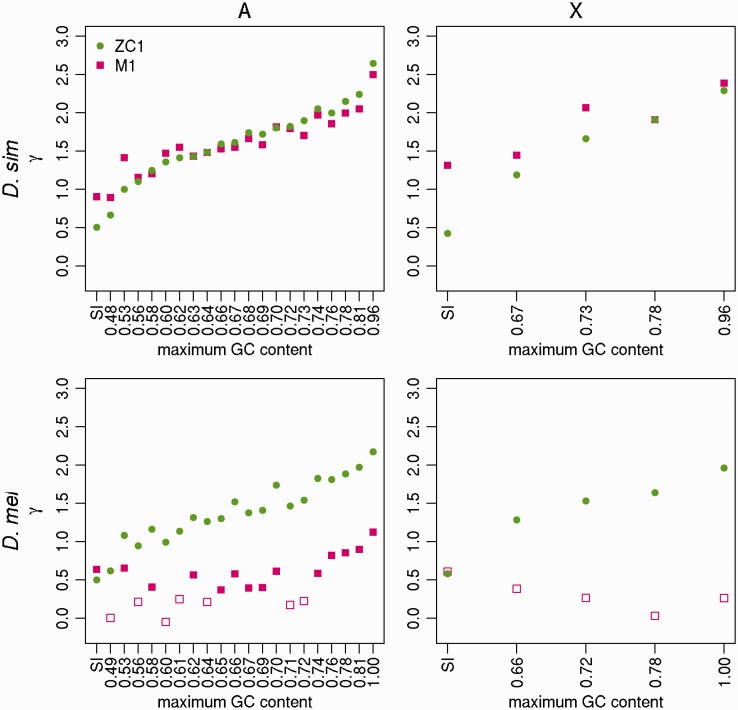
The estimated strength of selection favoring GC alleles. The estimates of the strength of selection in favor of GC alleles ($\gamma =4N_{e}s$) are shown for positions 8–30 bp of introns <66-bp long (SI sites; leftmost points), and 4-fold degenerate sites (remaining points), binned as described in figure 1. γ was estimated using the MD sample of *D. simulans* (top row) and the RG sample of *D. melanogaster* (bottom row), for autosomes (left-hand column) and X-linked sites (right-hand column). Two methods were used: the method of Zeng and Charlesworth (2009) with a one-step size in population size (ZC in the main text) – green circles; and the method of Glémin et al. (2015), not incorporating polarization errors (M1 in the main text)—pink squares. Filled points: bins where a model with γ≠0 fitted best; open points: bins where a model with γ=0 fitted best.

For every *D. simulans* bin on both the A and X, both ZC1 and M1 fit the data significantly better than the corresponding models with γ=0 (i.e., ZC0 and M0; min χ^2 ^=^ ^17.84, df  = 1, *P* < 0.001); the only exception is the X-linked SI bin where M1 does not fit the data better than M0 (χ^2 ^=^ ^0.071, df  = 1, *P *=* *0.79) (fig. 4). Estimates obtained by ZC1 and M1 agree closely for the *D. simulans* data (fig. 4; Wilcoxon paired signed-rank test, *P *=* *0.25). The agreement between the results from the two methods, which are expected to be sensitive to forces favoring GC on different timescales (see Material and Methods), suggests consistent selection over time favoring GC alleles at 4-fold degenerate sites in *D. simulans*. In addition, GC content correlates positively with γ on both the autosomes (Kendall’s *τ *= 0.98, *P* < 0.001; *τ *= 0.88, *P* < 0.001 for ZC1 and M1, respectively) and the X chromosome. Thus, in agreement with the results obtained from the divergence- and DAF-based analyses, selection for GC is indeed stronger in regions with higher GC content. The patterns obtained from comparing M0* and M1* are qualitatively identical (supplementary fig. S2, Supplementary Material online). In addition, when using the Akaike Information Criterion (AIC) to rank the four Glémin models (this is necessary because, e.g., M0* and M1 are not nested and cannot be compared using the likelihood ratio test), M1 and M1* are always the two best fitting models for all bins across both chromosome sets, except for the SI bin on the X (supplementary table S3, Supplementary Material online).

Similarly to the analysis based on DAFs, the patterns are less clear-cut in *D. melanogaster*. When M1 and M0 are compared, 13/20 autosomal 4-fold site bins are found to be non-neutrally evolving, including the four highest autosomal GC bins, and none on the X (fig. 4). In contrast, according to the comparison between M1* and M0*, only 3 autosomal bins show evidence of nonzero γ in *D. melanogaster* (2/20 autosomal 4-fold site bins and the autosomal SI bin), and none of the X-linked bins do so (supplementary fig. S2, Supplementary Material online). In particular, the fact that none of the high GC bins have a significant test is out of keeping with the observation that these bins have large differences between ${DAF}_{W\to S}$ and ${DAF}_{S\to W}$. A close inspection suggests that statistical power may be an issue: there are on average four times fewer SNPs in the 4-fold site bins in *D. melanogaster*, and in the highest 4-fold site bin, there were only 69 W→S SNPs. As described in the Materials and Methods, the Glémin models are parameter rich, especially M0* and M1*. In fact, M1* often came out (e.g., in 10/20 autosomal 4-fold site bins) as the worse fitting one among the four models according to the AIC.

To deal with this issue, we redid the comparison by reducing the number of autosomal 4-fold bins to 10. M1 fits better than M0 in 9/10 bins, while M1* fits better than M0* in 4/10 bins, including two out of the top four GC bins (supplementary fig. S3, Supplementary Material online). According to the AIC, the frequency of M1 being the best fitting model increases to 9/10 bins, whereas the frequency of M1* being the worse fitting model decreases to 2/10 bins (supplementary table S3, Supplementary Material online). The observation that M1* sometimes ranked lower than M1 according to the AIC in both species may also be due to the fact that our method for correcting for nonequilibrium when reconstructing ancestral states has reduced the need to correct for polarization errors.

As is apparent from figure 4, M1 also estimates consistently lower absolute values of γ than ZC1 in *D. melanogaster* (Wilcoxon paired signed-rank test, *P *=* *1.9 × 10^−^^6^). Given that the ZC method returns long-term average estimates of γ, these differences clearly indicate a recent decline in the strength of selection on CUB in this species. As with *D. simulans*, however, autosomal GC content correlates positively with γ under both models (Kendall’s *τ *= 0.87, *P* < 0.001; *τ *= 0.48, *P *=* *0.003 for ZC and M1, respectively; fig. 4), which is suggestive of some, if weak, ongoing selection for GC at autosomal 4-fold sites, particularly in GC-rich regions of the genome. The fact that the SFS is more negatively skewed at 4-fold sites in regions of higher GC content in both species, as measured by Δ_*π*_ (supplementary fig. S4, Supplementary Material online), is also consistent with selection on these sites.

In addition to γ, the two methods also produced estimates of other parameters of interest. For instance, both methods can estimate *κ*, the mutational bias parameter, defined as *u*/*v* where *u* is the mutation rate from *S* to *W* per site per generation, and *v* is that in the opposite direction. As shown in supplementary fig. S12, Supplementary Material online, in *D. simulans*, *κ* is close to 2 across the 4-fold site bins, similar to previous estimates obtained by different methods (Singh et al. 2005; Keightley et al. 2009; Zeng 2010; Schrider et al. 2013). The fact that *κ* is estimated to be similar across the bins suggests that the difference in 4-fold sites’ GC content can be attributed to stronger selection, not to differences in mutational bias. In *D. melanogaster*, the difference in the estimates between the two methods is much more pronounced, with *κ* from the Glémin method (short timescale) being consistently higher than those estimated by the ZC method (long timescale), probably reflecting a recent increase in the mutation rate towards A/T nucleotides (see Discussion).

Consistent with the apparently negative Tajima’s *D* values calculated using 4-fold sites in *D. simulans* (table 1), the ZC method detected clear evidence for recent population expansion in all bins (*P* < 10^−^^16^ for all bins; supplementary table S4, Supplementary Material online), whereas for *D. melanogaster*, no clear evidence for recent population expansion was found, which is consistent with the observed data (e.g., Tajima’s *D* is only −0.11 for A in *D. melanogaster*, but is −1.03 in *D. simulans*) and our previous analysis based on a different data set (Zeng and Charlesworth 2009). In supplementary text S2, Supplementary Material online (see also supplementary tables S5 and S6, Supplementary Material online), we present a more detailed description of estimation of the demographic parameters in *D. melanogaster*, and the statistical and computational issues we encountered. We also provide evidence that our conclusion of a continuing decline in selection intensity in *D. melanogaster* is robust to these potential issues (supplementary fig. S13, Supplementary Material online).

### A More Detailed Analysis of the SI

The SI data shown in figures 3 and 4 suggest that GC may be favored over AT in SI. Given the apparent lack of selective constraints on SI sites (Halligan and Keightley 2006; Parsch et al. 2010), this is suggestive of the action of gBGC. In contrast to selection on CUB at 4-fold sites, all alleles have equal fitness under the gBGC model, and the selection-like pattern is created by the preferential transmission of the *S* allele in *SW* heterozygotes to the next generation (Duret and Galtier 2009). The S→S and W→W mutations are “neutral” in the sense that they should be unaffected by gBGC. To gain further insights, we carried out additional analyses by binning the SI data according to their GC content, and asked whether gBGC could be responsible for the observed patterns. Constrained by the limited amount of data and the parameter-richness of some of the models, we only carried out these analyses using the autosomal SI data, divided into five bins. These data were then examined in the same way as the 4-fold sites. However, with such a small number of bins, the correlation-based analysis is likely to be prone to statistical noise; the results should thus be treated with caution.

**Fig. 5.—:**
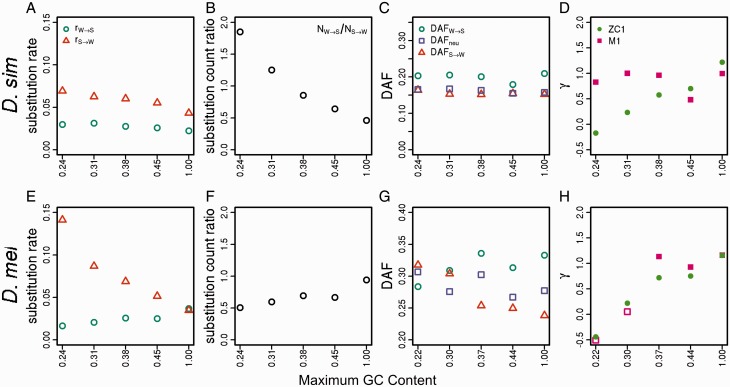
Results for autosomal SI sites binned by GC content. Top row: data from the MD sample of *D. simulans*; bottom row: data from the RG sample of *D. melanogaster*. *A* and *E*: substitution rates for AT→GC substitutions (teal circles) and GC→AT substitutions (orange triangles). *B* and *F*: the ratio of substitution counts along each lineage. *C* and *G*: DAF for AT→GC mutations (teal circles); GC→AT mutations (orange triangles); AT→AT mutations or GC→GC mutations (lilac squares). AT→AT and GC→GC mutations were labelled as neutral to signify that they should be unaffected by gBGC. *D* and *H*: estimated values of the magnitude of selection in favor of GC alleles ($\gamma =4N_{e}s$). Two methods were used: the method of Zeng and Charlesworth (2009) with a one-step size in population size (ZC in the main text)—green circles; and the method of Glémin et al. (2015), not incorporating polarization errors (M1 in the main text)—pink squares. Filled points: bins where a model with γ≠0 fitted best; open points: bins where a model with γ=0 fitted best. All analyses that required reconstruction of the ancestral state at the *ms* node used the AWP method, as described in the main text.

As shown in figure 5*A* and *E*, $r_{S\to W}$ decreases as GC content increases in both species (Kendall’s *τ* = −1, *P *=* *0.03), which may reflect an ancestral reduction in the strength of the force favoring G/C nucleotides (see Discussion). However, $r_{W\to S}$ is not significantly correlated with GC content in either species (Kendall’s *τ* = −0.8, *P *=* *0.09, in *D. simulans*; Kendall’s *τ* = 0.8, *P *=* *0.09, in *D. melanogaster*). Comparing $N_{W\to S}$ and $N_{S\to W}$ across bins using a 2 × 5 contingency table test suggests that the substitution pattern is heterogeneous across the bins in both species (*P* < 2.2 × 10^−^^16^ in *D. simulans* and *P *=* *2.04 × 10^−^^8^ in *D. melanogaster*). The $N_{W\to S}$/$N_{S\to W}$ ratio decreases with increasing GC content in *D. simulans* (Kendall’s *τ* = −1, *P *=* *0.03; fig. 5*B*), qualitatively similar to what we reported above for the 4-fold sites in this species (fig. 2). However, this ratio shows no significant correlation with GC content in *D. melanogaster* (Kendall’s *τ* = 0.8, *P *=* *0.09; fig. 5*F*). These results highlight the difficulty in conducting detailed analyses in the SI regions, due to insufficient data. Nevertheless, they provide evidence for variation between different SI regions.

We did not detect any statistically significant correlation between the three types of DAFs and GC content in *D. simulans* (fig. 5*C*, minimum *P *=* *0.22 for the three tests), although the relationship ${DAF}_{S\to W}$ < ${DAF}_{neu}$ < ${DAF}_{W\to S}$ holds in all bins. The lack of strong support for a relationship with GC content was also reflected when the Kruskal–Wallis test was used to test for heterogeneity in median DAFs across bins; the *p*-values for S→W, neutral, and W→S are 0.38, 0.20 and 0.04, respectively. In *D. melanogaster* (fig. 5*G*), ${DAF}_{S\to W}$ is significantly negatively correlated with GC content (Kendall’s *τ* = −1, *P *=* *0.03), but no relationship was found for the other two DAFs (minimum *P *=* *0.22). In the three bins with higher GC content, we have ${DAF}_{S\to W}$ < ${DAF}_{neu}$ < ${DAF}_{W\to S}$. But the order is completely reversed in the lowest GC content bin, although the differences between the DAFs are nonsignificant based on the Glémin model (see below). Consistent with this, the Kruskal–Wallis test detected significant heterogeneity in median DAF across bins in the ${DAF}_{S\to W}$ case (*P *=* *1.40 × 10^−^^8^), but not in the other two cases (*P* > 0.08).

Finally, we used polymorphism data to estimate the strength of the force favoring GC, as measured by *γ*. In line with the DAF-based analysis, in neither *D. simulans* (Kendall’s *τ* = 0, *P *=* *1; fig. 5*D*) nor *D. melanogaster* (Kendall’s *τ* = 0.8, *P *=* *0.09; fig. 5*H*) did we find a significant relationship between GC content and *γ* as estimated by the M1 model of Glémin et al. (2015). In *D. simulans*, M1 fits the data significantly better than M0 in all five bins, whereas in *D. melanogaster*, the neutral model M0 is sufficient to explain the data for the first two bins, with the M1 model being more adequate for data collected from the more GC-rich bins. Estimates of *γ* produced by the ZC1 method are positively correlated with GC content in both species (Kendall’s *τ* = 1, *P *=* *0.03; fig. 5*D* and *H*). Interestingly, ZC1 fits the data significantly better than ZC0 in all cases, even in bins where *γ* is fairly close to zero. A close inspection suggests that this is not due to poor convergence in the search algorithm. Furthermore, simulations have shown that the ZC model is very robust to linkage between sites and demographic changes (Zeng and Charlesworth 2010b), suggesting that these results are unlikely to be methodological artefacts, and may reflect long-term dynamics in these regions. Finally, in *D. melanogaster*, there is no clear evidence that the estimates of long-term *γ* derived from ZC1 are higher than estimates of short-term *γ* derived from M1 (fig. 5*H*).

## Discussion

### Evidence for Past Selection on CUB in Both *Drosophila* Species

The correlations between the substitution rates and GC content at 4-fold sites presented in figure 1 and supplementary fig. S5, Supplementary Material online can be explored using the following modelling framework (Li 1987; Bulmer 1991; McVean and Charlesworth 1999), which assumes a fixed $N_{e}$ and thus a fixed value of γ for each GC bin. If there are temporal changes along a lineage, we can regard these parameters as long-term averages. Let u be the mutation rate from S→W per site per generation; and v be that in the opposite direction. Define κ as u/v. The two substitution rates, $r_{S\to W}$ and $r_{W\to S}$, are proportional to $u\gamma /\left[\text{exp}\;\left(\gamma \right)-1\right]$ and $v\gamma /\left[1-\text{exp}\;\left(-\gamma \right)\right]$, respectively (e.g., Eq. B6.4.2b of Charlesworth and Charlesworth (2010); Eq. 11 of Sawyer and Hartl (1992); Akashi et al. 2007). We can then define

(2) $$R=\frac{r_{S\to W}}{r_{W\to S}}=\kappa \frac{1-e^{-\gamma }}{e^{\gamma }-1}=\kappa e^{-\gamma }$$

Assuming that u and v are constant across the GC bins and over time (κ is thus also constant), R is a function of γ. Taking the derivative with respect to γ, we have

(3) $$\frac{dR}{d\gamma }=-\kappa e^{-\gamma }$$

In other words, R=κ when γ=0 (neutrality), and decreases as γ becomes positive (i.e., when W is selected against). Thus, the decreasing values of R shown in figure 1 and supplementary fig. S5, Supplementary Material online suggest that S is more strongly favoured in high GC bins. For instance, the R values for the lowest and highest autosomal 4-fold site bins in *D. simulans* are 1.51 and 0.56, respectively. If the SI sites are neutral (see below), κ can be estimated by the *R* value from the SI bin, which is 1.93, very close to the value of 2 reported previously (Singh et al. 2005; Keightley et al. 2009; Zeng 2010; Schrider et al. 2013), solving eq. (2) for γ gives values of 0.25 and 1.24 for the lowest and highest bins, respectively. These rough, long-term estimates are about 2-fold lower than those obtained from the polymorphism data (fig. 4). It is possible that *D. simulans* has a larger recent $N_{e}$ (reflected in the polymorphism-based analysis) than the average $N_{e}$ along the entire lineage, which is consistent with the evidence for population expansion from the negative Tajima’s *D* values (table 1). Finally, as detailed in the supplementary text S1, Supplementary Material online, this model can also explain why the slope for $r_{S\to W}$ is apparently steeper than that for $r_{W\to S}$ (fig. 1).

The above model can also explain why, at 4-fold sites, $R_{N}=N_{W\to S}/N_{S\to W}$ < 1 and there is a negative relationship between $R_{N}$ and GC content (fig. 2), where $N_{W\to S}$ and $N_{S\to W}$ are the numbers of substitutions between the S and W alleles along the lineage of interest. Note first that $N_{S\to W}$and $N_{W\to S}$ are, respectively, proportional to $Qu\gamma /\left[\;\text{exp}\;\left(\gamma \right)-1\right]$ and $\left(1-Q\right)v\gamma /\left[1-\text{exp}\;\left(-\gamma \right)\right]$, where Q is the GC content at the *ms* node (since Q changes very slowly, this should be a reasonable first approximation). At equilibrium, $Q=1/\left[1+\kappa \;\text{exp}\;\left(-\gamma \right)\right]$ (Li 1987; Bulmer 1991) and hence $N_{W\to S}/N_{S\to W}=1$. Consider a model where the ancestral species was at equilibrium, but γ is reduced to $p\gamma \left(0\leq p<1\right)$ along a lineage that leads to an extant species, so that $N_{S\to W}$and $N_{W\to S}$ become proportional to $Qup\gamma /\left[\text{exp}\;\left(p\gamma \right)-1\right]$ and $\left(1-Q\right)vp\gamma /\left[1-\text{exp}\;\left(-p\gamma \right)\right]$, respectively. Then, $R_{N}$ for the GC content bin in question can be written as

(4) $$R_{N}=\frac{N_{W\to S}}{N_{S\to W}}=\frac{\left(1-Q\right)(e^{p\gamma }-1)}{\kappa Q(1-e^{-p\gamma })}=e^{-(1-p)\gamma }$$

Assuming that p is constant across bins (i.e., there has been a genome-wide proportional reduction in γ), then $R_{N}$ decreases as γ increases. This, together with the arguments presented above that the long-term average γ is higher in high GC bins, eq. (4) implies that the negative relationship between $R_{N}$ and GC content is consistent with a genome-wide reduction in the intensity of selection in both species (see also Akashi et al. 2007).

In contrast, if we assume that γ=0 and κ is constant across the bins (i.e., there has been no selection along both the *D. melanogaster* and *D. simulans* lineages), the fact that R=κ means that a genome-wide increase in κ (i.e., a more AT-biased mutation pattern) would not cause a negative relationship between R and GC content. If the relationship between R and GC content were entirely mutational in origin, then u must decrease as GC content increases, whereas v changes in the opposite direction (fig. 1). Such a model is incompatible with the evidence for selection from the two polymorphism-based methods (fig. 4), and cannot easily explain the well-known positive correlation between GC content of CDSs (or the extent of CUB) and gene expression levels (e.g., Campos et al. 2013), especially when considering the lack of support for transcription-coupled mutational repair in *Drosophila* (Singh et al. 2005; Keightley et al. 2009).

As shown in supplementary fig. S12, Supplementary Material online, the Glémin method (short timescale) and the ZC method (long timescale) returned κ estimates that are more comparable in *D. simulans* than in *D. melanogaster*; the ZC method produced consistently lower estimates in *D. simulans* and consistently higher estimates in *D. melanogaster* (two-sided binomial test, *P *=* *1.91 × 10^−^^6^ in both cases). Taken at face value, these results suggest that there probably has been relatively little change in the extent of mutational bias in the *D. simulans* lineage, whereas mutation may have become more AT-biased in *D. melanogaster*. These results suggest that the patterns shown in figure 2 are probably a result of an ancestral reduction in the efficiency of selection in *D. simulans*. For *D. melanogaster*, it is possible that a more AT-biased mutational pattern has also contributed to the evolution of base composition in its genome, as suggested by previous studies (Takano-Shimizu 2001; Kern and Begun 2005; Nielsen et al. 2007; Zeng and Charlesworth 2010a; Clemente and Vogl 2012b).

Overall, the above considerations suggest that the data presented in figures 1 and 2 and supplementary fig. S5, Supplementary Material online cannot be explained by a shift towards a more AT-biased mutational pattern alone. Instead, selection favoring GC over AT basepairs must have acted on both species for a significant amount of time since they last shared a common ancestor, although both lineages are likely to have experienced an ancestral reduction in the efficacy of selection that led to the AT-biased substitution patterns.

### Estimating the Intensity of Selection on Preferred Codons over Different Timescales

A novelty of this study is that, by applying two different methods to the same polymorphism data set, we have attempted to understand how the selective pressure on CUB has changed over time by comparing γ estimates reflective of either a short timescale (for roughly the last 4*N_e_* generations; i.e., the Glémin method (Glémin et al. 2015)), or a long timescale (for > 4*N_e_* generations; i.e., the ZC method; Zeng and Charlesworth 2009). However, pinpointing the exact timescale for the ZC method is difficult, because it depends on details of past evolutionary dynamics that we know little about (e.g., the timescale can be affected by both the time when the ancestral population size reduction took place and the severity of the reduction; see supplementary figs. S8–S11 in Zeng and Charlesworth 2009). This difference in timescale between the methods is due to the use of the derived SFS under the infinite-sites model (Kimura 1983) in the Glémin method and the use of a reversible mutation model in the ZC method (see Zeng 2012 for a more thorough discussion of the differences between these two models). By the same token, we can classify other polymorphism-based methods into short timescale (Akashi and Schaeffer 1997; Bustamante et al. 2001) and long timescale (Maside et al. 2004; Cutter and Charlesworth 2006; Galtier et al. 2006; Zeng 2010; Clemente and Vogl 2012a; Vogl and Bergman 2015).

Contrasting the results obtained from the ZC method with those from the divergence-based analysis (figs. 1 and 2) and the Glémin method (fig. 4) is informative. First, consider *D. simulans*. The fact that values of γ estimated by both the ZC method and the Glémin method are virtually identical suggests that there have not been significant changes in the intensity of selection over the time period that the ZC method considers. Hence, the reduction in γ suggested in the previous section, which may have caused $N_{W\to S}/N_{S\to W}<1$ and the negative correlation between $N_{W\to S}/N_{S\to W}$ and GC content, probably happened so early during the evolution of *D. simulans* that it did not leave detectable traces in the polymorphism data.

In contrast, in *D. melanogaster*, both the divergence-based analysis and the comparison between the ZC method and the Glémin method provide evidence for a reduction in γ, indicating a recent decline in this species. Assuming that SI are neutral, and using autosomal data from the putatively ancestral populations (i.e., MD and RG), table 1 in this study and table 1 in Jackson et al. (2015) suggest that $N_{e}$ is 2.21-fold higher in *D. simulans* compared with *D. melanogaster*, implying more efficient selection in the recent past. In fact, focusing on the 13 autosomal 4-fold site bins in *D. melanogaster* where M1 fits the data better than M0 (filled squares in fig. 4), the γ estimates in the corresponding bins in *D. simulans* are on average 2.93 times higher, comparable to the difference in $N_{e}$ suggested by the SI data. This difference in $N_{e}$ may be due to differences in the two species’ demographic history. Previous studies have also suggested that the lower recombination rate in *D. melanogaster* compared with *D. simulans* (Comeron et al. 2012; True et al. 1996) may have played a role through stronger Hill–Robertson interference between selected sties (Takano-Shimizu 1999; McVean and Charlesworth 2000; Comeron et al. 2008, 2012; Cutter and Payseur 2013). However, without detailed genetic maps from closely-related outgroup species, it is impossible to ascertain whether the reduced map length in *D. melanogaster* represents the ancestral or derived state; this is an important area for further research.

### Comparison with Previous Studies

Poh et al. (2012) suggested that AT-ending codons might be favored in *D. melanogaster*, based on the observation that, along the *D. melanogaster* lineage, S→W mutations fixed at a higher rate than W→S changes; also, in their polymorphism data set, the proportion of singletons in the SFS for S→W changes was smaller than in the SFS for W→S changes (23.2% vs. 24.3%). The latter difference is significant under a Mann–Whitney *U* test, although neither Tajima’s *D* nor Fu and Li’s D* were significantly different from zero. Here we have provided evidence that the pattern of $r_{S\to W}$ > $r_{W\to S}$ can be readily explained by a reduction in selection intensity favoring *S* basepairs along the *D. melanogaster* lineage. As for their polymorphism data, Poh et al. (2012) used lines collected from Raleigh, North America. There is clear evidence that this population has experienced bottlenecks in the recent past, as can be seen from the lower level of diversity in this population compared with populations from Africa (genome-wide *π_S_* = 0.013 vs. 0.019 for the Raleigh and Malawi populations; Langley et al. 2012). Without using model-based methods to correct for the effects of demographic changes, the results of Poh et al. (2012) may be susceptible to complications caused by such complex demography. In addition, their ancestral states were inferred using maximum parsimony, which is prone to error. In supplementary fig. S14, Supplementary Material online, we used parameter values realistic for *D. melanogaster* to show that, with demography and polarization error, it is possible for the proportion of singletons in the SFS for S→W changes to be lower than that for W→S changes in the presence of weak selection favoring *S* (see the legend to supplementary fig. S14, Supplementary Material online for further discussion of this issue).

Another possible cause of the Poh et al. (2012) results is admixture with African *D. melanogaster* during the recovery from the bottleneck (Caracristi and Schlötterer 2003; Duchen et al. 2013; Bergland et al. 2016). Because the average synonymous site GC content is >60% (Campos et al. 2013) and mutation is AT-biased (supplementary fig. S12, Supplementary Material online), S→W SNPs should be more common overall among the introduced variants than W→S SNPs. Rapid population growth following the bottleneck would make the introduced S→W variants contribute more multiple copies of the derived *W* alleles than W→S variants, which could create the relative deficit of W→S singletons. Because this effect is expected to be stronger in regions with higher GC content, it could also explain Poh et al.’s (2012) observation that the relative deficit of S→W singletons is more apparent in highly-expressed genes.

A detailed analysis of these demographic factors is beyond the scope of this article, as it would require knowledge of many poorly-known parameters (for example, the time and the extent of the admixture; see Duchen et al. 2013). Overall, notwithstanding the possibility that AT-ending codons may be favored in some genes (DuMont et al. 2004; Nielsen et al. 2007), our data from a nonbottlenecked population that is close to the putative ancestor of *D. melanogaster* suggest that the genome-wide pattern is compatible with a model in which selection on CUB is reduced in the *D. melanogaster* lineage and ongoing selection is confined to the most GC-rich parts of the genome.

In addition, Lawrie et al. (2013) suggested that a subset of 4-fold sites may be under strong selective constraints in *D. melanogaster*. These authors based their conclusions on two main observations that were made from analysing a North American population generated by the *Drosophila* Genetic Reference Panel (DGRP): a lack of difference in the shape of the SFSs between 4-fold and SI sites and a ∼22% reduction in diversity level at 4-fold sites relative to SI sites (after correcting for differences in GC content; see their fig. 1). The authors suggested that their findings might represent “a largely orthogonal force to canonical CUB” (p. 12 of Lawrie et al. (2013)). Indeed, by using a sample with 130 alleles, they were able to detect signals of much stronger purifying selection (with γ estimated to be −283) than is permitted by our sample sizes (21 MD lines from *D. simulans* and 17 RG lines from *D. melanogaster*). Additionally, their estimates of the intensity of strong selection appear to be uniform across genes with high and low levels of CUB, in contrast to the pattern we report here.

Obtaining more information about these two seemingly independent forces acting on 4-fold sites (weak selection on CUB and strong purifying selection) is an important area for future investigation. Several factors are of note. As discussed above, admixture is likely to complicate the analysis of the North American population of *D. melanogaster*. Although Lawrie et al. (2013) used the same method as that of Glémin et al. (2015) to control for demography, this method is nonetheless an approximation and may still lead to biased estimates of γ under certain conditions, as demonstrated by simulations (Eyre-Walker et al. 2006). Using nonadmixed populations and explicit demographic models (as in this study) may be preferable. Second, with a larger sample size (as in Lawrie et al. (2013)), it should be possible to jointly model the effects of both weak selection on CUB, which requires distinguishing W→S, S→W, and putatively neutral mutations (i.e., S→S and W→W) (which were ignored by Lawrie et al. 2013), and strong purifying selection, which primarily leads to an excess of very low frequency variants. By doing so, we should be able to explicitly test the relative importance of these two forces, and gain further insights into the evolution of 4-fold sites in the *Drosophila* genome.

### Complex Evolutionary Patterns in Short Introns

Short introns have been widely used as a neutral reference in *Drosophila* evolutionary genetic studies (Halligan and Keightley 2009; Parsch et al. 2010), and are thought to be closer to base composition equilibrium than other genomic regions (Kern and Begun 2005; Haddrill and Charlesworth 2008; Singh et al. 2009; Robinson et al. 2014), a pattern we have also observed (fig. 2). When analyzed as a whole, the data point to the existence of a GC-favoring force in both species (figs. 3 and 4). Given the apparent lack of selective constraints in SI regions, it seems probable that gBGC may have played a significant role in their evolution. Although our detailed analyses were complicated by insufficient data, multiple aspects of the data presented in figure 5 are nonetheless inconsistent with the standard gBGC model, which would predict that the strength of the GC-favoring effect should increase with GC content (Duret and Galtier 2009).

For *D. simulans*, the substitution patterns across SI bins shown in figure 5 are qualitatively similar to those shown in figures 1 and 2 for the 4-fold sites. This seems to imply that the GC-favoring force acting on short introns may also have experienced a reduction in strength. However, in contrast to the 4-fold sites for which a genome-wide excess of S→W substitutions was observed (fig. 2), we obtained contrasting patterns in low-GC and high-GC SI bins (fig. 5B), with the former having a significant bias towards W→S substitutions (χ^2^ test, *P *=* *2.30 × 10^−^^16^), and the latter a significant bias towards S→W substitutions (χ^2^ test, *P *=* *2.95 × 10^−^^24^). These contrasting patterns could potentially be explained by an increase in the strength of the GC-favoring force in the low-GC short introns, but a decrease in the high-GC ones. The difference between the *γ* values estimated by the Glémin method and the ZC method gives some tantalising indications that this might have happened (fig. 5D). However, we are unaware of any direct evidence supporting this possibility, and it is also hard to reconcile with what we observed at the 4-fold sites, which were extracted from the same set of genes. Furthermore, the Glémin model provides little evidence that *S* basepairs are more favored in high GC content regions, although this might have been the case in the past according to the ZC model.

In *D. melanogaster* (fig. 5F), a bias towards fixing *W* basepairs was observed in the first four SI bins (χ^2^ test, maximum *P *=* *5.85 × 10^−^^8^), but not the last bin (χ^2^ test, *P *=* *0.40). Again this is inconsistent with the genome-wide fixation bias towards *W* at the 4-fold sites (fig. 2). Estimates of *γ* from the two polymorphism-based methods are closer to each other compared with *D. simulans*, and both methods seem to suggest that *S* basepairs are more favored in GC-rich regions (fig. 5H), but the small number of bins makes it difficult to draw definitive conclusions from correlation-based analyses.

To investigate this further, we calculated the polymorphism-to-divergence ratio for W→S changes, S→W changes, and changes that are supposedly unaffected by gBGC (i.e., W→W and S→S changes), denoted by ${rpd}_{W\to S}$, ${rpd}_{S\to W}$, and ${rpd}_{neu}$, respectively. If high GC content is driven by gBGC, we expect ${rpd}_{neu}$/${rpd}_{W\to S}$ > 1 (i.e., fixation bias towards *S*) and ${rpd}_{neu}$/${rpd}_{S\to W}$ < 1 (i.e., fixation bias against *W*) in high GC bins, but these two ratios should be close to one in low GC bins where gBGC should be weak. In *D. melanogaster*, the first prediction was met (${rpd}_{neu}$/${rpd}_{W\to S}$ = 1.60, *P *=* *0.001 and ${rpd}_{neu}$/${rpd}_{S\to W}$ = 0.69, *P *=* *7.2 × 10^−^^3^, in the most GC-rich bin). However, we found evidence for the existence of an AT-favoring force in the bin with the lowest GC content (${rpd}_{neu}$/${rpd}_{W\to S}$ = 0.66, *P *=* *7.60 × 10^−^^5^, and ${rpd}_{neu}$/${rpd}_{S\to W}$ = 2.01, *P *=* *3.50 × 10^−^^12^), which is in agreement with estimates produced by the ZC method (fig. 5*H*), but inconsistent with the gBGC model. In a similar analysis of the SI bins in *D. simulans*, none of the polymorphism-to-divergence ratios were found to be significantly different from 1, except in the bin with the lowest GC content where ${rpd}_{neu}$/${rpd}_{W\to S}$ = 1.25 (*P *=* *0.0079). These findings are again inconsistent with the gBGC model.

Overall, the data from both species suggest that there is heterogeneity in evolutionary patterns between short introns residing in different parts of the genome, and that there might be some GC-favoring forces acting on short introns. However, there are substantial uncertainties as to how much of the GC-favoring effect is caused by gBGC. This conclusion is consistent with several previous studies that found little or no evidence for gBGC in *D. melanogaster* (Clemente and Vogl 2012b; Comeron et al. 2012; Campos et al. 2013; Robinson et al. 2014). Furthermore, in contrast to the 4-fold sites, where a reduction in *γ* is clear when estimates from the Glémin model and the ZC model are compared, no clear evidence of such a difference can be seen in the SI data. Regardless, this GC-favoring force acting on short introns is unlikely to be the sole explanation of the results obtained from 4-fold sites, because the γ estimates obtained from the latter are consistently higher than those from the former (fig. 4 vs. fig. 5). Given the importance of these putatively neutral sites in short introns, more work is necessary to understand the unique features reported above. 

## Supplementary Material

Supplementary data are available at *Genome Biology and Evolution* online.

## Supplementary Material

Supplementary Data
