# Supplementary material for: Variation in the Intensity of Selection on Codon Bias over Time Causes Contrasting Patterns of Base Composition Evolution in Drosophila
Source: Genome Biol Evol. 2017 Jan 12;9(1):102–23. doi: 10.1093/gbe/evw291 (PMC5381600; doi:10.1093/gbe/evw291)
Supplement: Supplementary Data [file evw291_Supp.zip › Dsim_supplement_tables_S4-S6.docx]

Table S4. Parameters from the ZC models for *D. simulans*

| Model^a^ | Chr^b^ | Bin^c^ | Ln-likelihood | theta_10^d^ | theta_01^d^ | Gamma^e^ | rho_1^f^ | tau_1^g^ |
| --- | --- | --- | --- | --- | --- | --- | --- | --- |
| Size Change | A | SI | -129510.8 | 0.009063231 | 0.02665429 | 0.5053996 | 7.132612 | 0.09510645 |
|  |  | 1 | -64627.64 | 0.007925471 | 0.01999906 | 0.6654698 | 6.730757 | 0.08962270 |
|  |  | 2 | -90289.84 | 0.010609965 | 0.02494194 | 1.0003426 | 7.029799 | 0.09041692 |
|  |  | 3 | -105893.06 | 0.011778773 | 0.02675734 | 1.1016389 | 6.475094 | 0.09039624 |
|  |  | 4 | -107839.44 | 0.011592866 | 0.02730390 | 1.2476840 | 7.209585 | 0.08127633 |
|  |  | 5 | -101560.40 | 0.012552112 | 0.03006254 | 1.3595118 | 6.848893 | 0.07557119 |
|  |  | 6 | -108143.39 | 0.013797508 | 0.03224679 | 1.4120030 | 7.156593 | 0.06237867 |
|  |  | 7 | -105746.15 | 0.013548504 | 0.03021001 | 1.4292874 | 6.760338 | 0.07152871 |
|  |  | 8 | -97464.79 | 0.013057692 | 0.02886844 | 1.4828819 | 7.652300 | 0.06283991 |
|  |  | 9 | -95475.83 | 0.012293829 | 0.02870964 | 1.5931675 | 7.029213 | 0.07515016 |
|  |  | 10 | -96808.99 | 0.012491374 | 0.02827678 | 1.6124739 | 7.123327 | 0.07405093 |
|  |  | 11 | -85982.50 | 0.012106197 | 0.02914640 | 1.7386829 | 7.198196 | 0.07250917 |
|  |  | 12 | -89065.61 | 0.011890260 | 0.02686627 | 1.7200913 | 7.742159 | 0.07267078 |
|  |  | 13 | -89781.72 | 0.012725083 | 0.02904295 | 1.8037104 | 7.894146 | 0.05811314 |
|  |  | 14 | -81394.42 | 0.014140724 | 0.03083336 | 1.8215664 | 6.784793 | 0.06735427 |
|  |  | 15 | -80527.71 | 0.012830382 | 0.02849215 | 1.8968239 | 7.934777 | 0.05497307 |
|  |  | 16 | -70057.60 | 0.012032862 | 0.02861499 | 2.0529023 | 7.628394 | 0.06451422 |
|  |  | 17 | -69437.71 | 0.013143399 | 0.02721952 | 1.9984671 | 7.154343 | 0.07527195 |
|  |  | 18 | -60311.44 | 0.011851515 | 0.02529495 | 2.1476693 | 7.080975 | 0.07581237 |
|  |  | 19 | -50125.71 | 0.013357308 | 0.02640404 | 2.2404037 | 9.653536 | 0.04349114 |
|  |  | 20 | -37689.44 | 0.011965293 | 0.02532356 | 2.6445748 | 9.079092 | 0.04473185 |
|  | X | SI | -11801.07 | 0.006666192 | 0.01401326 | 0.4239841 | 10.30722 | 0.07332561 |
|  |  | 1 | -64611.02 | 0.006083731 | 0.01192166 | 1.188950 | 13.76701 | 0.04751119 |
|  |  | 2 | -67048.26 | 0.007927229 | 0.01547754 | 1.661858 | 14.17604 | 0.04206629 |
|  |  | 3 | -55250.59 | 0.008121589 | 0.01498454 | 1.908711 | 14.89628 | 0.03708429 |
|  |  | 4 | -37835.50 | 0.008591188 | 0.01518529 | 2.289152 | 16.28032 | 0.02975225 |
|  | A | SI (1) | -20522.94 | 0.011497905 | 0.03928727 | -0.1717898 | 6.013830 | 0.09161355 |
|  |  | SI (2) | -25090.39 | 0.009155444 | 0.02804137 | 0.2335573 | 7.313749 | 0.09653951 |
|  |  | SI (3) | -26273.79 | 0.008882020 | 0.02912131 | 0.5756746 | 6.682379 | 0.11429963 |
|  |  | SI (4) | -27568.73 | 0.008719952 | 0.02448430 | 0.6990386 | 7.844036 | 0.08933342 |
|  |  | SI (5) | -27005.74 | 0.008118878 | 0.02471692 | 1.2186120 | 7.215250 | 0.07974893 |
| Equilibrium | A | SI | - 131155.4 | 0.0158811 | 0.03546742 | 0.4995445 | NA | NA |
|  |  | 1 | -65281.56 | 0.01507162 | 0.04313416 | 0.8159608 | NA | NA |
|  |  | 2 | -91454.60 | 0.01960859 | 0.05643326 | 1.2525782 | NA | NA |
|  |  | 3 | - 107172.02 | 0.02024149 | 0.05818902 | 1.3923090 | NA | NA |
|  |  | 4 | - 109316.83 | 0.01969346 | 0.06122869 | 1.5908392 | NA | NA |
|  |  | 5 | - 102871.58 | 0.01956371 | 0.06441036 | 1.7487296 | NA | NA |
|  |  | 6 | - 109530.37 | 0.02022063 | 0.06653580 | 1.8280224 | NA | NA |
|  |  | 7 | - 107081.11 | 0.02011955 | 0.06349083 | 1.8506064 | NA | NA |
|  |  | 8 | - 98821.91 | 0.01941300 | 0.06214699 | 1.9295693 | NA | NA |
|  |  | 9 | - 96782.19 | 0.01814207 | 0.06303110 | 2.0727241 | NA | NA |
|  |  | 10 | - 98156.17 | 0.01833303 | 0.06231082 | 2.1024864 | NA | NA |
|  |  | 11 | - 87204.98 | 0.01705616 | 0.06447494 | 2.2799683 | NA | NA |
|  |  | 12 | - 90449.59 | 0.01751604 | 0.06174893 | 2.2556753 | NA | NA |
|  |  | 13 | - 91078.33 | 0.01686392 | 0.06298159 | 2.3871022 | NA | NA |
|  |  | 14 | - 82468.48 | 0.01827334 | 0.06506738 | 2.4065044 | NA | NA |
|  |  | 15 | - 81674.91 | 0.01615289 | 0.06115533 | 2.5225456 | NA | NA |
|  |  | 16 | - 71123.21 | 0.01487083 | 0.06378156 | 2.7463899 | NA | NA |
|  |  | 17 | - 70500.77 | 0.01697558 | 0.06153363 | 2.6642449 | NA | NA |
|  |  | 18 | - 61216.56 | 0.01440179 | 0.05728001 | 2.8737783 | NA | NA |
|  |  | 19 | - 50974.47 | 0.01457243 | 0.05882804 | 3.0652331 | NA | NA |
|  |  | 20 | - 38309.66 | 0.01040679 | 0.05651837 | 3.7048632 | NA | NA |
|  | X | SI | - 11969 | 0.0158811 | 0.03546742 | 0.4995445 | NA | NA |
|  |  | 1 | - 65643.73 | 0.012196505 | 0.03118302 | 1.492375 | NA | NA |
|  |  | 2 | - 68346.39 | 0.013205564 | 0.03997431 | 2.165834 | NA | NA |
|  |  | 3 | - 56340.95 | 0.011880659 | 0.03826363 | 2.537929 | NA | NA |
|  |  | 4 | - 38610.28 | 0.009880136 | 0.03804166 | 3.152079 | NA | NA |
|  | A | SI (1) | - 20757.52 | 0.02329085 | 0.07280258 | - 0.2774824 | NA | NA |
|  |  | SI (2) | - 25428.77 | 0.02035433 | 0.06262571 | 0.2486126 | NA | NA |
|  |  | SI (3) | - 26603.07 | 0.01911206 | 0.06720719 | 0.6767407 | NA | NA |
|  |  | SI (4) | - 27950.52 | 0.01833015 | 0.05749812 | 0.8442205 | NA | NA |
|  |  | SI (5) | - 27326.58 | 0.01379074 | 0.05494312 | 1.5395580 | NA | NA |

^a^ Assuming no demographic change (the equilibrium model) or a single step-change in population size (the size change model).

^b^ Chromosome.

^c^ 4-fold degenerate sites were binned based on the GC-content of the *D. melanogaster* reference sequence. SI sites were investigated together (no parenthesis after ‘SI’), as well as binned (parentheses after ‘SI’) for the autosomal sites, also based on the GC-content of the *D. melanogaster* reference sequence.

^d^ The mutation rate from $AT\to GC$ (theta_10) and from $GC\to AT$ (theta_01).

^e^ Values of the magnitude of selection in favour of GC alleles ($\gamma=4N_{e}s$).

^f^ The factor by which the effective population size changes at time tau_1.

^g^ The time in generations into the past, scaled by twice the effective population size, at which the population size change takes place.

Table S5. Parameters from the ZC models for *D. melanogaster*

| Model^a^ | Chr^b^ | Bin^c^ | Ln-likelihood | theta_10^d^ | theta_01^d^ | Gamma^e^ | rho_1^f^ | tau_1^g^ |
| --- | --- | --- | --- | --- | --- | --- | --- | --- |
| Size Change | A | SI | -121492.4 | 0.005685958 | 0.01779346 | 0.5001906 | 1.553977 | 0.1540019 |
|  |  | 1 | -36182.52 | 0.006500024 | 0.01509887 | 0.6175125 | 500 | 3.399730e-05 |
|  |  | 2 | -47252.64 | 0.005963442 | 0.01615820 | 1.0826345 | 1.2147262 | 2.893665e-01 |
|  |  | 3 | -57014.23 | 0.007589809 | 0.01590732 | 0.9466280 | 500 | 3.169093e-05 |
|  |  | 4 | -58298.87 | 0.008509285 | 0.01996567 | 1.1621763 | 500 | 2.248592e-05 |
|  |  | 5 | -54897.05 | 0.008155369 | 0.01514481 | 0.9922920 | 500 | 6.015833e-05 |
|  |  | 6 | -52518.52 | 0.008284308 | 0.01645848 | 1.1348985 | 500 | 2.714025e-05 |
|  |  | 7 | -53317.92 | 0.007267020 | 0.01622980 | 1.3145151 | 500 | 3.506102e-05 |
|  |  | 8 | -49407.68 | 0.009336927 | 0.01856653 | 1.2631235 | 500 | 1.904870e-05 |
|  |  | 9 | -49487.35 | 0.009435474 | 0.01834430 | 1.3002389 | 500 | 1.735029e-05 |
|  |  | 10 | -47187.07 | 0.007966337 | 0.01820392 | 1.5194639 | 1.1839371 | 4.603757e-02 |
|  |  | 11 | -46458.31 | 0.009096294 | 0.01718026 | 1.3762971 | 1.0624492 | 1.036494e-01 |
|  |  | 12 | -43931.79 | 0.009921110 | 0.01826941 | 1.4088674 | 500 | 4.629618e-05 |
|  |  | 13 | -42191.62 | 0.015545353 | 0.03537482 | 1.7388536 | 0.5129453 | 3.002821e+00 |
|  |  | 14 | -40616.32 | 0.013938791 | 0.02385576 | 1.4662953 | 0.7265423 | 2.367621e+00 |
|  |  | 15 | -37796.98 | 0.009660859 | 0.01696978 | 1.5395049 | 500 | 2.767496e-05 |
|  |  | 16 | -32697.30 | 0.012079715 | 0.02545081 | 1.8260914 | 0.7265423 | 2.056413e+00 |
|  |  | 17 | -32943.16 | 0.009439723 | 0.01839872 | 1.8114047 | 500 | 2.636547e-05 |
|  |  | 18 | -31157.64 | 0.009670571 | 0.01820742 | 1.8830743 | 500 | 3.037606e-05 |
|  |  | 19 | -24692.54 | 0.010375310 | 0.01819292 | 1.9719160 | 500 | 4.511005e-05 |
|  |  | 20 | -16603.19 | 0.011530292 | 0.01762483 | 2.1742313 | 1.6769864 | 1.766517e-02 |
|  | X | SI | -12531.25 | 0.0061646 | 0.01709489 | 0.5810461 | 6.564036 | 0.02823154 |
|  |  | 1 | -35305.95 | 0.007643092 | 0.01786317 | 1.283422 | 4.073685 | 0.0295373581 |
|  |  | 2 | -36404.78 | 0.009655024 | 0.01874947 | 1.530942 | 4.576675 | 0.0295373581 |
|  |  | 3 | -29041.17 | 0.010719485 | 0.01748033 | 1.640160 | 500 | 0.0001474021 |
|  |  | 4 | -18290.01 | 0.012525851 | 0.01832996 | 1.960835 | 500 | 0.0001291119 |
|  | A | SI (1) | -17617.40 | 0.002504979 | 0.007427115 | -0.4401380 | 3.689975 | 1.497151e+00 |
|  |  | SI (2) | -22987.10 | 0.006413246 | 0.021397022 | 0.2213338 | 500 | 4.850392e-05 |
|  |  | SI (3) | -25441.95 | 0.005397022 | 0.021667719 | 0.7204925 | 5.383871 | 6.425344e-03 |
|  |  | SI (4) | -26797.50 | 0.004630329 | 0.014164251 | 0.7518270 | 1.585475 | 7.412774e-01 |
|  |  | SI (5) | -24385.35 | 0.004913514 | 0.014166080 | 1.1572279 | 2.182759 | 1.177823e-01 |
| Equilibrium | A | SI | -121516.8 | 0.006320698 | 0.02054881 | 0.5399996 | NA | NA |
|  |  | 1 | - 36184.38 | 0.006670162 | 0.01581766 | 0.6380540 | NA | NA |
|  |  | 2 | - 47254.45 | 0.006239309 | 0.01770309 | 1.1296884 | NA | NA |
|  |  | 3 | - 57017.07 | 0.007717821 | 0.01668366 | 0.9774092 | NA | NA |
|  |  | 4 | - 58300.42 | 0.008571192 | 0.02067149 | 1.1892328 | NA | NA |
|  |  | 5 | - 54907.80 | 0.008430991 | 0.01662497 | 1.0535752 | NA | NA |
|  |  | 6 | - 52520.52 | 0.008365851 | 0.01717267 | 1.1672576 | NA | NA |
|  |  | 7 | - 53321.41 | 0.007327567 | 0.01720628 | 1.3647215 | NA | NA |
|  |  | 8 | - 49408.62 | 0.009377884 | 0.01911945 | 1.2873147 | NA | NA |
|  |  | 9 | - 49488.09 | 0.009464635 | 0.01883930 | 1.3228482 | NA | NA |
|  |  | 10 | - 47187.34 | 0.007971587 | 0.01860015 | 1.5388375 | NA | NA |
|  |  | 11 | - 46458.41 | 0.009107968 | 0.01737581 | 1.3845659 | NA | NA |
|  |  | 12 | - 43938.18 | 0.010017913 | 0.01976989 | 1.4796540 | NA | NA |
|  |  | 13 | - 42193.56 | 0.009549935 | 0.01909058 | 1.5454150 | NA | NA |
|  |  | 14 | - 40616.86 | 0.011013301 | 0.01779957 | 1.3841752 | NA | NA |
|  |  | 15 | - 37798.79 | 0.009670581 | 0.01779472 | 1.5857796 | NA | NA |
|  |  | 16 | - 32697.79 | 0.010286432 | 0.01993230 | 1.7169357 | NA | NA |
|  |  | 17 | - 32944.70 | 0.009381774 | 0.01929715 | 1.8650248 | NA | NA |
|  |  | 18 | - 31159.70 | 0.009583366 | 0.01926207 | 1.9487349 | NA | NA |
|  |  | 19 | - 24696.54 | 0.010208655 | 0.01983004 | 2.0766358 | NA | NA |
|  |  | 20 | - 16603.71 | 0.011381331 | 0.01837576 | 2.2283784 | NA | NA |
|  | X | SI | - 12560.42 | 0.007834216 | 0.02414876 | 0.6934692 | NA | NA |
|  |  | 1 | - 35342.90 | 0.008208799 | 0.02291368 | 1.469861 | NA | NA |
|  |  | 2 | - 36448.20 | 0.010021248 | 0.02409939 | 1.756472 | NA | NA |
|  |  | 3 | - 29083.13 | 0.010864753 | 0.02241613 | 1.887741 | NA | NA |
|  |  | 4 | - 18314.78 | 0.012152670 | 0.02315406 | 2.239197 | NA | NA |
|  | A | SI (1) | - 17620.08 | 0.007920455 | 0.02337138 | - 0.4715533 | NA | NA |
|  |  | SI (2) | - 22989.94 | 0.006737756 | 0.02270366 | 0.2315056 | NA | NA |
|  |  | SI (3) | - 25445.60 | 0.005623560 | 0.02344516 | 0.7591773 | NA | NA |
|  |  | SI (4) | - 26800.90 | 0.006067517 | 0.01957775 | 0.8109537 | NA | NA |
|  |  | SI (5) | - 24400.82 | 0.005523445 | 0.01865774 | 1.3220496 | NA | NA |

^a^ Assuming no demographic change (the equilibrium model) or a single step-change in population size (the size change model).

^b^ Chromosome.

^c^ 4-fold degenerate sites were binned based on the GC-content of the *D. melanogaster* reference sequence. SI sites were investigated together (no parenthesis after ‘SI’), as well as binned (parentheses after ‘SI’) for the autosomal sites, also based on the GC-content of the *D. melanogaster* reference sequence.

^d^ The mutation rate from $AT\to GC$ (theta_10) and from $GC\to AT$ (theta_01).

^e^ Values of the magnitude of selection in favour of GC alleles ($\gamma=4N_{e}s$).

^f^ The factor by which the effective population size changes at time tau_1.

^g^ The time in generations into the past, scaled by twice the effective population size, at which the population size change takes place.

Table S6. Parameters from the ZC models for *D. melanogaster* autosomal sites grouped into 10 bins, and for different ranges of the possible value that rho_1 may take.

| Model^a^ | Chr^b^ | Bin^c^ | Max_rho_1^d^ | Ln-likelihood | theta_10^e^ | theta_01^e^ | Gamma^f^ | rho_1^g^ | tau_1^h^ |
| --- | --- | --- | --- | --- | --- | --- | --- | --- | --- |
| Size Change | A | 1 | 500 | -83696.85 | 0.006202299 | 0.01570633 | 0.8813971 | 1.3601229 | 1.095472e-01 |
|  |  | 1 | 1000 | -83696.85 | 0.006202298 | 0.01570633 | 0.8813968 | 1.360123 | 1.095477e-01 |
|  |  | 2 | 500 | -115366.89 | 0.008045821 | 0.01790669 | 1.0604380 | 500 | 3.363704e-05 |
|  |  | 2 | 1000 | -115366.88 | 0.008045815 | 0.01790660 | 1.0604336 | 1000 | 1.680200e-05 |
|  |  | 3 | 500 | -107435.51 | 0.008233529 | 0.01571558 | 1.0591412 | 500 | 5.209305e-05 |
|  |  | 3 | 1000 | -107435.50 | 0.008233555 | 0.01571565 | 1.0591425 | 1000 | 2.601613e-05 |
|  |  | 4 | 500 | -102754.14 | 0.008238216 | 0.01730981 | 1.2875430 | 500 | 3.385069e-05 |
|  |  | 4 | 1000 | -102754.14 | 0.008238220 | 0.01730975 | 1.2875394 | 1000 | 1.690793e-05 |
|  |  | 5 | 500 | -96687.59 | 0.008742213 | 0.01825230 | 1.4037611 | 500 | 2.134020e-05 |
|  |  | 5 | 1000 | -96687.59 | 0.008742220 | 0.01825233 | 1.4037621 | 1000 | 1.065840e-05 |
|  |  | 6 | 500 | -90409.43 | 0.009516117 | 0.01772249 | 1.3939046 | 500 | 3.280051e-05 |
|  |  | 6 | 1000 | -90409.43 | 0.009516133 | 0.01772257 | 1.3939076 | 1000 | 1.638131e-05 |
|  |  | 7 | 500 | -82819.92 | 0.010953843 | 0.02095530 | 1.5407664 | 0.8649999 | 1.000000e+00 |
|  |  | 7 | 1000 | -82820.36 | 0.010294248 | 0.01834739 | 1.4619688 | 1000 | 5.284546e-06 |
|  |  | 8 | 500 | -70510.73 | 0.009986531 | 0.01820366 | 1.6181511 | 500 | 2.426038e-05 |
|  |  | 8 | 1000 | -70510.73 | 0.009986533 | 0.01820361 | 1.6181482 | 1000 | 1.211804e-05 |
|  |  | 9 | 500 | -64120.79 | 0.009579602 | 0.01823204 | 1.8441493 | 500 | 3.592394e-05 |
|  |  | 9 | 1000 | -64120.79 | 0.009579614 | 0.01823206 | 1.8441492 | 1000 | 1.794217e-05 |
|  |  | 10 | 500 | -41409.54 | 0.010846455 | 0.01785955 | 2.0517634 | 500 | 4.264177e-05 |
|  |  | 10 | 1000 | -41409.54 | 0.010846458 | 0.01785970 | 2.0517724 | 1000 | 2.129505e-05 |
| Equilibrium |  | 1 | NA | -83700.33 | 0.006415187 | 0.01689386 | 0.9184764 | NA | NA |
|  |  | 2 | NA | -115370.63 | 0.008124107 | 0.01869052 | 1.0904525 | NA | NA |
|  |  | 3 | NA | -107446.35 | 0.008398039 | 0.01689351 | 1.1093637 | NA | NA |
|  |  | 4 | NA | -102757.73 | 0.008275104 | 0.01812105 | 1.3250544 | NA | NA |
|  |  | 5 | NA | -96688.46 | 0.008729682 | 0.01870955 | 1.4248790 | NA | NA |
|  |  | 6 | NA | -90412.79 | 0.009535105 | 0.01853720 | 1.4327974 | NA | NA |
|  |  | 7 | NA | -82820.23 | 0.010251355 | 0.01844270 | 1.4651767 | NA | NA |
|  |  | 8 | NA | -70511.75 | 0.009943271 | 0.01878699 | 1.6486060 | NA | NA |
|  |  | 9 | NA | -64124.19 | 0.009475422 | 0.01927814 | 1.9060218 | NA | NA |
|  |  | 10 | NA | -41413.47 | 0.010629366 | 0.01917599 | 2.1388384 | NA | NA |

^a^ Assuming no demographic change (the equilibrium model) or a single step-change in population size (the size change model).

^b^ Chromosome.

^c^ 4-fold degenerate sites were binned based on the GC-content of the *D. melanogaster* reference sequence.

^d^ The maximum value that rho_1 is constrained to during the search.

^e^ The mutation rate from $AT\to GC$ (theta_10) and from $GC\to AT$ (theta_01).

^f^ Values of the magnitude of selection in favour of GC alleles ($\gamma=4N_{e}s$).

^g^ The factor by which the effective population size changes at time tau_1.

^h^ The time in generations into the past, scaled by twice the effective population size, at which the population size change takes place.
