# Supplementary material for: Variation in the Intensity of Selection on Codon Bias over Time Causes Contrasting Patterns of Base Composition Evolution in Drosophila
Source: Genome Biol Evol. 2017 Jan 12;9(1):102–23. doi: 10.1093/gbe/evw291 (PMC5381600; doi:10.1093/gbe/evw291)
Supplement: Supplementary Data [file evw291_Supp.zip › Dsim_supplement_v11.docx]

# Supplementary Material

Variation in the intensity of selection on codon bias over time causes contrasting patterns of base composition evolution in *Drosophila*

Benjamin C Jackson, José L Campos, Penelope R Haddrill, Brian Charlesworth and Kai Zeng

# Text S1

## The difference between the slopes for $\boldsymbol{r}_{\boldsymbol{S}\boldsymbol{\to}\boldsymbol{W}}$ and $\boldsymbol{r}_{\boldsymbol{W}\boldsymbol{\to}\boldsymbol{S}}$

We use the same model and notation as those that lead to Eq. (2) in the main text. As a rough approximation, it suffices to ask whether the ratio of the values of $r_{S\to W}$ observed in the bins with the lowest and highest GC content at 4-fold sites is larger than the ratio for $r_{W\to S}$ between the highest and lowest GC content bins, given the estimated values of $\gamma$ for the two bins. We consider only the autosomal data. In *D. simulans*, $\gamma$ was estimated to be 0.25 and 1.24, respectively, for the two extreme GC content bins (see the paragraph below Eq. (3)). The ratio of $r_{S\to W}$between the two GC content bins is given by the ratio of the values of $\gamma/\left[ exp\left( \gamma\right)-1 \right]$ (assuming similar $\kappa$ values for the two bins) whereas the ratio for $r_{W\to S}$ is the ratio of the values of $\gamma/\left[ 1-exp\left( -\gamma\right) \right]$. Substituting the two estimates of $\gamma into these expressions$, we find that, the ratio for $r_{S\to W}$ is 1.74 and is 1.55 for $r_{W\to S}$. The observed values are 2.13 and 1.27, respectively, which differ in the predicted direction.

In *D. melanogaster*, assuming that SI sites are neutrally evolving, then $\kappa$ can be estimated from the ratio of $r_{S\to W}$ to $r_{W\to S}$, which is 2.61. This is somewhat higher than the value of 2, which has been frequently quoted in the literature, but within the range of variation observed in mutation accumulation experiments (Schrider et al. 2013). Using the same approach as above, we first used Eq. (2) in the main text to estimate$\gamma$ for the two extreme GC bins on the autosomes, which are 0.02 and 1.01, respectively. These values predict that the ratios of $r_{S\to W}$ and $r_{W\to S}$ between the two extreme GC content bins are 1.71 and 1.57, respectively. The observed values are 1.93 and 1.40, respectively.

# Text S2

**Estimating demographic parameters using the Zeng and Charlesworth (ZC) method**

As shown in Supplementary Table S4, and consistent with the negative Tajima’s *D* values calculated using 4-fold sites from *D. simulans* (-1.03; Table 1), we detected clear evidence for a recent population expansion (*p* < 10^-16^ for all bins; Table S4). Since the ZC method and the Glémin method returned very similar *γ* estimates, despite the fact that they incorporate the effects of demography using very different approaches (see Materials and Methods), this suggests that the results obtained from these methods are unlikely to be artefacts.

The situation is somewhat more complex in *D. melanogaster*. Take the 20 autosomal 4-fold site bins as an example (Supplementary Table S5). The equilibrium model with a constant population size appears to be sufficient to explain the data in 16 of them, consistent with the fact that Tajima’s *D* is close to zero (-0.11; Table 1 in Jackson et al. 2015) and our previous suggestion that the mildly negative value is probably due to weak selection on CUB (Zeng and Charlesworth 2009).

As shown in Table S5, when the ZC1 model with a one-step change in population size was fitted to the autosomal bins in *D. melanogaster*, the algorithm sometimes converged to biologically unrealistic situations with a very large increase in population size (a 500-fold increase, which is the upper limit used to run the search) that took place a very short time ago (in the order of 10^-5^ x 2*N_c_* generations ago, where *N_c_* is the current effective population size). To investigate whether the above is due to the issue of statistical power, we also tried to reanalyse the data by reducing the number of bins to 10, and observed the same behaviour (Table S6). When increasing the upper limit to 1000, the algorithm converged to a model with a 1000-fold increase in size but an even shorter time since the expansion, and these estimates have virtually identical ln-likelihood as those found in the analyses with the upper limit set to 500 (Supplementary Table S6).

Compared to the 20-bin analysis, in seven of the 10 bins, the likelihood ratio test for comparing the equilibrium model and the model with a one-step change in population size now has a *p*-value less than 0.05. However, except for one bin where *p* = 1.96e-05, the *p*-values in the other bins are only marginally significant (ranging between 1.92% and 3.47%). According to our previous simulation study (Zeng and Charlesworth 2010), as well as results from other workers (e.g., Messer and Petrov 2013; Sousa et al. 2013), these results are more likely to be caused by violation of model assumptions and/or minor numerical inaccuracy induced by the limited precision of computer floating point arithmetic. In fact, both Zeng and Charlesworth (2010) and Sousa et al. (2013) observed that giving the chi-squared test one or two extra degrees of freedom can often resolve these artefacts. When testing the results using chi-squared tests with three or four degrees of freedom, respectively, two and one bin from the 10-bin analysis and three and two bins from the 20-bin analysis remain significant, close to the expected number of significant tests by chance under multiple testing. In contrast, all results from the 20 bins in *D. simulans* remain highly significant regardless of the degrees of freedom used (max(*p*) < 10^-16^).

Putting all this together, we interpret the results in *D. melanogaster* as lack of clear support for a recent expansion. This conclusion is in line with the mildly negative Tajima’s *D* (-0.11). More importantly, even if there were extremely recent explosive population growth, it is unlikely that the signal can be picked up by a sample of 17 alleles (studies in humans have shown that thousands or even tens of thousands of alleles are necessary), and therefore our conclusions are unlikely to be affected. In fact, as shown in Supplementary Figure S13, *γ* estimates produced by either the equilibrium and non-equilibrium versions of the ZC method, in either the analysis based on 10 or 20 bins, are very similar, both being higher than those estimated by the Glémin method.

# Tables

**Table S1.** **Summary statistics for the unfiltered *D. simulans* dataset**

| Chr^a^ | Site |  | Within- population statistics | | | | |  | Population  differentiation |
| --- | --- | --- | --- | --- | --- | --- | --- | --- | --- |
|  |  |  | Pop.^b^ | *π*^c^ | *θ_W_*^d^ | *Δ_π_*^e^ | *D*^f^ |  | *F_ST_* |
| A | 0-fold^g^ |  | MD | 0.00173 | 0.00293 | -0.121 | -1.33 |  | 0.0201 |
|  |  |  | NS | 0.00168 | 0.00239 | -0.0944 | -0.99 |  |  |
|  | 4-fold^h^ |  | MD | 0.0329 | 0.0457 | -0.0812 | -1.07 |  | 0.0258 |
|  |  |  | NS | 0.0314 | 0.038 | -0.0517 | -0.666 |  |  |
| X | 0-fold |  | MD | 0.00128 | 0.00222 | -0.124 | -1.28 |  | 0.0169 |
|  |  |  | NS | 0.00126 | 0.00184 | -0.0973 | -0.974 |  |  |
|  | 4-fold |  | MD | 0.0191 | 0.0296 | -0.104 | -1.32 |  | 0.0242 |
|  |  |  | NS | 0.0182 | 0.024 | -0.0731 | -0.89 |  |  |

All statistics were calculated per gene, and the means are presented here.

^a^ Chromosome

^b^ Population sample: MD – Madagascar; NS – Kenya

^c^ Average number of pairwise differences between lines

^d^ Watterson’s estimator of *θ*, the scaled mutation rate

^e^ See equation (1)

^f^ Tajima’s *D*

^g^ 0-fold degenerate sites

^h^ 4-fold degenerate sites

**Table S2.** **A comparison between substitutions along the *D. melanogaster* and *D. simulans* lineages.** Inferred from the dataset presented in this study, and from an independent dataset provided by Juraj Bergman and Claus Vogl (pers. comm.). Substutitions were inferred using parsimony at 4-fold degenerate and SI sites.

|  |  |  | *D. simulans* | | | | | | |  | *D. melanogaster* | | | | | | |
| --- | --- | --- | --- | --- | --- | --- | --- | --- | --- | --- | --- | --- | --- | --- | --- | --- | --- |
|  |  |  | A | | |  | X | | |  | A | | |  | X | | |
| Site | Dataset |  | $AT\to GC$ |  | $GC\to AT$ |  | $AT\to GC$ |  | $GC\to AT$ |  | $AT\to GC$ |  | $GC\to AT$ |  | $AT\to GC$ |  | $GC\to AT$ |
| 4-fold | This study |  | 13607 |  | 25656 |  | 1962 |  | 3934 |  | 10588 |  | 40586 |  | 1140 |  | 7395 |
|  | Bergman & Vogl |  | 8178 |  | 13917 |  | 1581 |  | 3789 |  | 10142 |  | 41858 |  | 1219 |  | 8587 |
| SI | This study |  | 1859 |  | 1598 |  | 206 |  | 152 |  | 1570 |  | 1884 |  | 131 |  | 229 |
|  | Bergman & Vogl |  | 1650 |  | 1345 |  | 196 |  | 206 |  | 2592 |  | 2996 |  | 226 |  | 352 |

**Table S3.** **Glémin et al. (2015) model AIC rankings.** Model M0 - $\gamma=0$, without polarisation error; Model M1 - $\gamma\neq0$, without polarisation error; Model M0* - $\gamma=0$, with polarisation error; Model M1* - $\gamma\neq0$, with polarisation error.

|  |  |  |  |  | AIC model ranking (1 is the best) | | | |
| --- | --- | --- | --- | --- | --- | --- | --- | --- |
| Species | Chr. | Site | Bin |  | M0 | M0* | M1 | M1* |
| *simulans* | A | SI |  |  | 3 | 4 | 1 | 2 |
|  |  | 4-fold | 1 |  | 4 | 3 | 1 | 2 |
|  |  |  | 2 |  | 4 | 3 | 1 | 2 |
|  |  |  | 3 |  | 4 | 3 | 1 | 2 |
|  |  |  | 4 |  | 4 | 3 | 2 | 1 |
|  |  |  | 5 |  | 4 | 3 | 1 | 2 |
|  |  |  | 6 |  | 4 | 3 | 2 | 1 |
|  |  |  | 7 |  | 4 | 3 | 1 | 2 |
|  |  |  | 8 |  | 4 | 3 | 2 | 1 |
|  |  |  | 9 |  | 4 | 3 | 1 | 2 |
|  |  |  | 10 |  | 4 | 3 | 2 | 1 |
|  |  |  | 11 |  | 4 | 3 | 2 | 1 |
|  |  |  | 12 |  | 4 | 3 | 1 | 2 |
|  |  |  | 13 |  | 4 | 3 | 2 | 1 |
|  |  |  | 14 |  | 4 | 3 | 2 | 1 |
|  |  |  | 15 |  | 4 | 3 | 2 | 1 |
|  |  |  | 16 |  | 4 | 3 | 1 | 2 |
|  |  |  | 17 |  | 4 | 3 | 2 | 1 |
|  |  |  | 18 |  | 4 | 3 | 1 | 2 |
|  |  |  | 19 |  | 4 | 3 | 2 | 1 |
|  |  |  | 20 |  | 4 | 3 | 2 | 1 |
|  | X | SI |  |  | 1 | 3 | 2 | 4 |
|  |  | 4-fold | 1 |  | 4 | 3 | 1 | 2 |
|  |  |  | 2 |  | 4 | 3 | 2 | 1 |
|  |  |  | 3 |  | 4 | 3 | 2 | 1 |
|  |  |  | 4 |  | 4 | 3 | 2 | 1 |
| *melanogaster* | A | SI |  |  | 4 | 3 | 1 | 2 |
|  |  | 4-fold | 1 |  | 1 | 3 | 2 | 4 |
|  |  |  | 2 |  | 4 | 3 | 1 | 2 |
|  |  |  | 3 |  | 1 | 3 | 2 | 4 |
|  |  |  | 4 |  | 2 | 4 | 1 | 3 |
|  |  |  | 5 |  | 1 | 3 | 2 | 4 |
|  |  |  | 6 |  | 1 | 3 | 2 | 4 |
|  |  |  | 7 |  | 4 | 2 | 1 | 3 |
|  |  |  | 8 |  | 1 | 3 | 2 | 4 |
|  |  |  | 9 |  | 2 | 3 | 1 | 4 |
|  |  |  | 10 |  | 4 | 2 | 1 | 3 |
|  |  |  | 11 |  | 4 | 2 | 1 | 3 |
|  |  |  | 12 |  | 2 | 3 | 1 | 4 |
|  |  |  | 13 |  | 3 | 4 | 1 | 2 |
|  |  |  | 14 |  | 1 | 3 | 2 | 4 |
|  |  |  | 15 |  | 1 | 3 | 2 | 4 |
|  |  |  | 16 |  | 2 | 3 | 1 | 4 |
|  |  |  | 17 |  | 4 | 2 | 1 | 3 |
|  |  |  | 18 |  | 4 | 2 | 1 | 3 |
|  |  |  | 19 |  | 4 | 3 | 1 | 2 |
|  |  |  | 20 |  | 4 | 3 | 1 | 2 |
|  | X | SI |  |  | 2 | 4 | 1 | 3 |
|  |  | 4-fold | 1 |  | 2 | 4 | 1 | 3 |
|  |  |  | 2 |  | 1 | 3 | 2 | 4 |
|  |  |  | 3 |  | 1 | 3 | 2 | 4 |
|  |  |  | 4 |  | 1 | 3 | 2 | 4 |
|  | A | 4-fold | 1&2 |  | 3 | 2 | 1 | 4 |
|  |  |  | 3&4 |  | 3 | 4 | 1 | 2 |
|  |  |  | 5&6 |  | 1 | 3 | 2 | 4 |
|  |  |  | 7&8 |  | 4 | 2 | 1 | 3 |
|  |  |  | 9&10 |  | 3 | 4 | 1 | 2 |
|  |  |  | 11&12 |  | 4 | 2 | 1 | 3 |
|  |  |  | 13&14 |  | 2 | 4 | 1 | 3 |
|  |  |  | 15&16 |  | 2 | 4 | 1 | 3 |
|  |  |  | 17&18 |  | 4 | 3 | 1 | 2 |
|  |  |  | 19&20 |  | 4 | 3 | 1 | 2 |

# Figures

**Figure S1.** **Residual heterozygosity per *D. simulans* isofemale line.** Calculated as the proportion of sites that were called as heterozygotes in the entire genome. Orange columns – the unfiltered dataset; blue columns – the 95% VQSR filtered dataset (see Material and Methods).

| 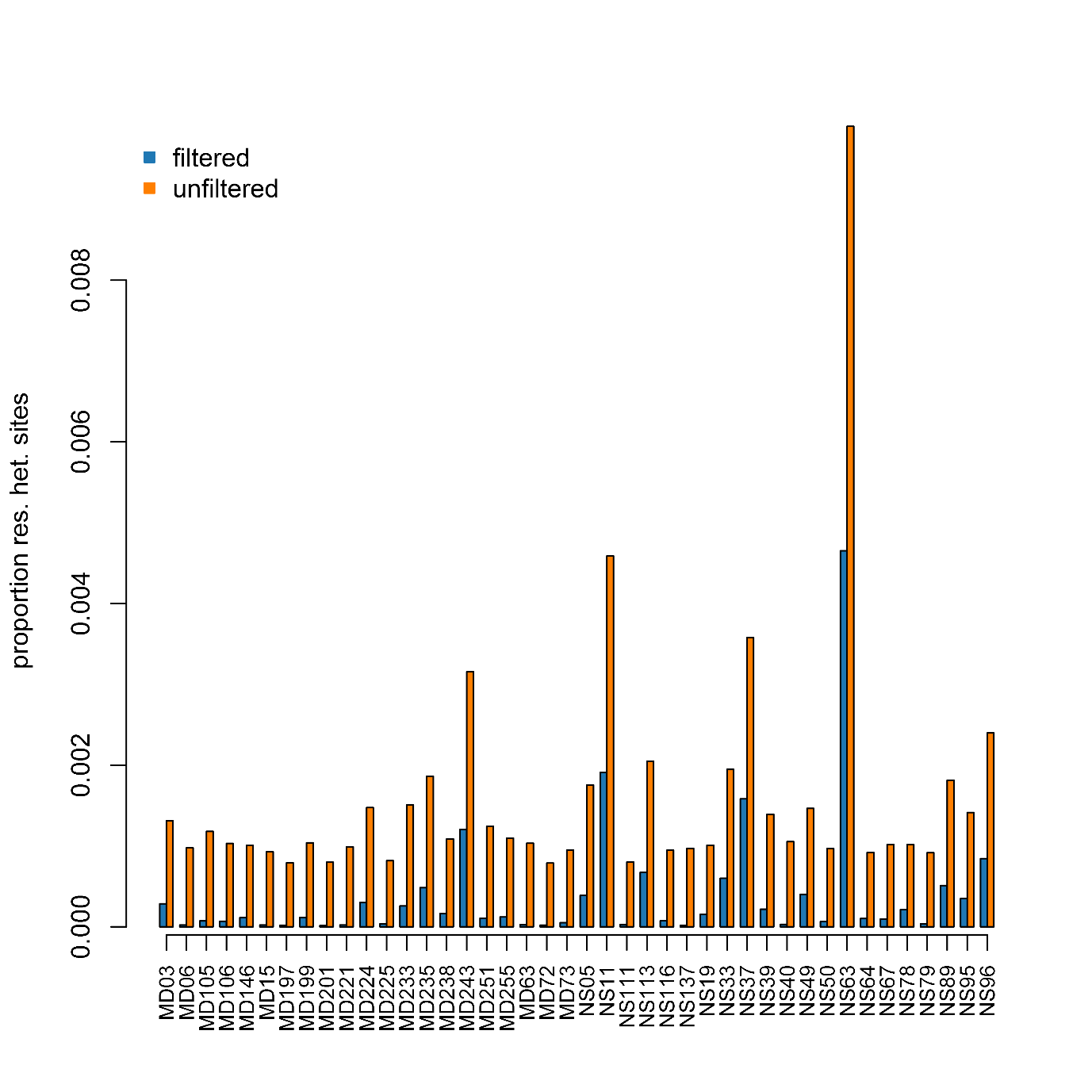 |
| --- |

**Figure S2.** **Comparison between estimates of the magnitude of selection for GC alleles (**$\boldsymbol{\gamma=4}\boldsymbol{N}_{\boldsymbol{e}}\boldsymbol{s}$**) from the method of Glémin et al. (2015)**. Panel A – autosomal sites from the Madagascan (MD) *D. simulans* sample; panel B – X chromosome sites from the MD *D. simulans* sample; panel C – autosomal sites from the Rwandan (RG) *D. melanogaster* sample; panel D – X chromosome sites from the RG *D. melanogaster* sample. Pink squares – model M1; grey triangles – model M1*. Filled points – bins where a model with $\gamma\neq0$ fitted best; open points – bins where a model with $\gamma=0$ fitted best.

| 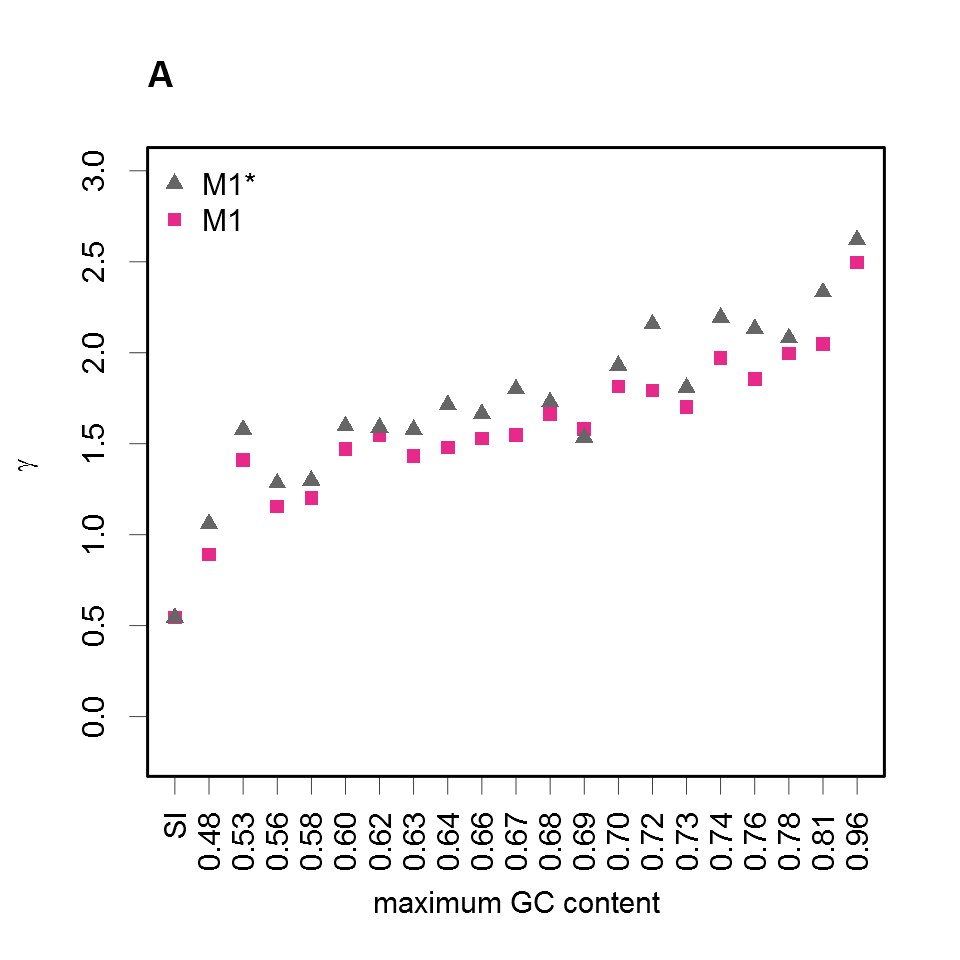 | 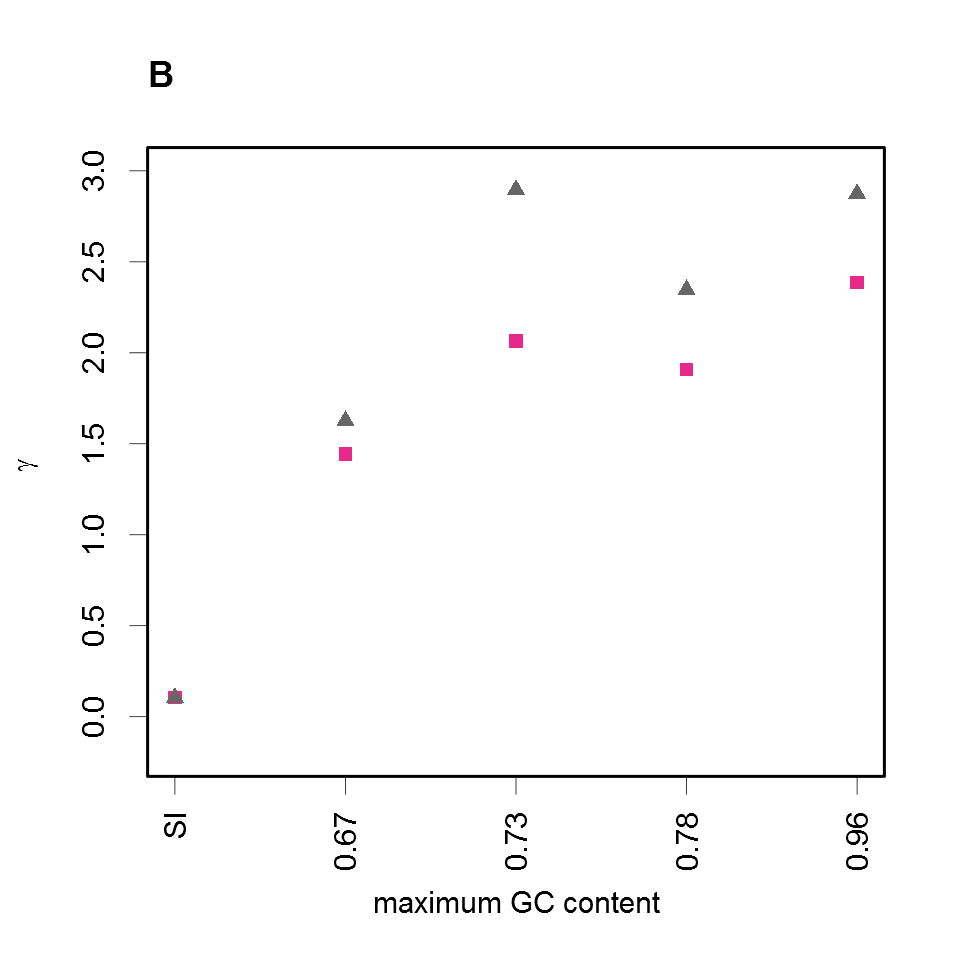 |
| --- | --- |
| 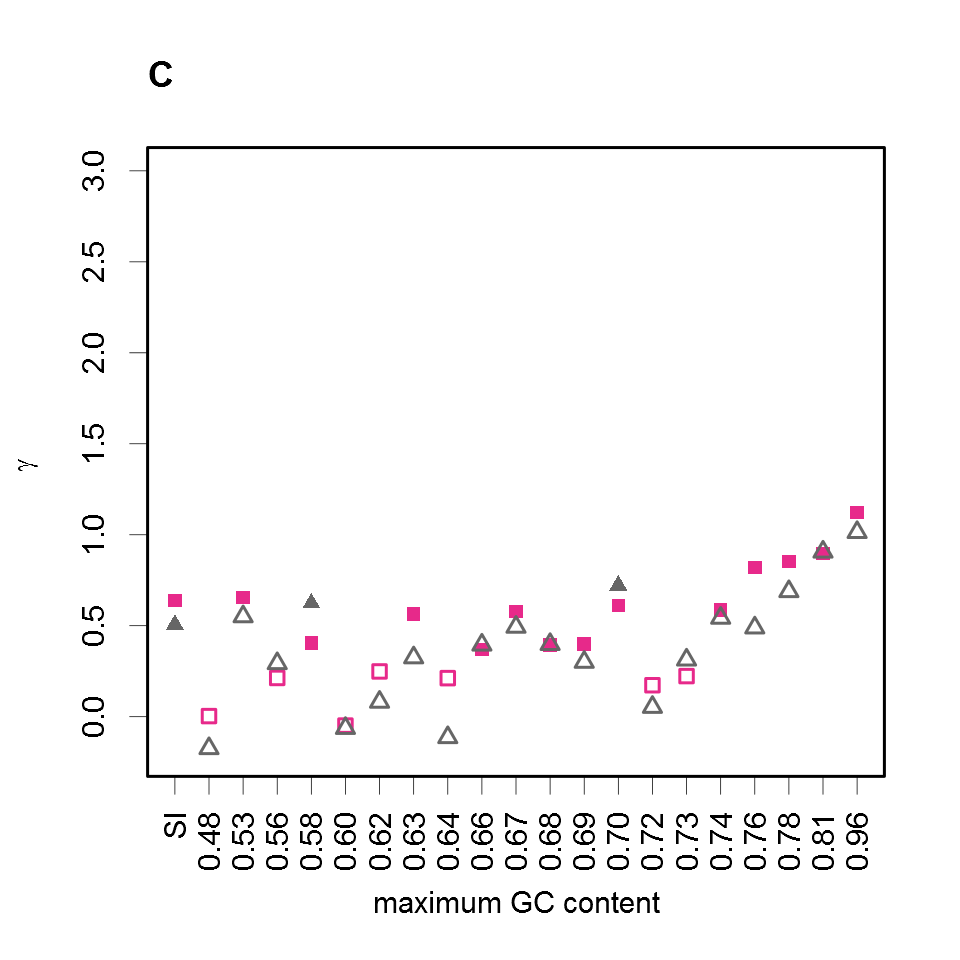 | 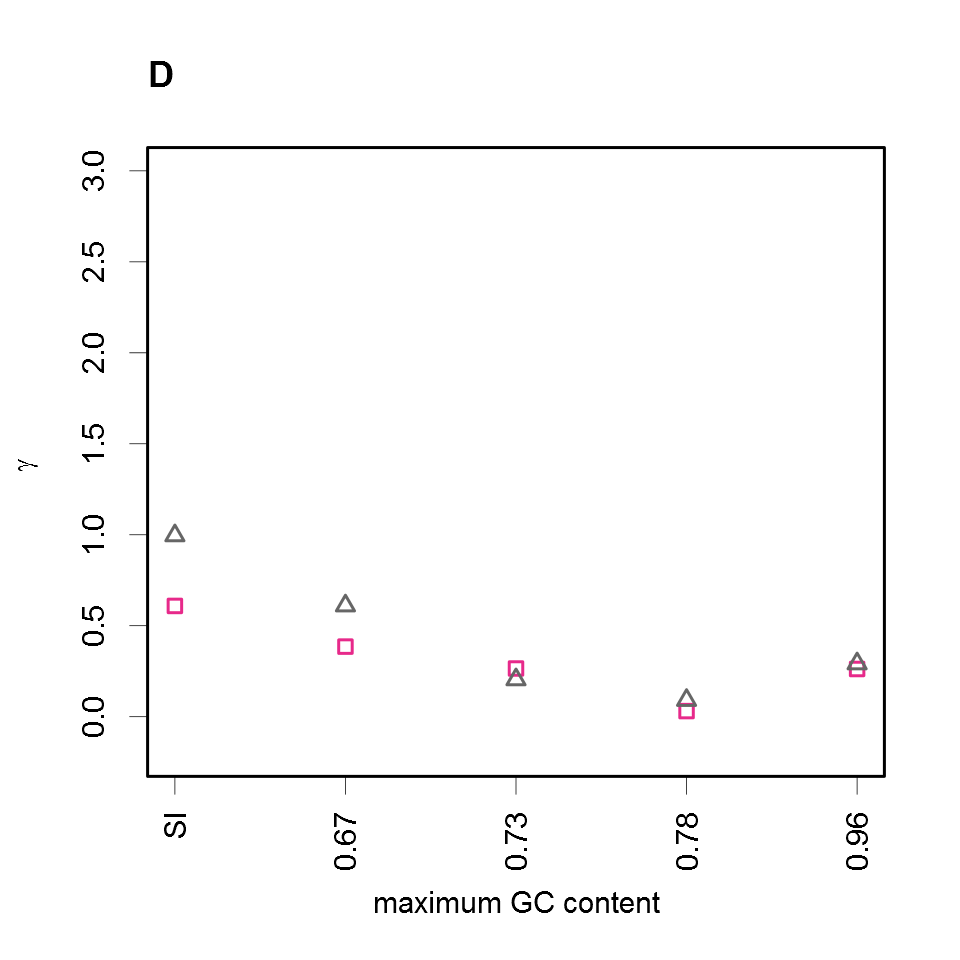 |

**Figure S3.** **Estimates of values of the force favouring GC alleles (**$\boldsymbol{\gamma=4}\boldsymbol{N}_{\boldsymbol{e}}\boldsymbol{s}$**).** For Rwandan (RG) *D. melanogaster* autosomal 4-fold degenerate sites, reduced to 10 GC content bins. $\gamma$ was calculated using the method of Glémin et al. (2015), incorporating polarisation errors (M1* in the main text) – grey triangles; and not incorporating polarisation errors (M1 in the main text) – pink squares. Filled points – bins where a model with $\gamma\neq0$ fitted best; open points – bins where a model with $\gamma=0$ fitted best.


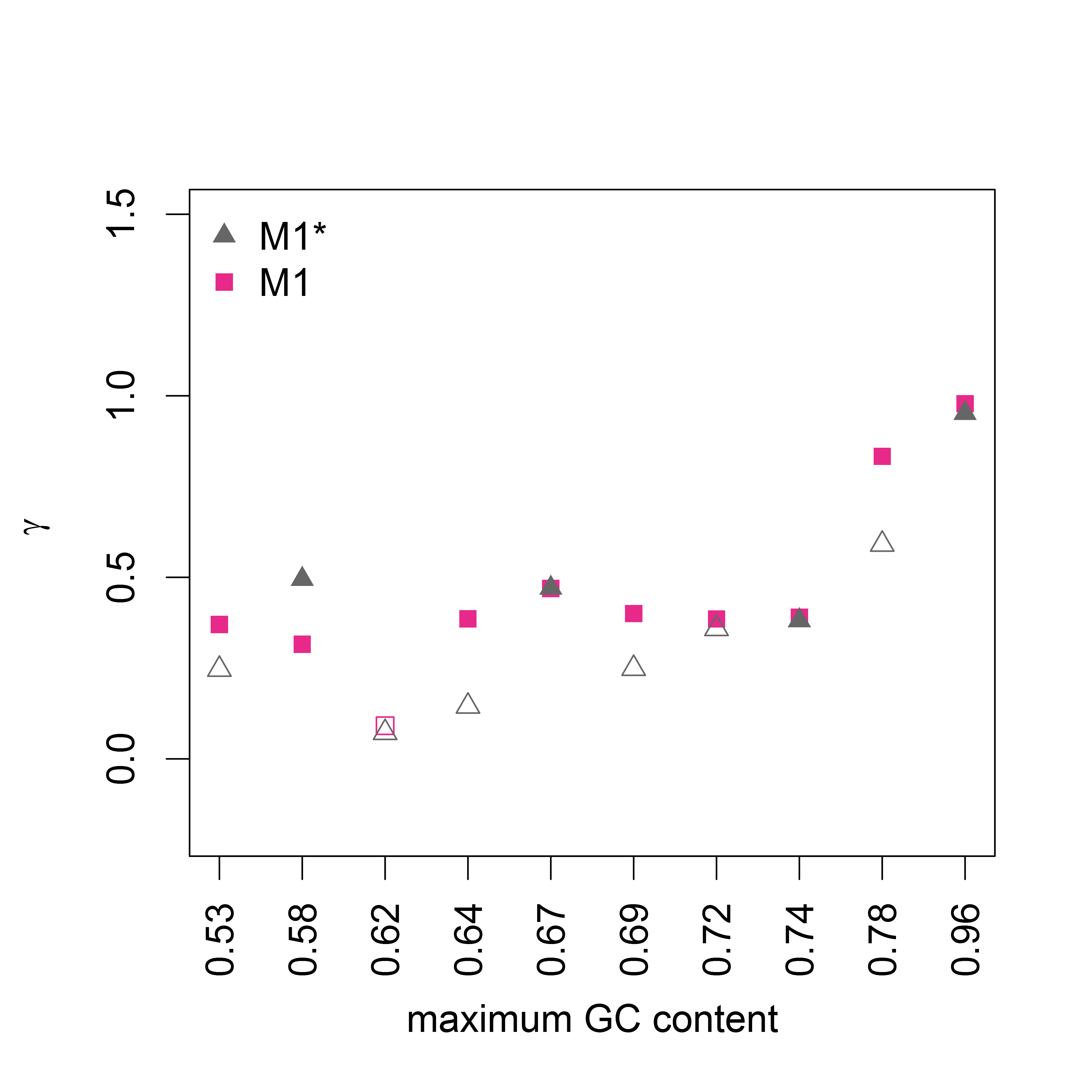


**Figure S4. The *Δ_π_* statistic of Langley et al (2014).** Panel A – autosomal sites from the Madagascan (MD) *D. simulans* sample; panel B – X-chromosome sites from the MD *D. simulans* sample; Panel C – autosomal sites from the Rwandan (RG) *D. melanogaster* sample; panel D – X chromosome sites from the RG *D. melanogaster* sample. In *D. simulans*, *Δ_π_* is negatively correlated with GC content at autosomal sites (Kendall’s τ = -0.88, p < 0.001; panel A). In *D. melanogaster*, *Δ_π_* is also negatively correlated with GC content at autosomal sites (Kendall’s τ = -0.41, p = 0.012; panel C).

| 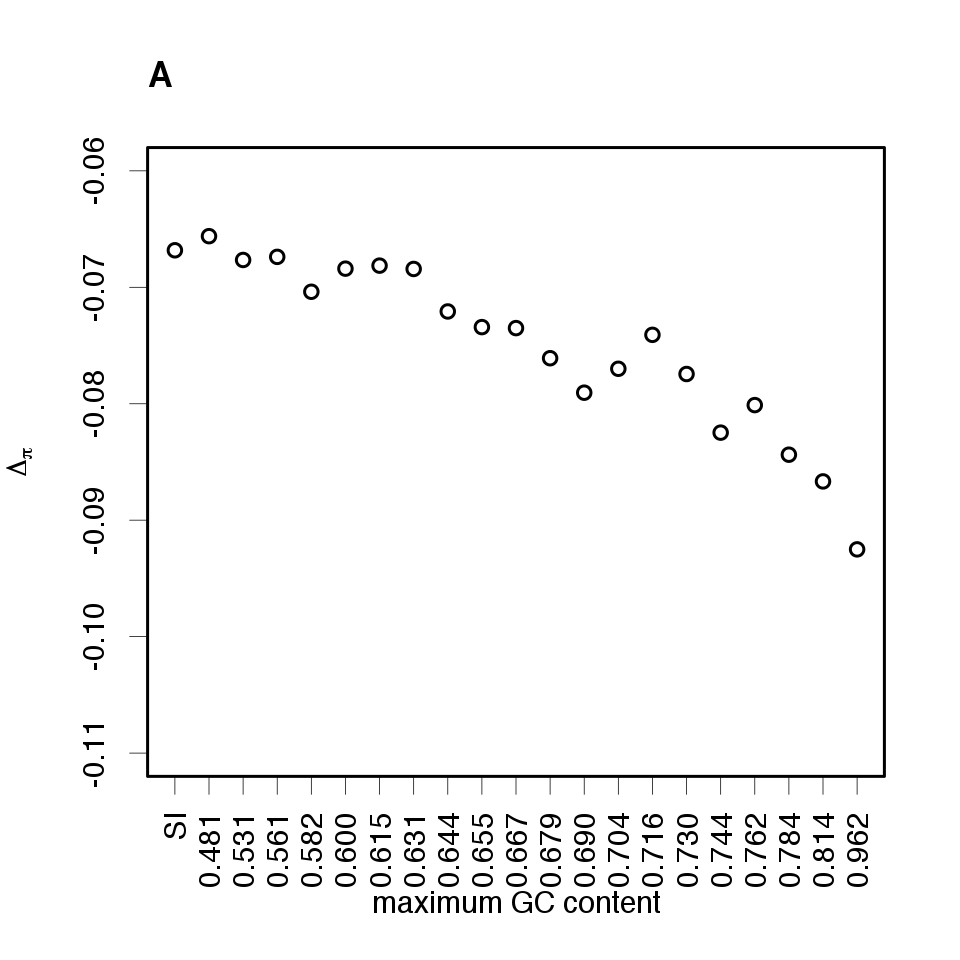 | 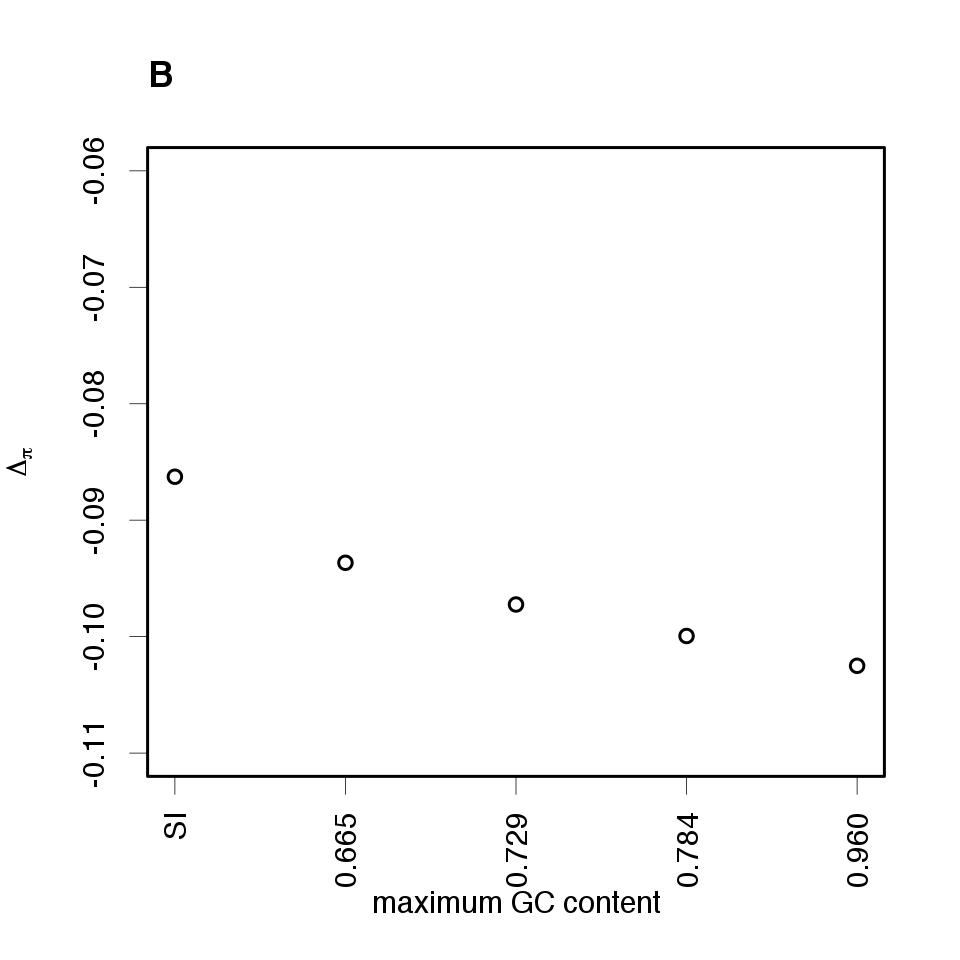 |
| --- | --- |
| 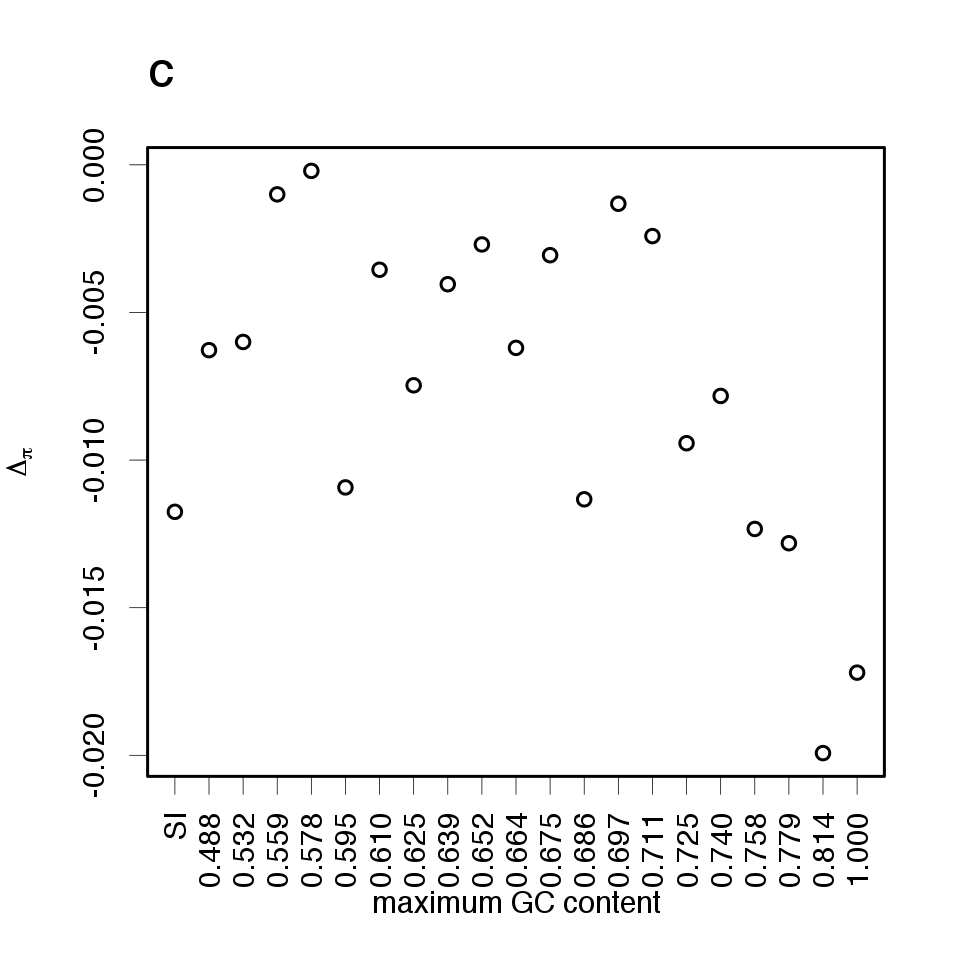 | 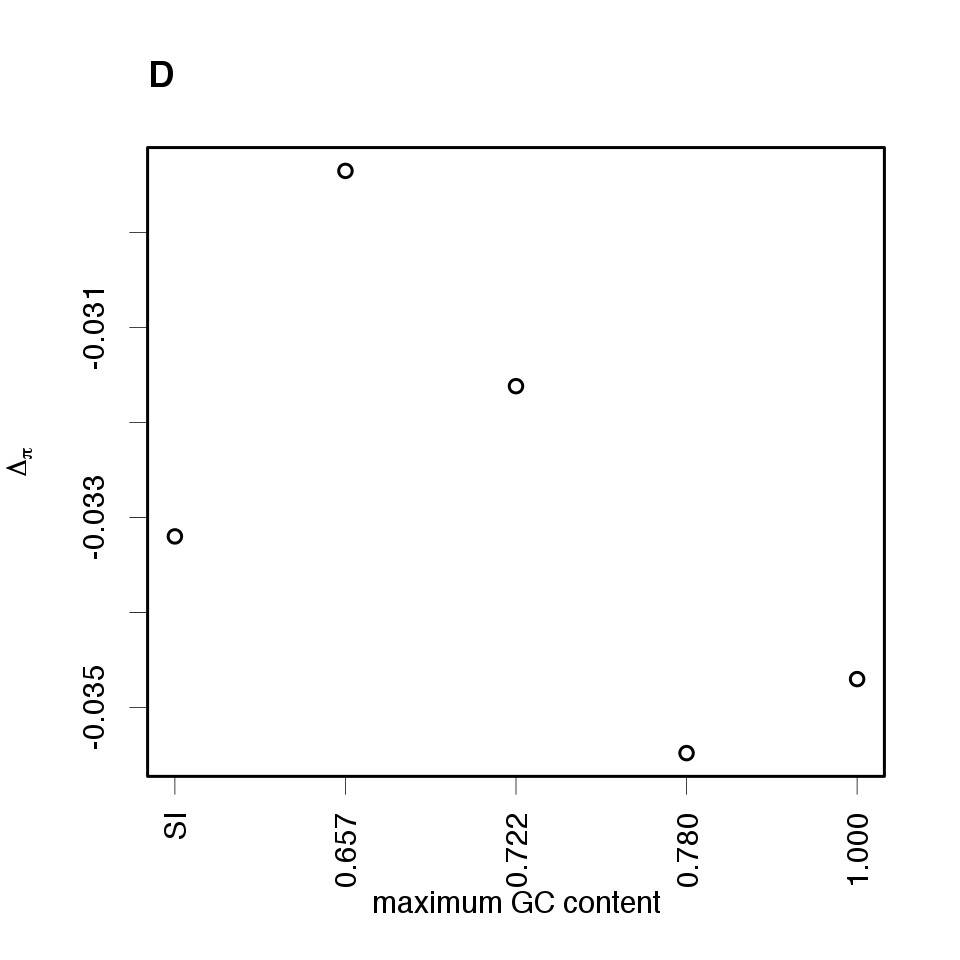 |

**Figure S5.** **The ratio of substitution rates.** For positions 8-30bp of introns <66bp long (SI sites; leftmost points), and 4-fold degenerate sites (remaining points) binned by the GC content of the extant *D. melanogaster* reference sequence, and using AWP to infer ancestral states. Panel A – autosomal substitution rates ratios along the *D. simulans* lineage; panel B – X chromosome rate ratios along the *D. simulans* lineage; panel C – autosomal substitution rate ratios along the *D. melanogaster* lineage; panel D – X chromosome substitution rate ratios along the *D. melanogaster* lineage.

| 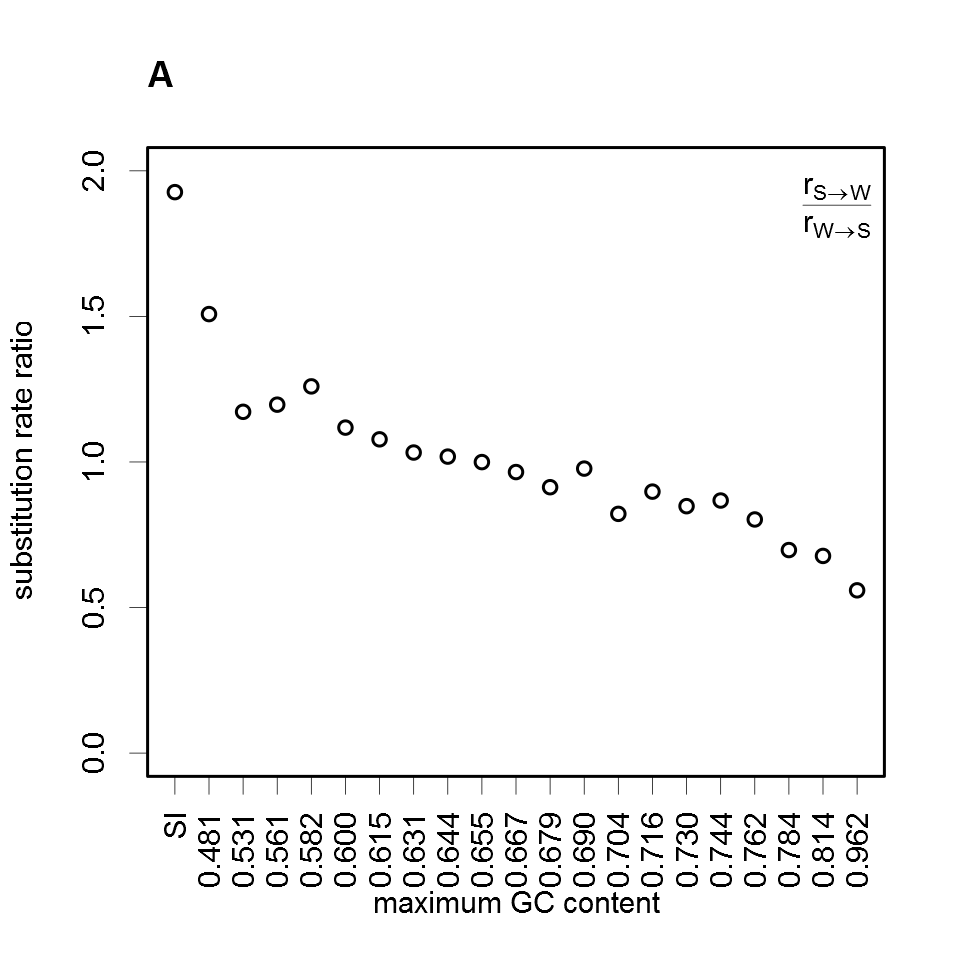 | 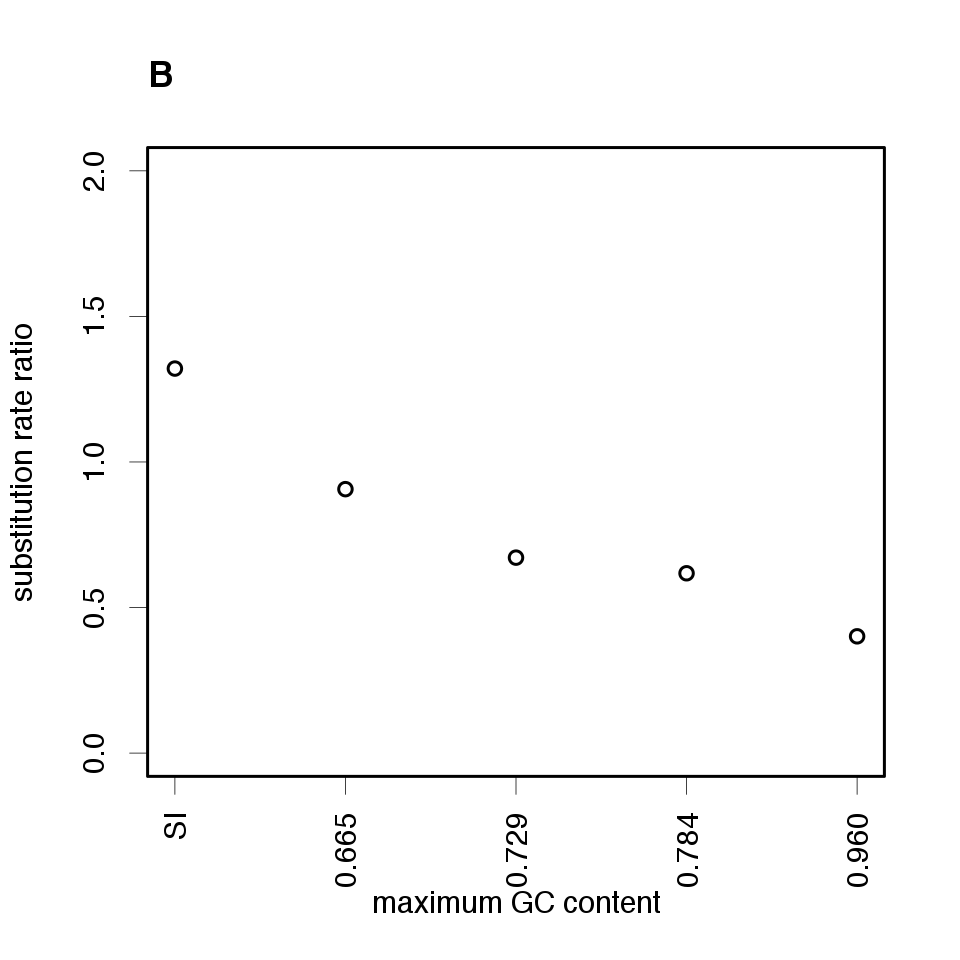 |
| --- | --- |
| 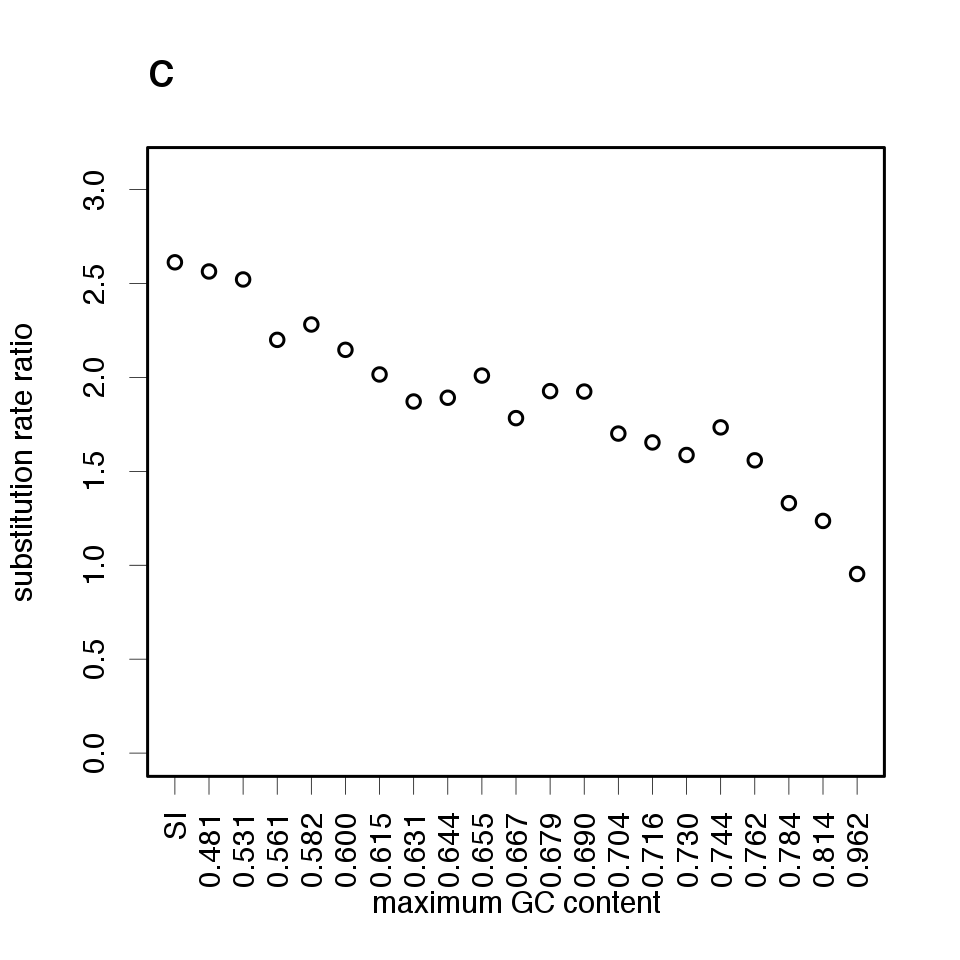 | 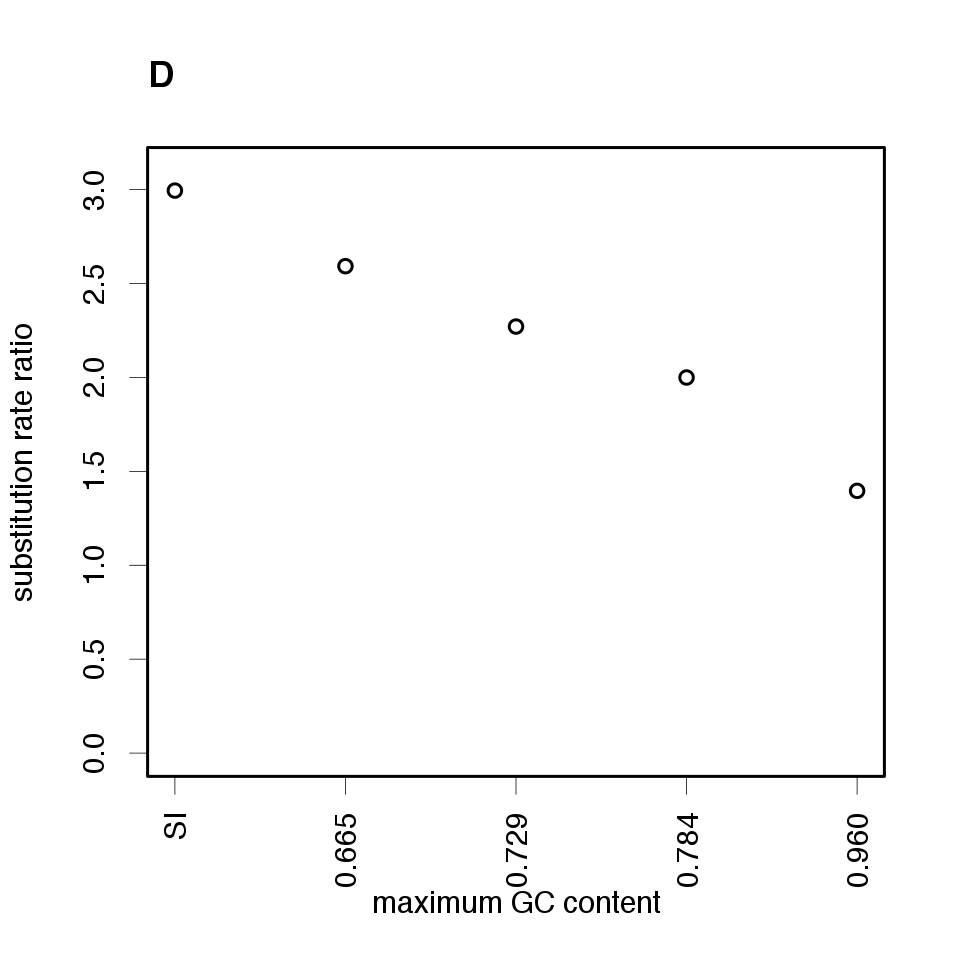 |

**Figure S6**. **Substitution rates using parsimony**. For positions 8-30bp of introns <66bp long (SI sites; leftmost points), and 4-fold degenerate sites (remaining points) binned by the GC content of the extant D. melanogaster reference sequence, for $AT\to GC$ substitutions (teal circles) and $GC\to AT$ substitutions (orange triangles), using parsimony to infer ancestral states. Panel A – autosomal substitution rates along the *D. simulans* lineage; panel B – X chromosome substitution rates along the *D. simulans* lineage; panel C – autosomal substitution rates along the *D. melanogaster* lineage; panel D – X chromosome substitution rates along the *D. melanogaster* lineage.

| 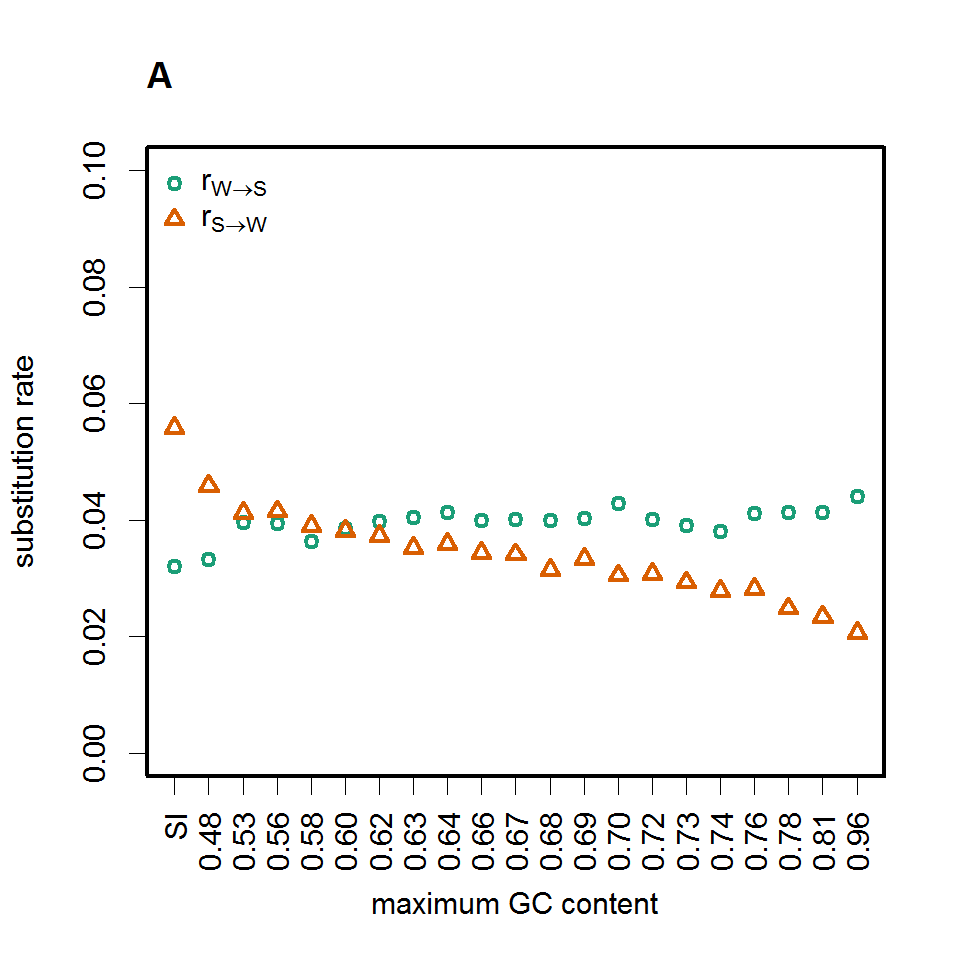 | 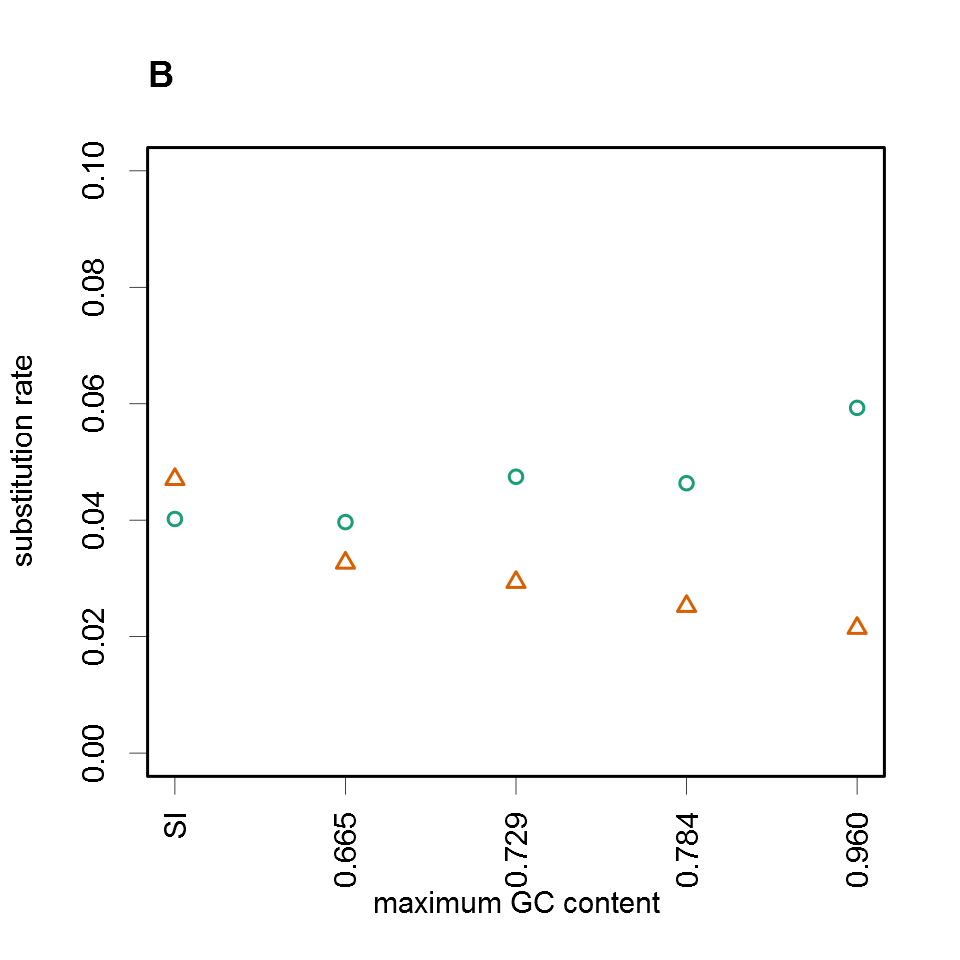 |
| --- | --- |
| 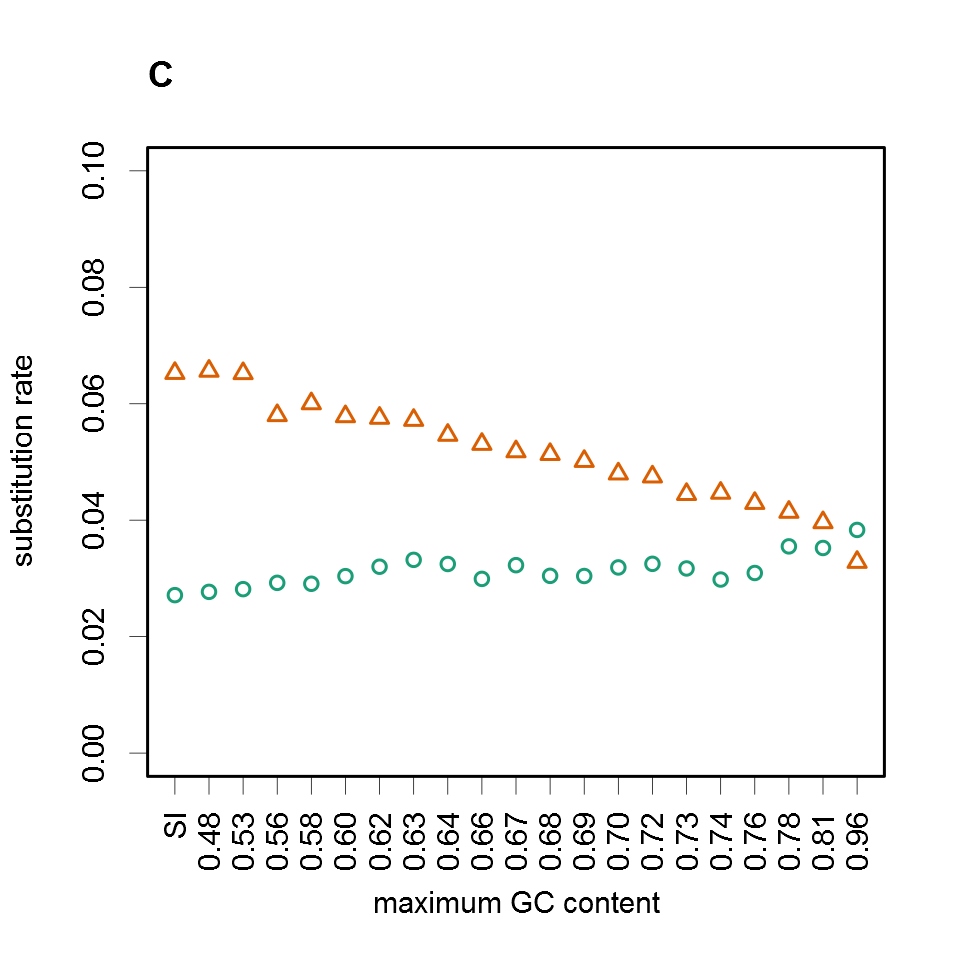 | 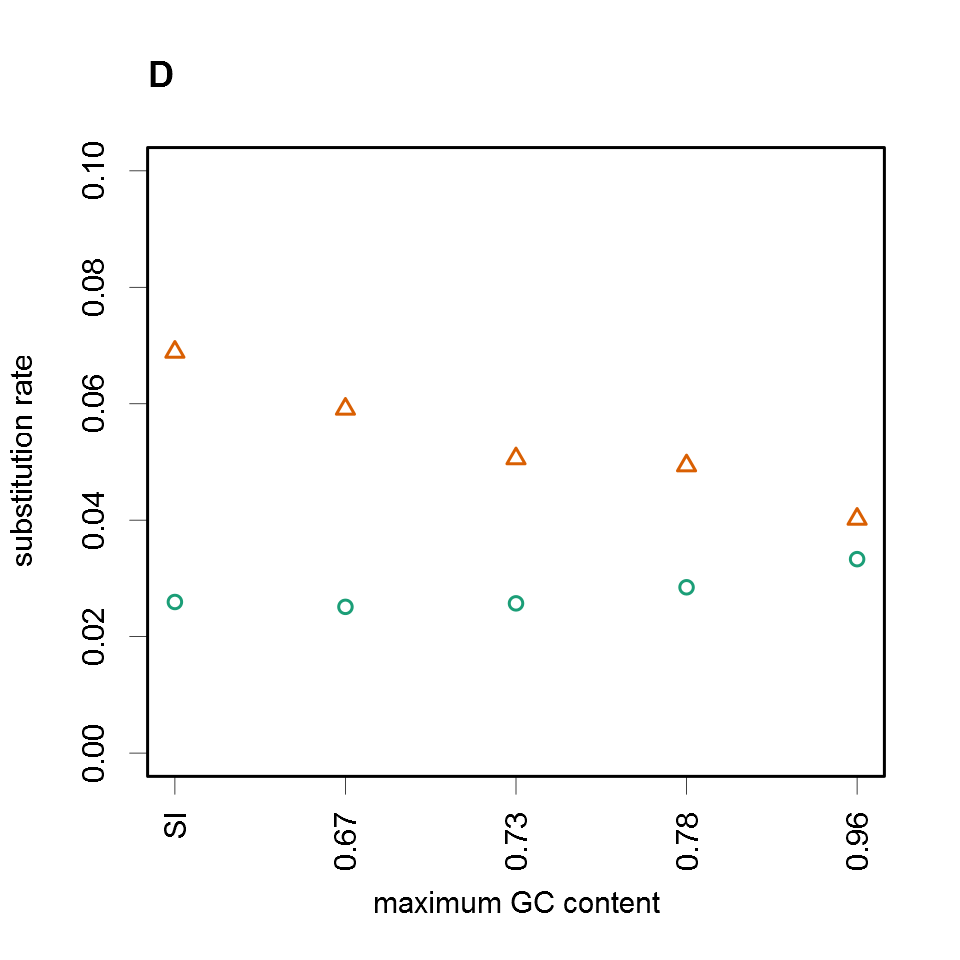 |

**Figure S7**. **The ratio of substitution counts using parsimony.** For positions 8-30bp of introns <66bp long (SI sites; leftmost points), and 4-fold degenerate sites (remaining points) binned by the GC content of the extant *D. melanogaster* reference sequence and using parsimony to infer ancestral states. Panel A – autosomal ratio of substitution counts along the *D. simulans* lineage; panel B – X chromosome ratio of substitution counts along the *D. simulans* lineage; panel C – autosomal ratio of substitution counts along the *D. melanogaster* lineage; panel D – X chromosome ratio of substitution counts along the *D. melanogaster* lineage.

| 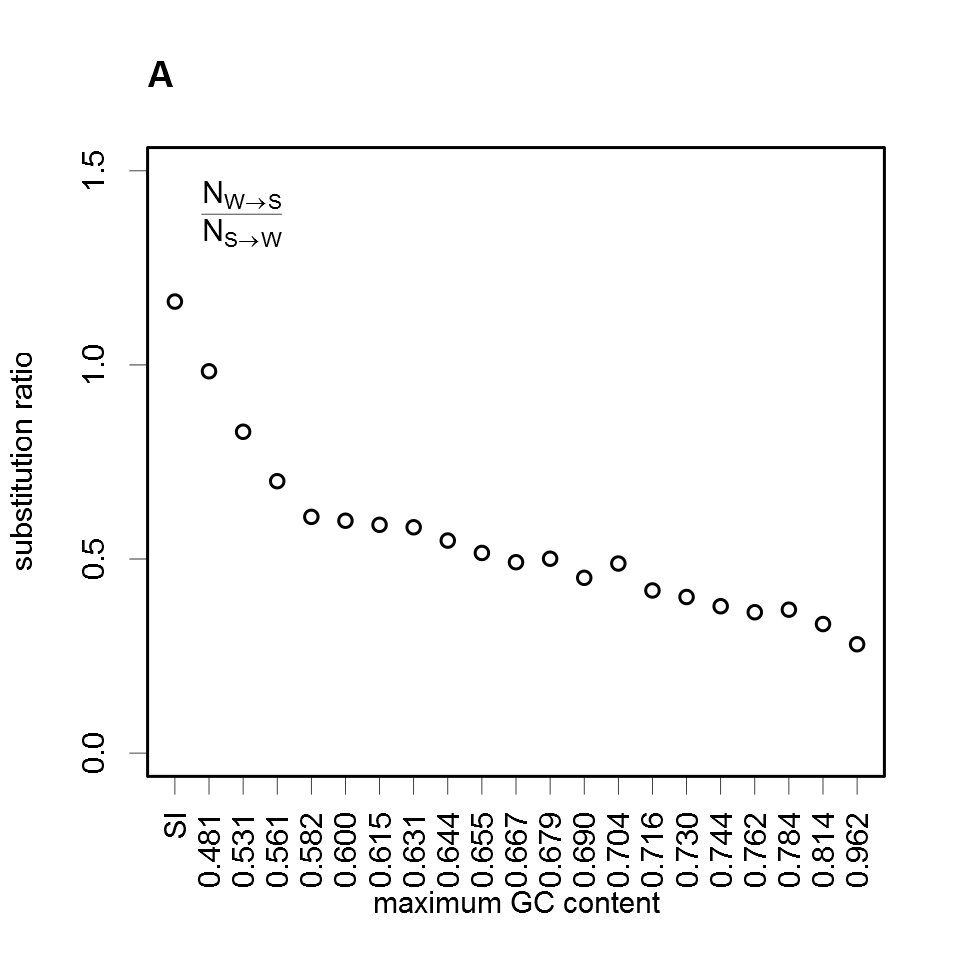 | 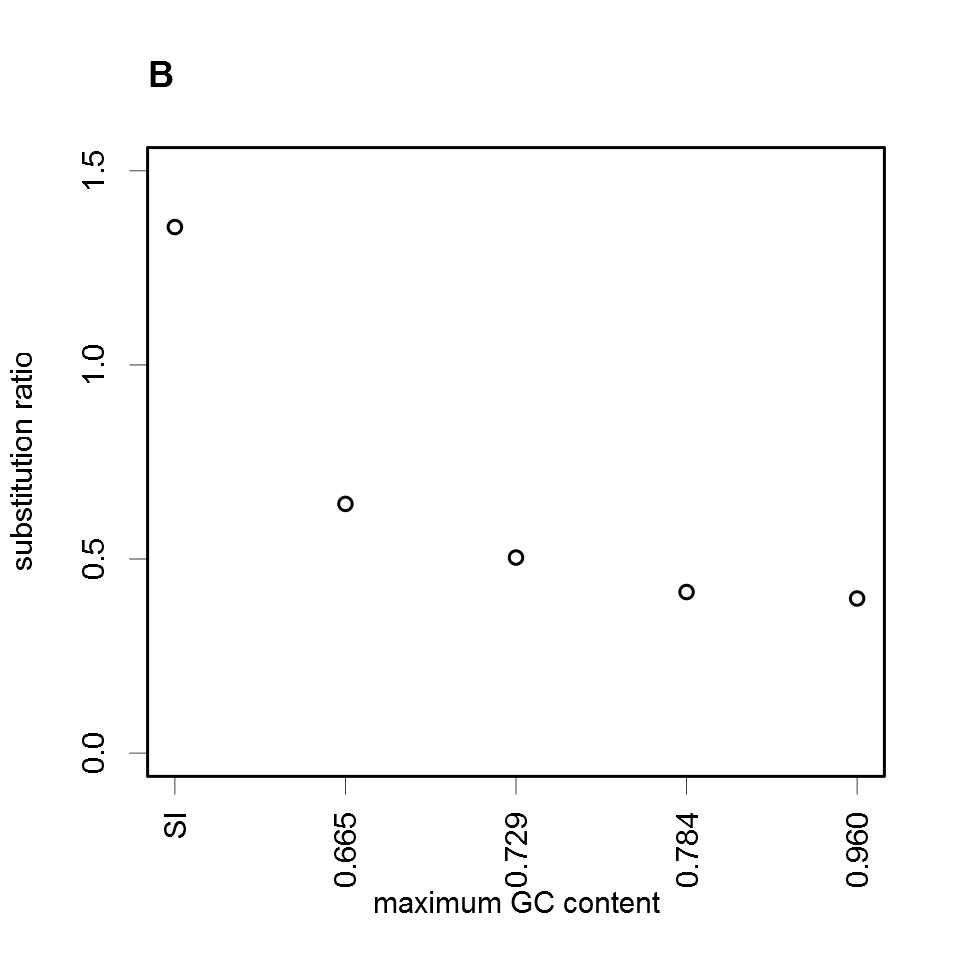 |
| --- | --- |
| 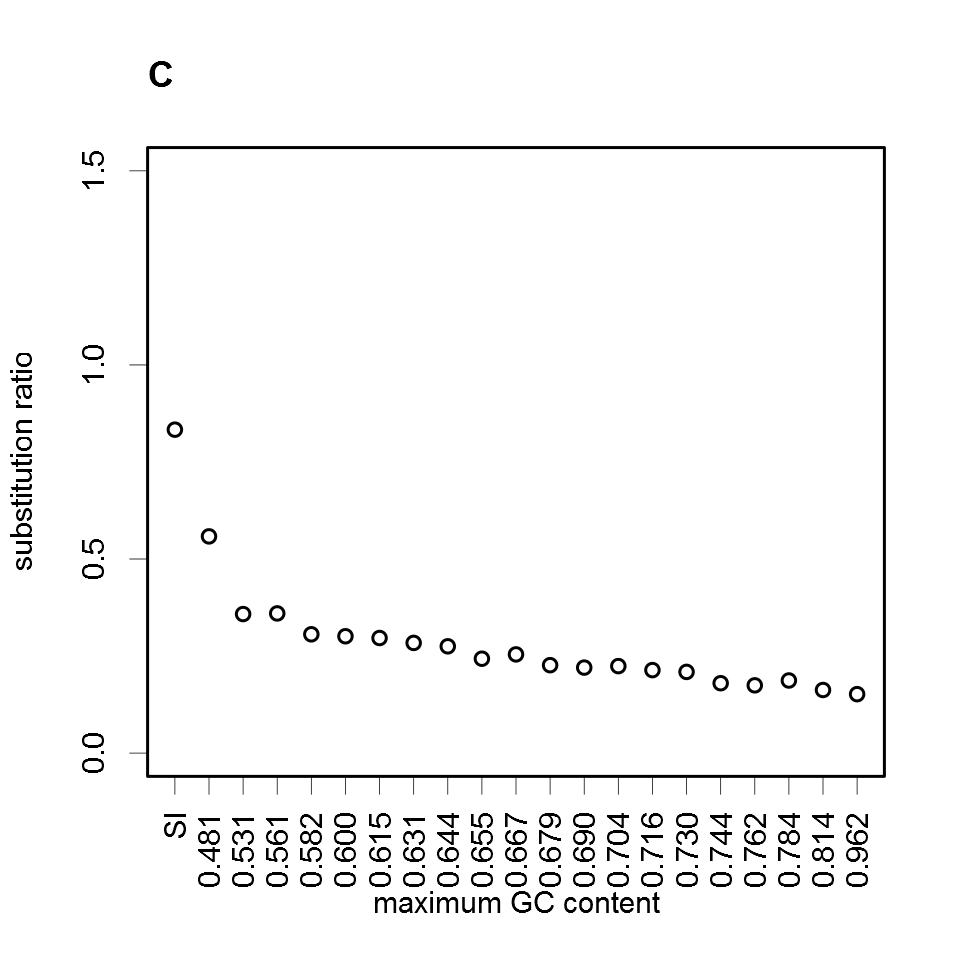 | 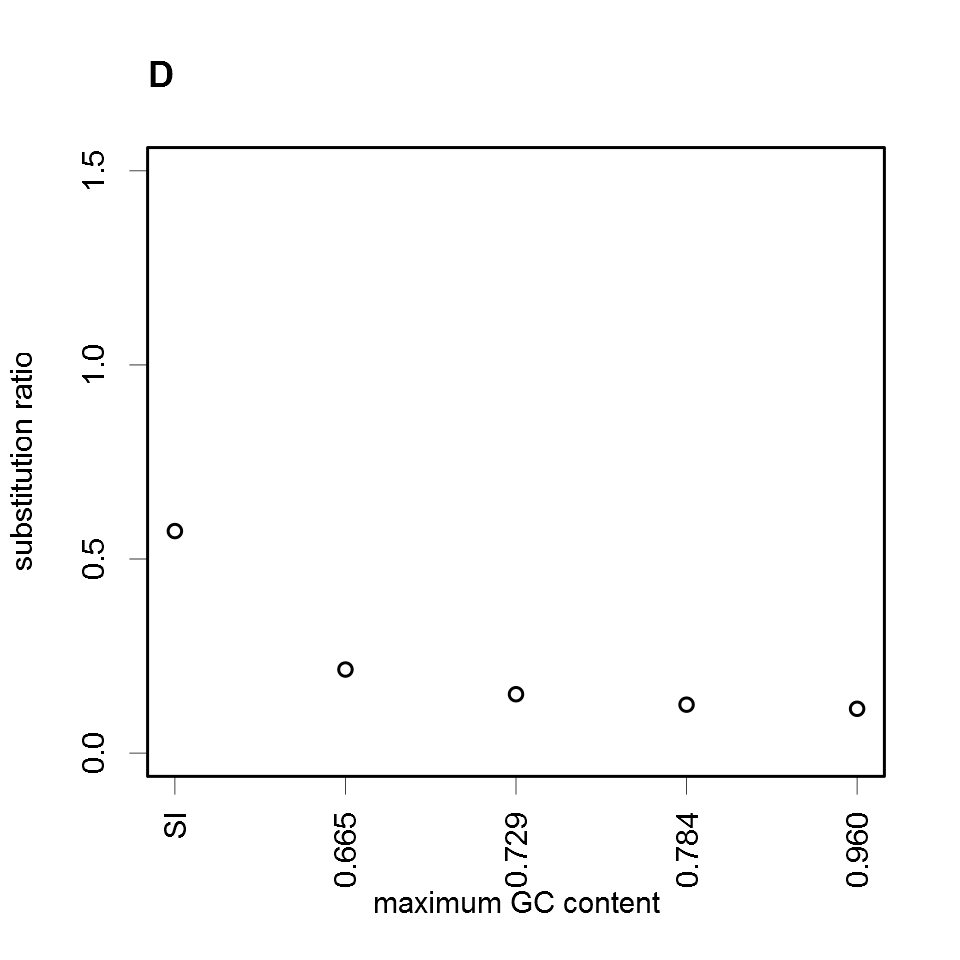 |

**Figure S8**. **Substitution rates using SBR.** For positions 8-30bp of introns <66bp long (SI sites; leftmost points), and 4-fold degenerate sites (remaining points) binned by the GC content of the extant *D. melanogaster* reference sequence, for $AT\to GC$ substitutions (teal circles) and $GC\to AT$ substitutions (orange triangles), using SBR to infer ancestral states. Panel A – autosomal substitution rates along the *D. simulans* lineage; panel B – X chromosome substitution rates along the *D. simulans* lineage; panel C – autosomal substitution rates along the *D. melanogaster* lineage; panel D – X chromosome substitution rates along the *D. melanogaster* lineage.

| 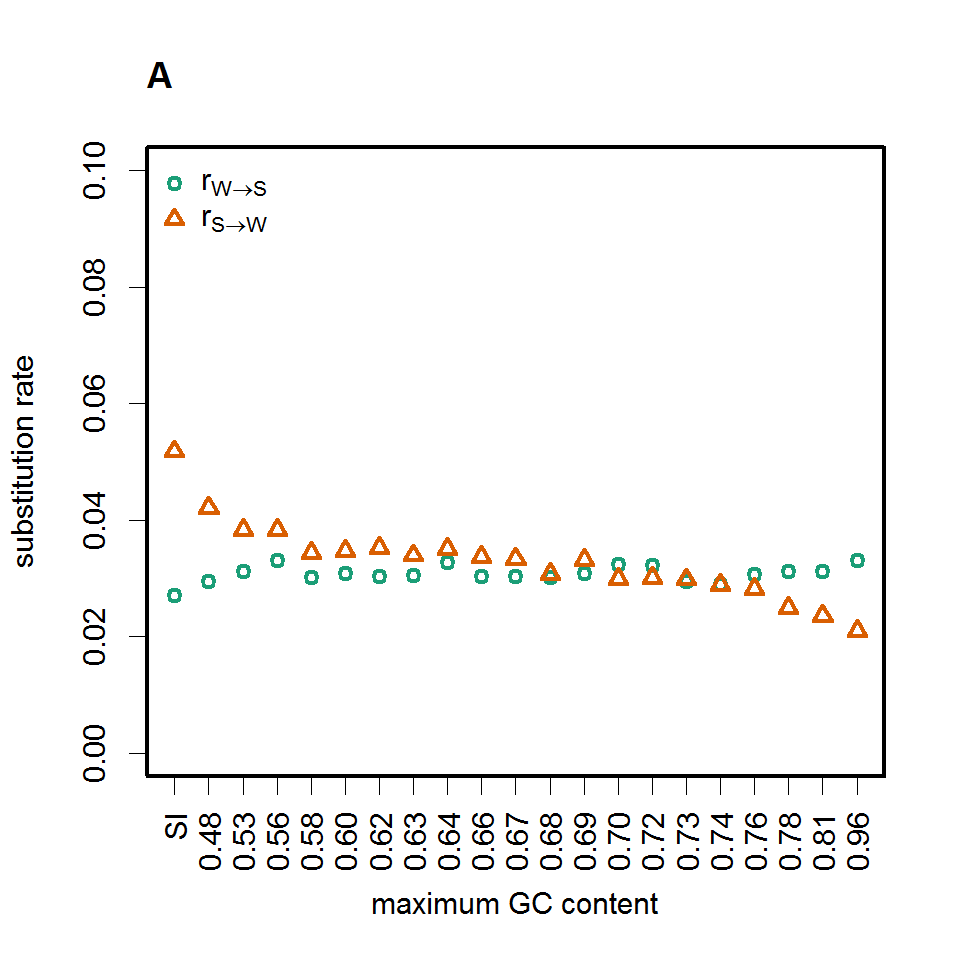 | 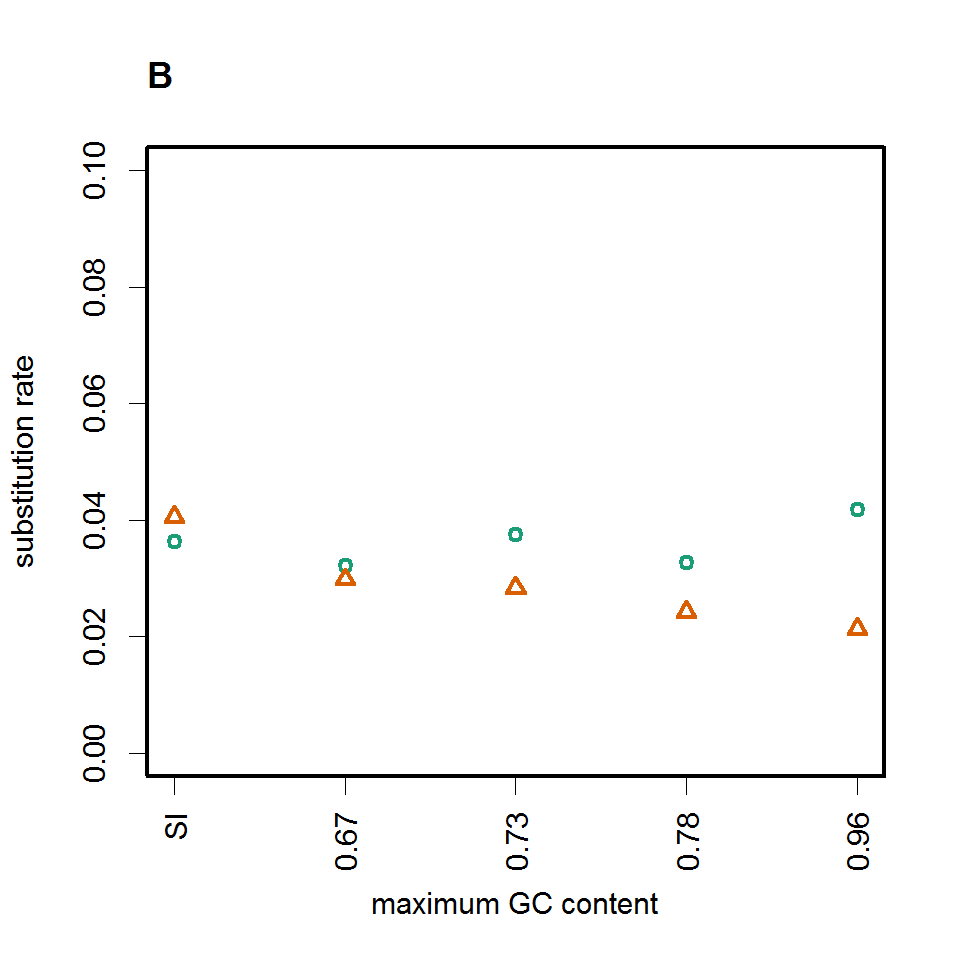 |
| --- | --- |
| 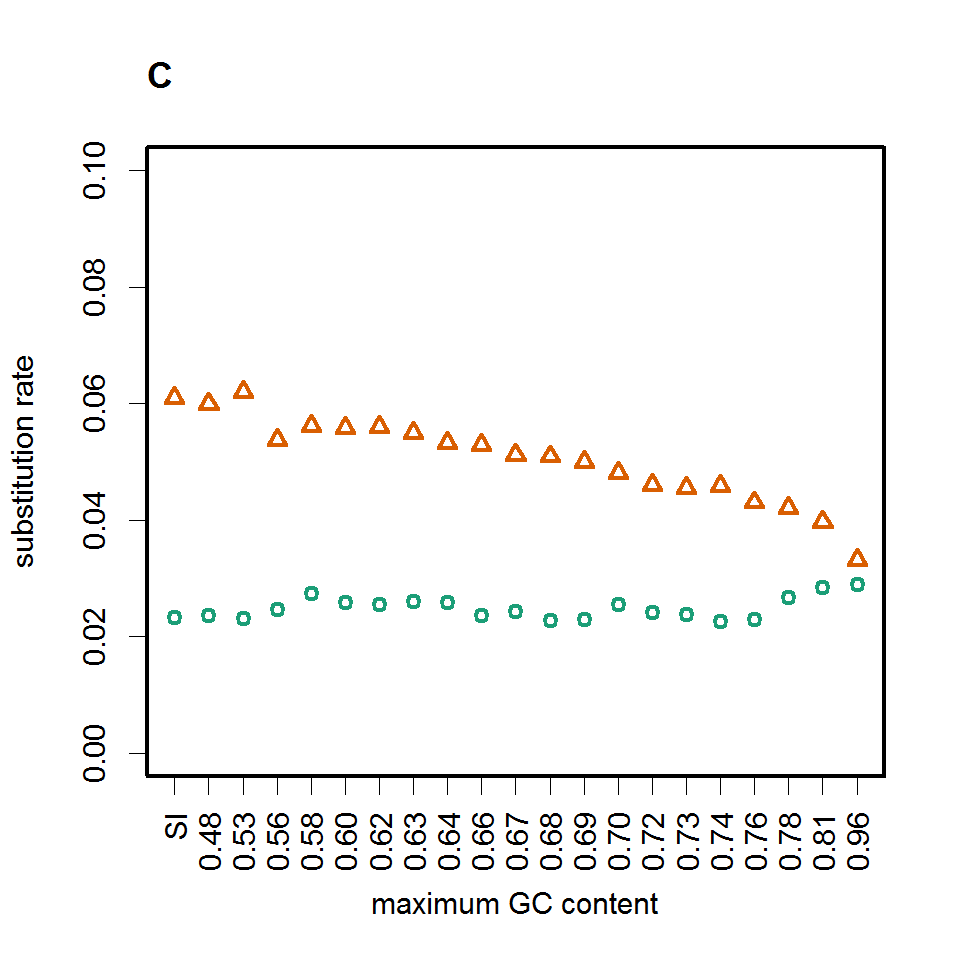 | 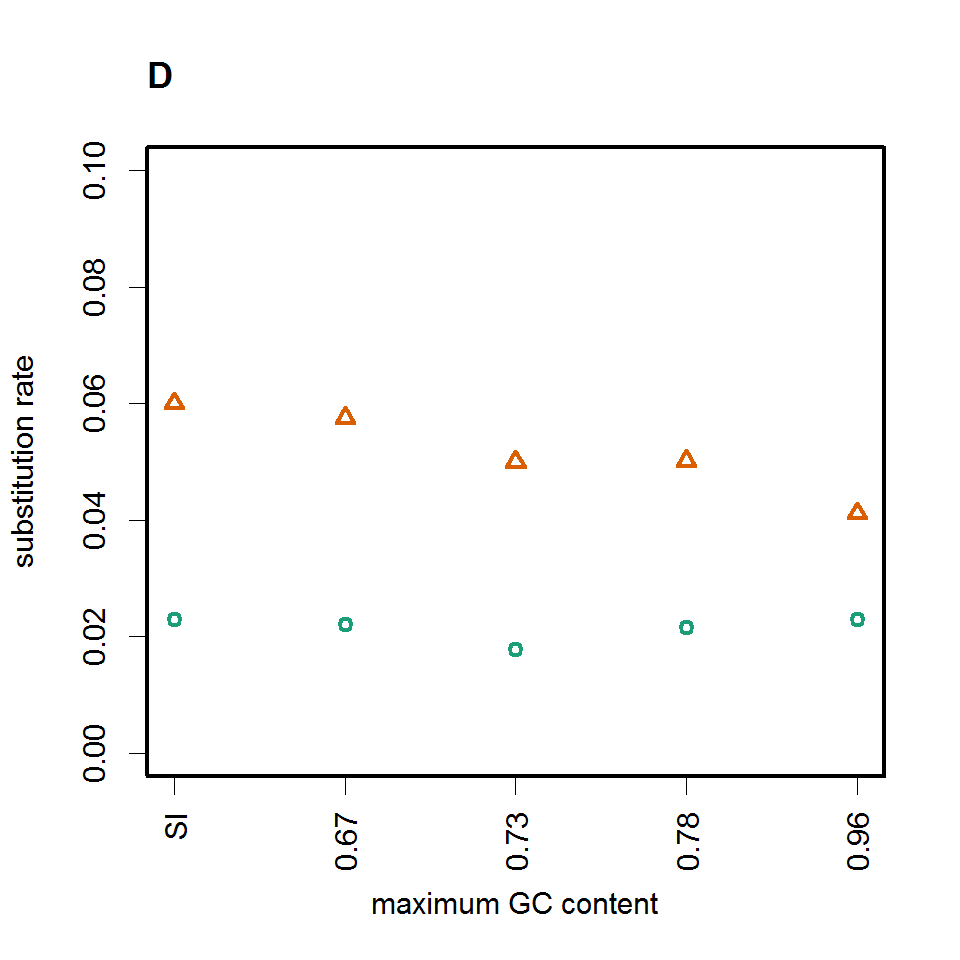 |

**Figure S9**. **The ratio of substitution counts using SBR.** For positions 8-30bp of introns <66bp long (SI sites), and 4-fold degenerate sites binned by the GC content of the extant *D. melanogaster* reference sequence and using SBR to infer ancestral states. Panel A – autosomal ratio of substitution counts along the *D. simulans* lineage; panel B – X chromosome ratio of substitution counts along the *D. simulans* lineage; panel C – autosomal ratio of substitution counts along the *D. melanogaster* lineage; panel D – X chromosome ratio of substitution counts along the *D. melanogaster* lineage.

| 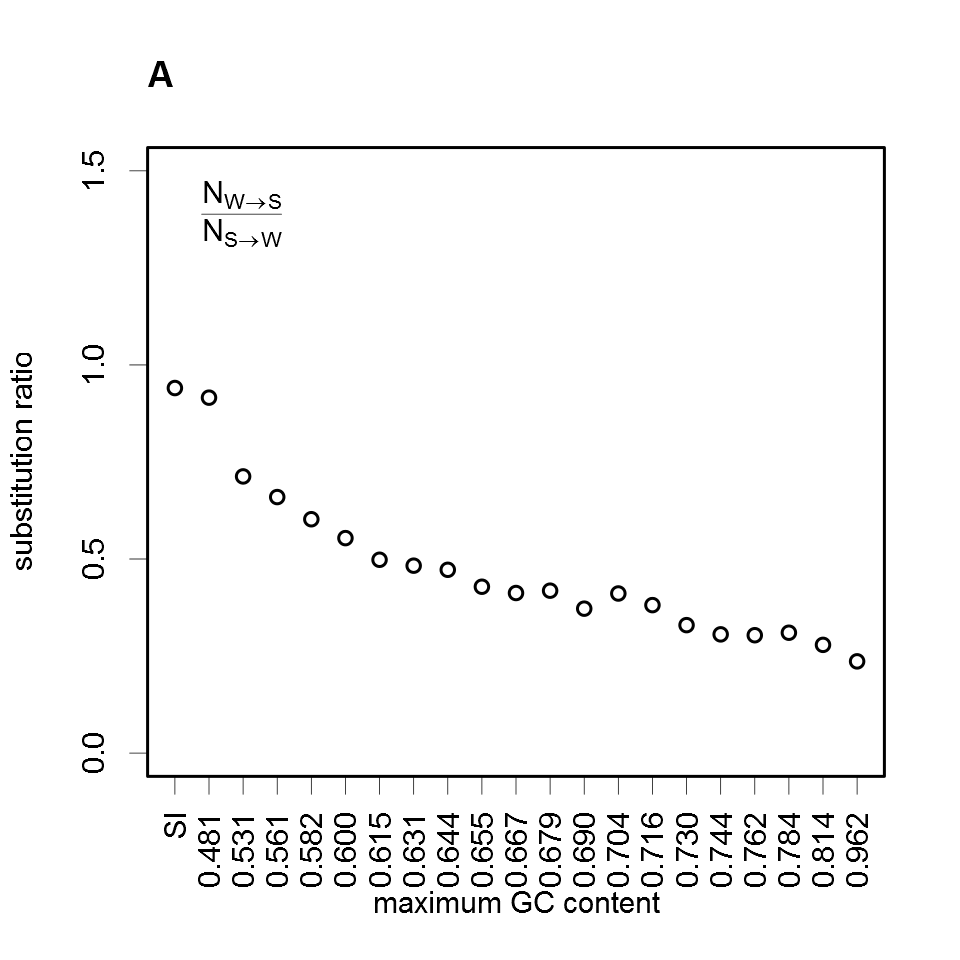 | 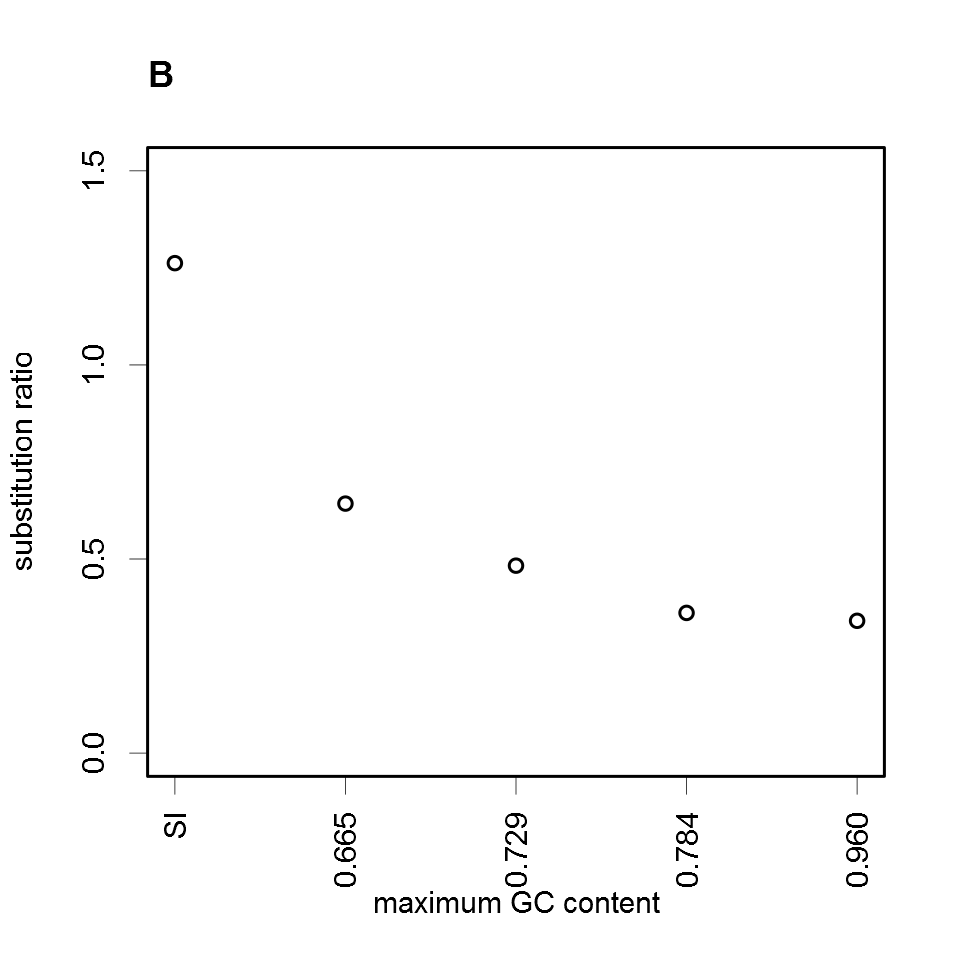 |
| --- | --- |
| 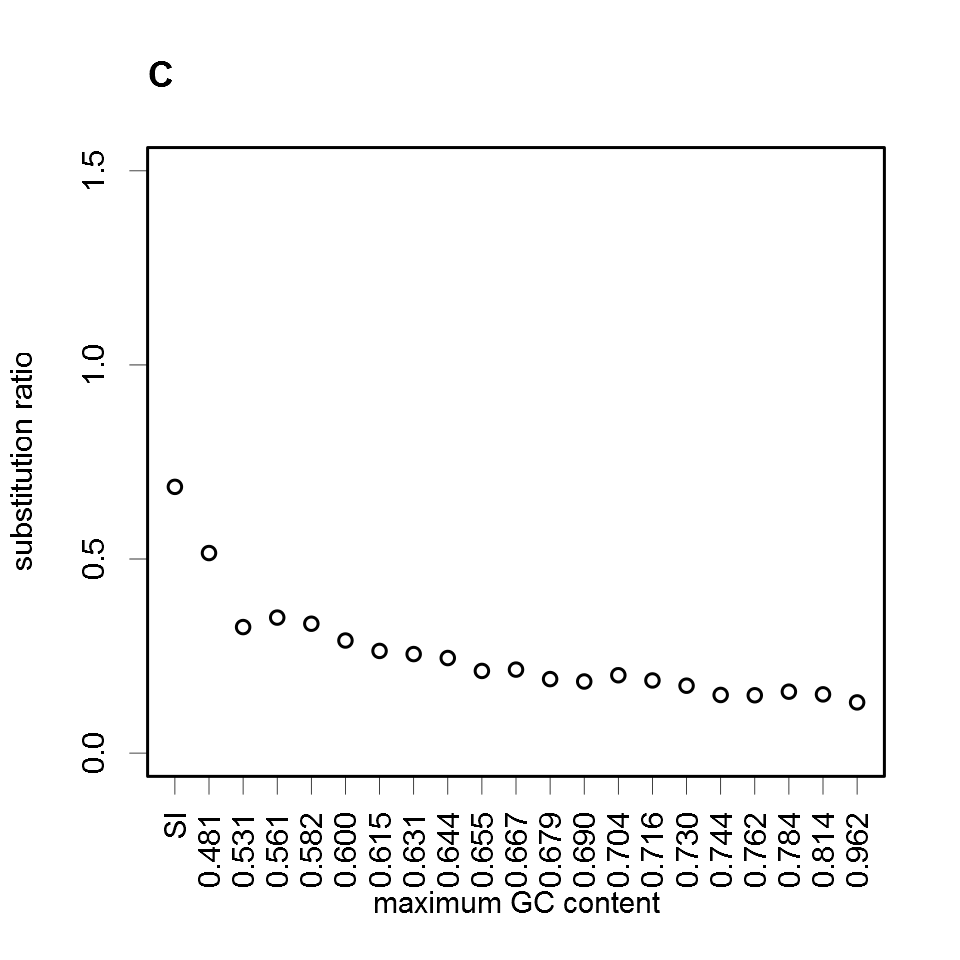 | 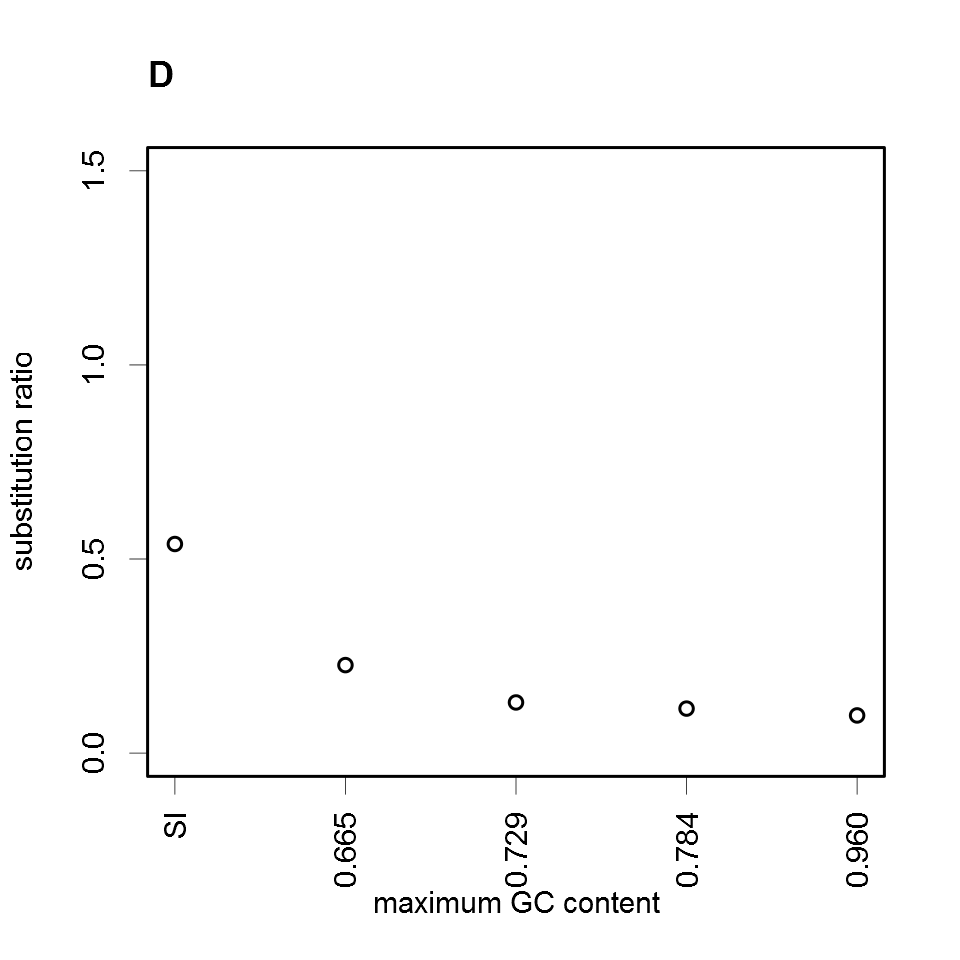 |

**Figure S10. Mean derived allele frequencies using parsimony.** For positions 8-30bp of introns <66bp long (SI sites; leftmost points), and 4-fold degenerate sites (remaining points) binned by the GC content of the extant *D. melanogaster* reference sequence, and using parsimony to infer ancestral states. $AT\to GC$ mutations – teal circles; $GC\to AT$ mutations – orange triangles; $AT\to AT$ mutations or $GC\to GC$ mutations – lilac squares. Panel A – autosomal DAFs in the Madagascan (MD) *D. simulans* sample; panel B – X chromosome DAFs in the MD *D. simulans* sample; panel C – autosomal DAFs in the Rwandan (RG) *D. melanogaster* sample; panel D – X chromosome DAFs in the RG *D. melanogaster* sample.

| 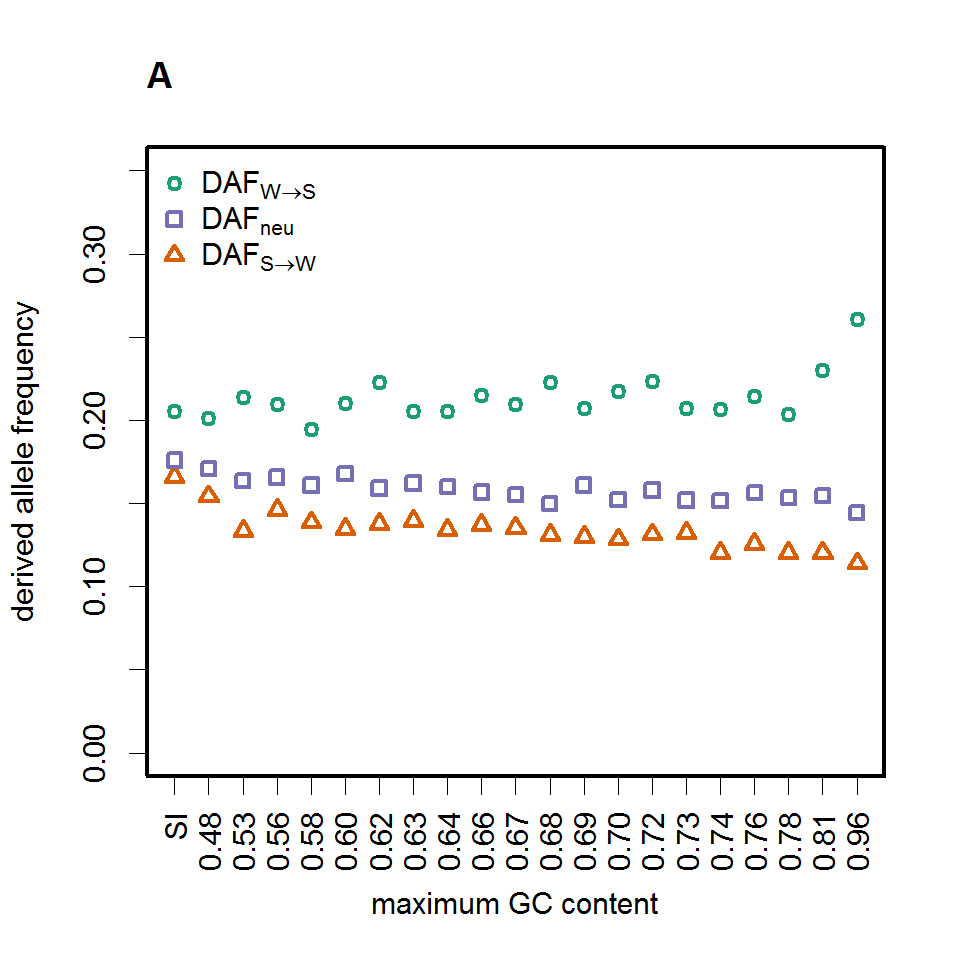 | 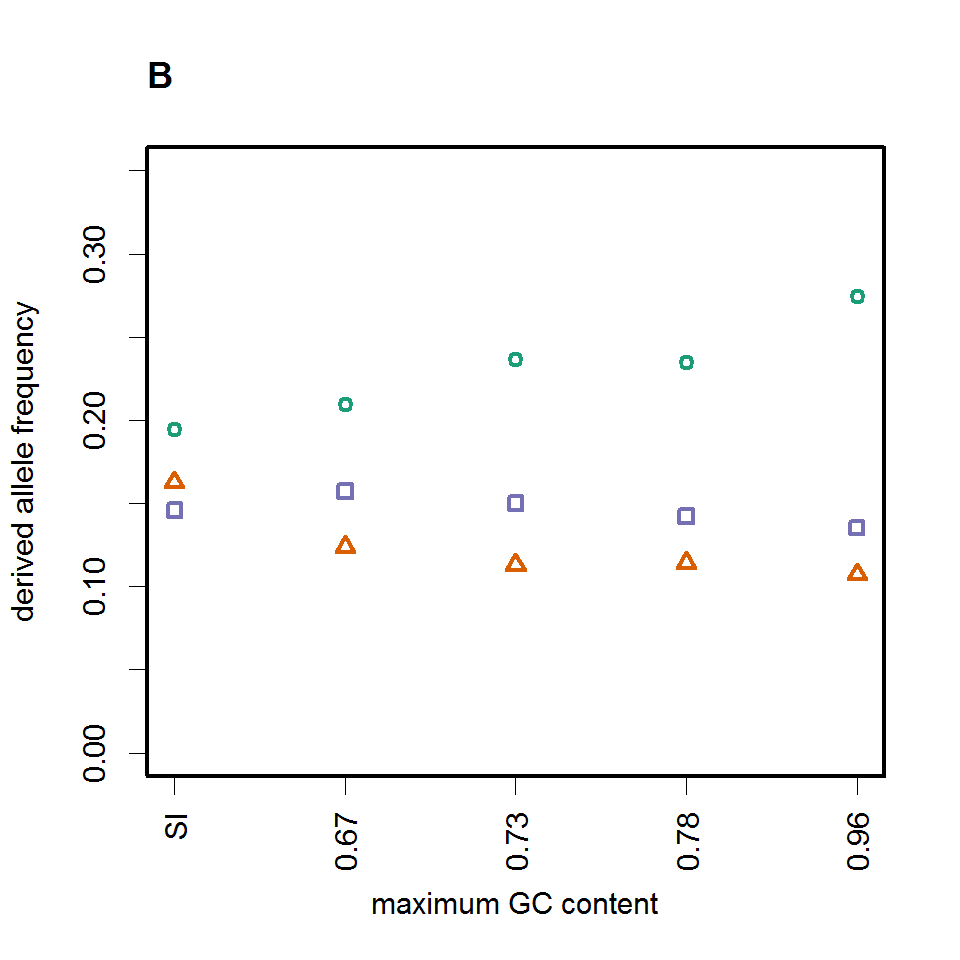 |
| --- | --- |
| 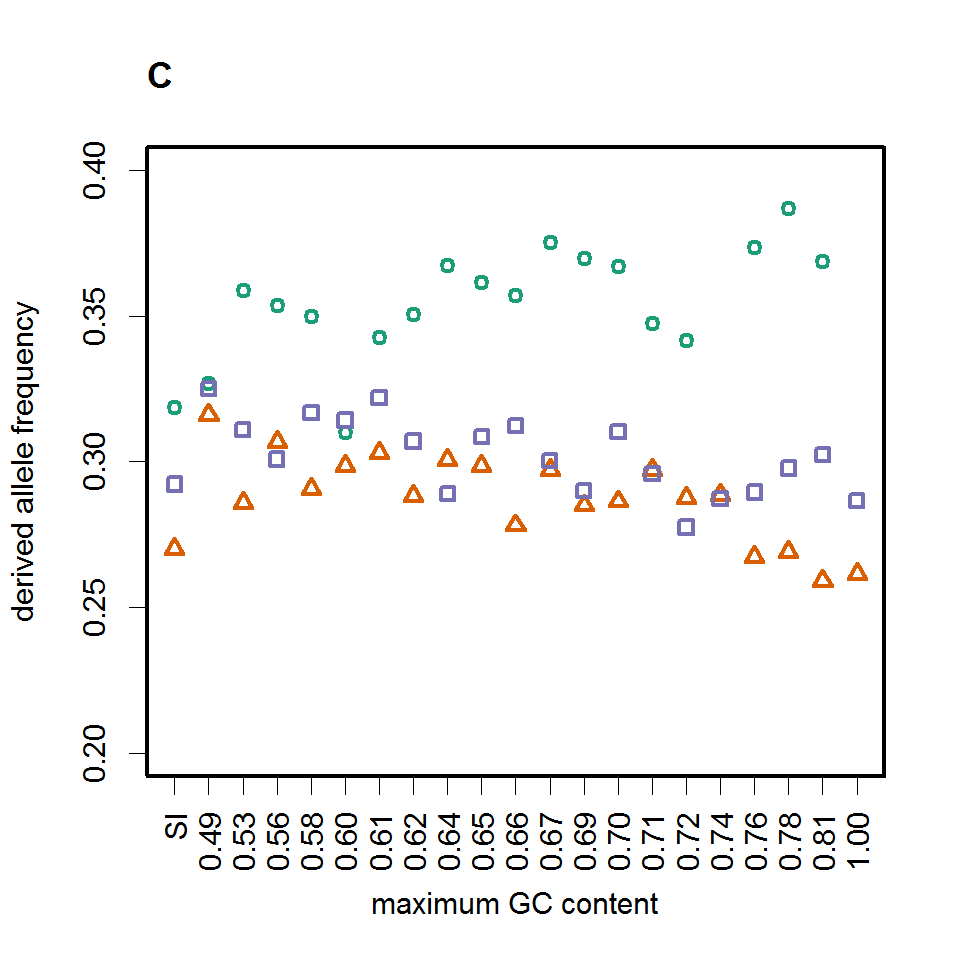 | 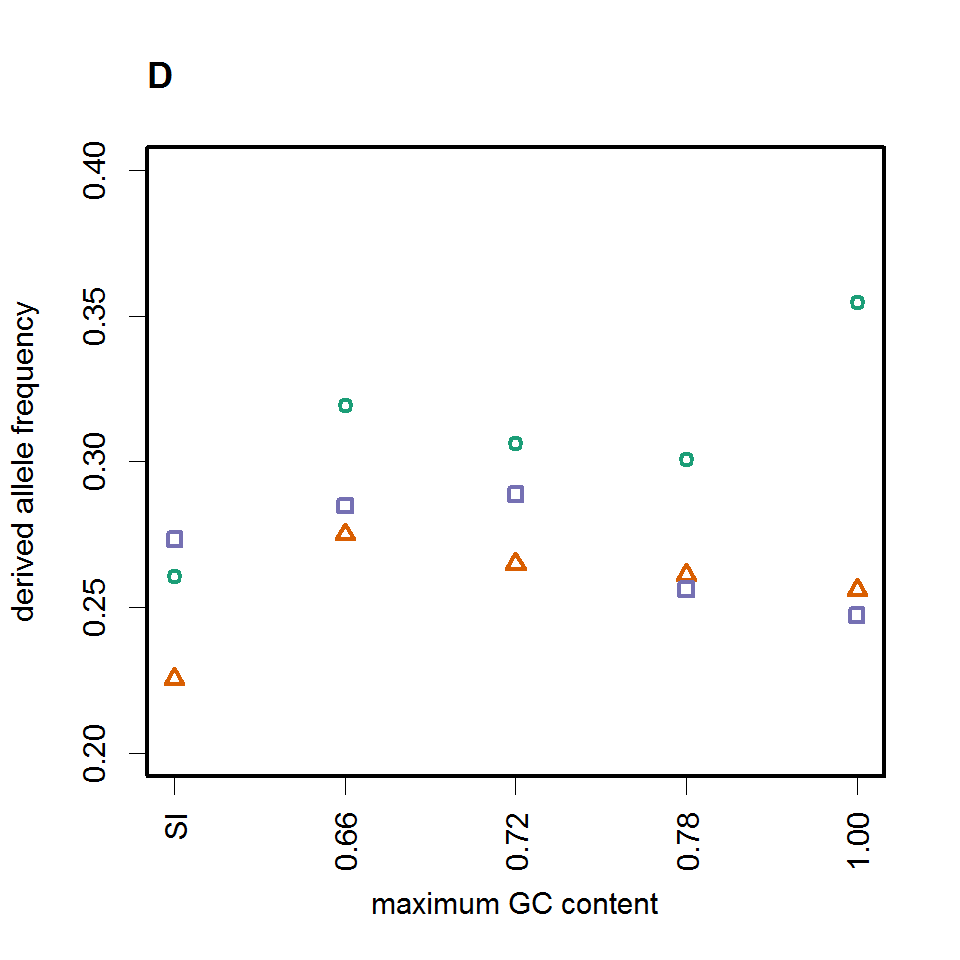 |

**Figure S11. Mean derived allele frequencies using SBR.** For positions 8-30bp of introns <66bp long (SI sites; leftmost points), and 4-fold degenerate sites (remaining points) binned by the GC content of the extant *D. melanogaster* reference sequence, and using SBR to infer ancestral states. $AT\to GC$ mutations – teal circles; $GC\to AT$ mutations – orange triangles; $AT\to AT$ mutations or $GC\to GC$ mutations – lilac squares. Panel A – autosomal DAFs in the Madagascan (MD) *D. simulans* sample; panel B – X chromosome DAFs in the MD *D. simulans* sample; panel C – autosomal DAFs in the Rwandan (RG) *D. melanogaster* sample; panel D – X chromosome DAFs in the RG *D. melanogaster* sample.

| 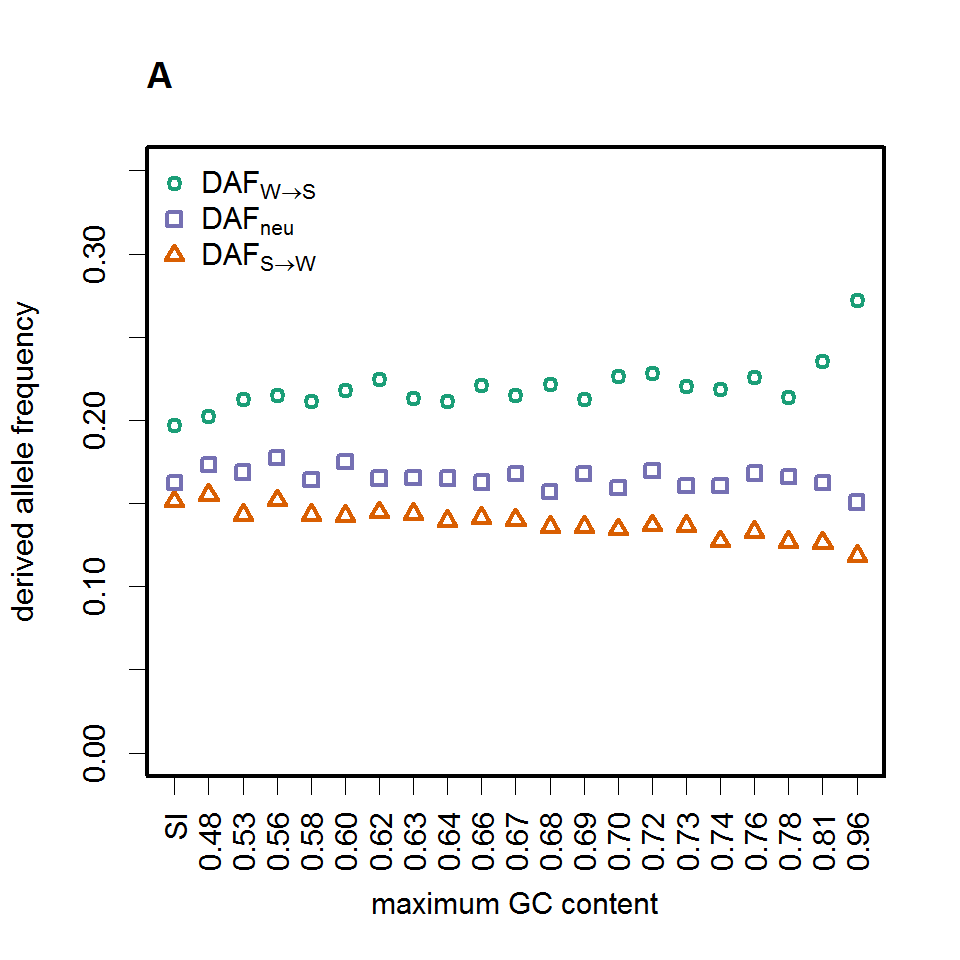 | 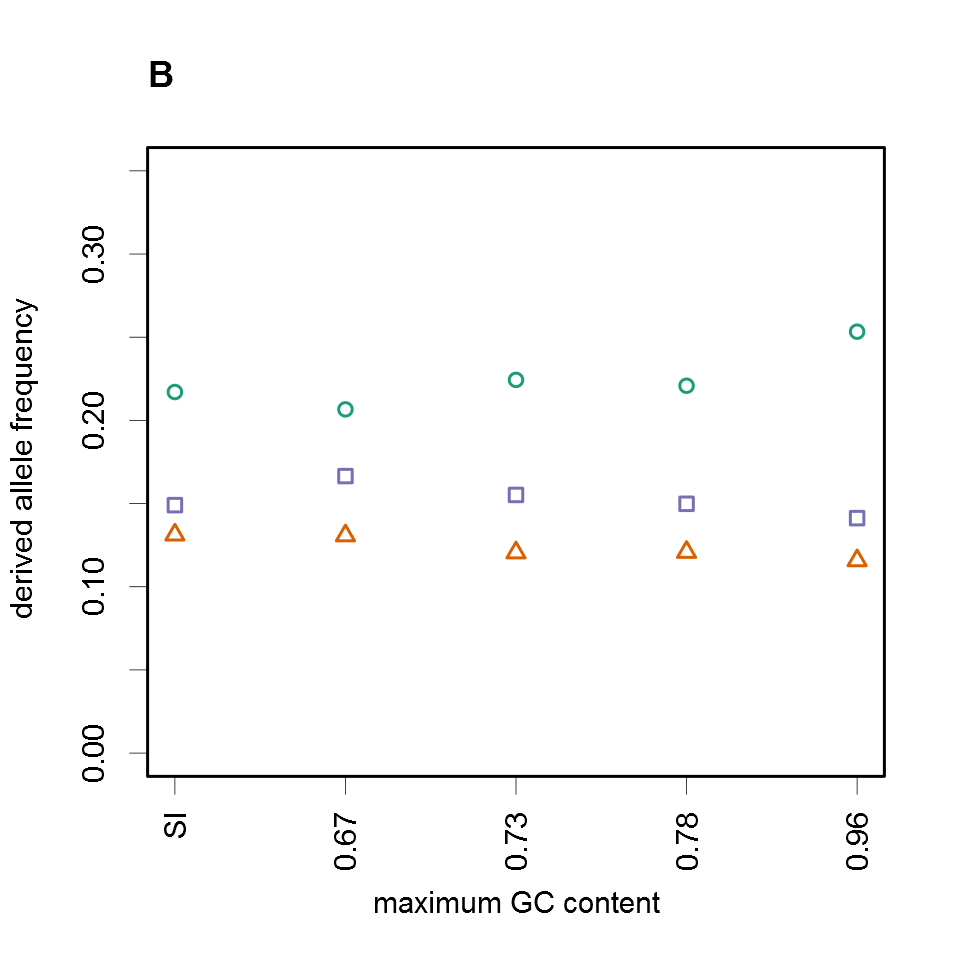 |
| --- | --- |
| 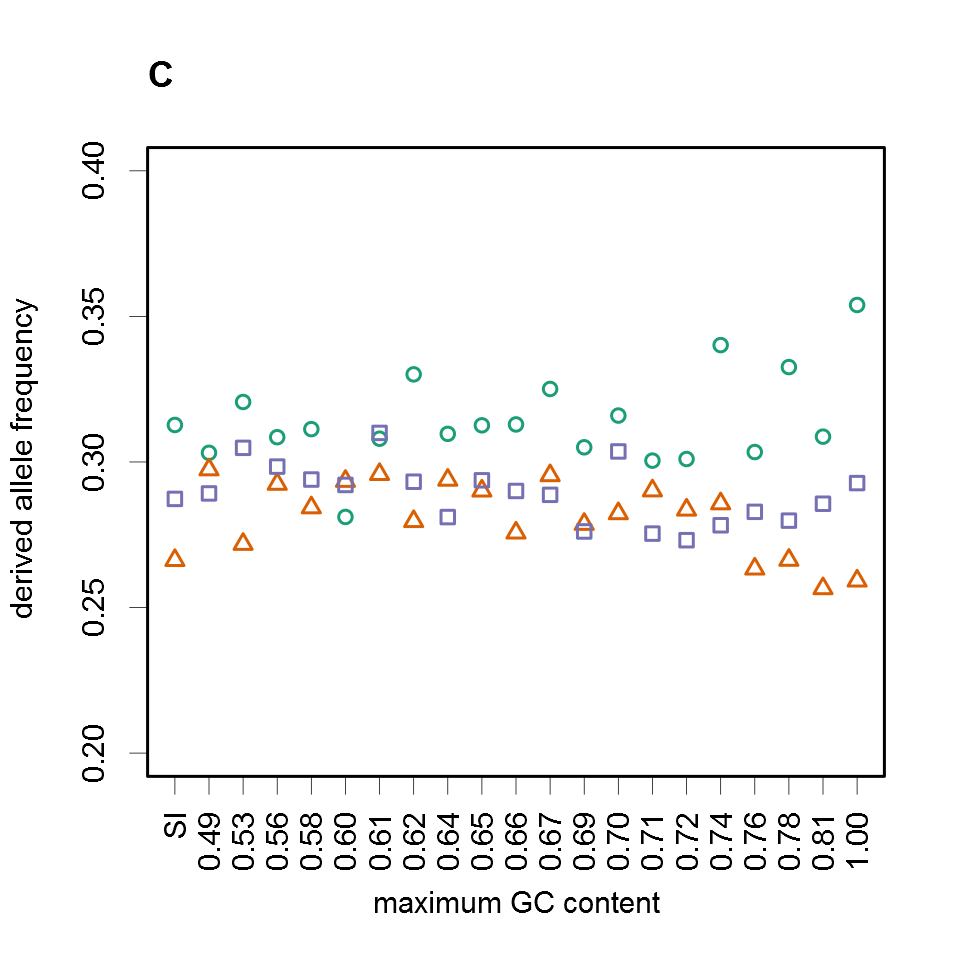 | 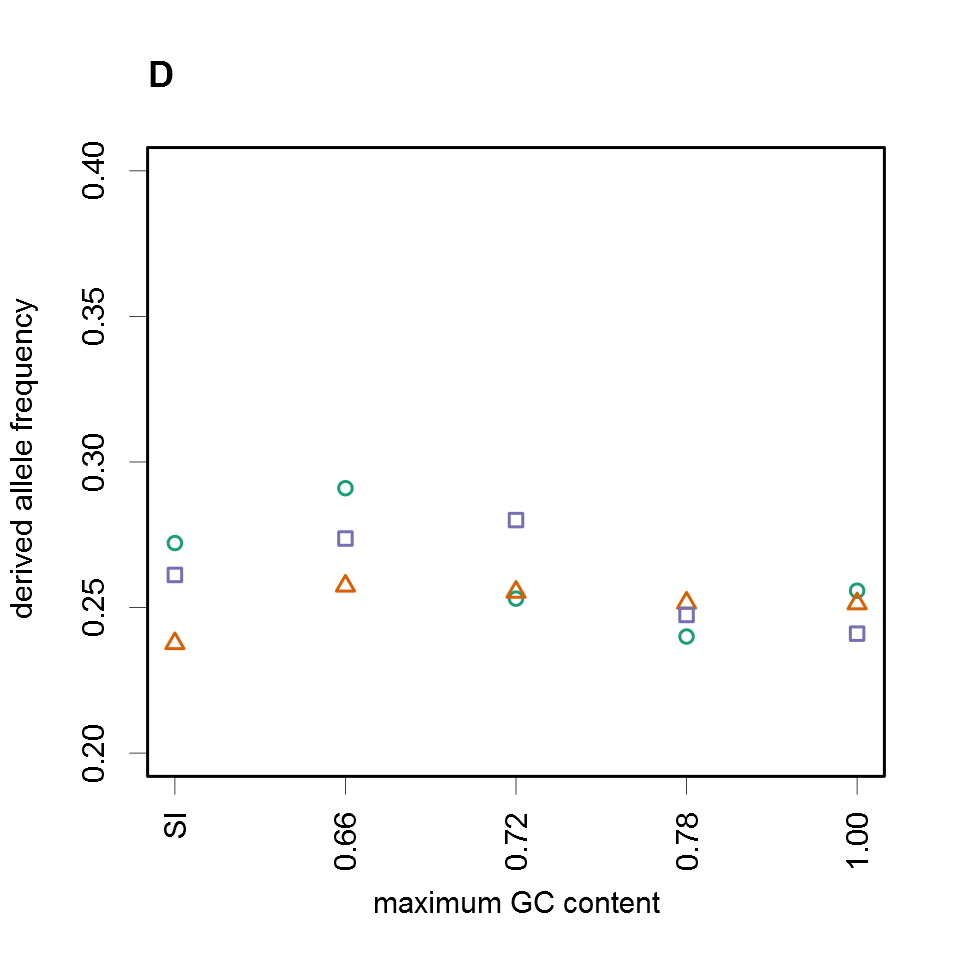 |

**Figure S12. Estimates of the mutation bias parameter, *κ*.** Estimated magnitude of mutation bias towards AT alleles ($\kappa=u/v,$ where $u$ is the rate at which S alleles mutate to W alleles, and $v$ is the mutation rate in the opposite direction) for positions 8-30bp of introns <66bp long (SI sites; leftmost points), and 4-fold degenerate sites (remaining points), binned as described for Fig 1 in the main text. $\kappa$ was estimated using the MD (Madagascan) sample of *D. simulans* (top row) and the RG (Rwandan) sample of *D. melanogaster* (bottom row), for autosomes (left-hand column) and X-linked sites (right-hand column). Two methods were used: the method of Zeng and Charlesworth (2009) with a one-step size in population size (ZC in the main text) – green circles; and the method of Glémin et al. (2015), not incorporating polarisation errors (M1 in the main text) – pink squares.

| 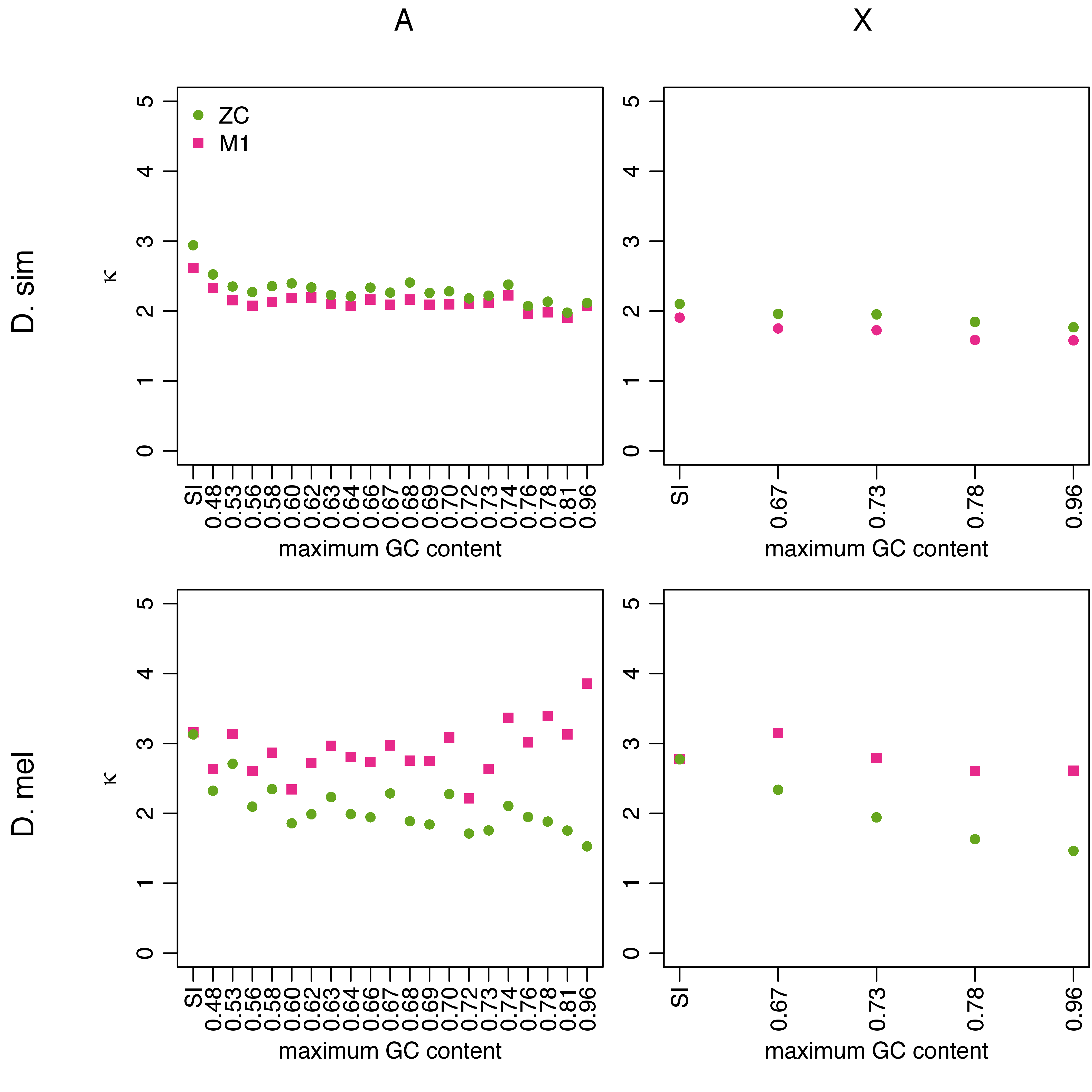 |
| --- |

**Figure S13. A comparison of estimates of *γ* from autosomal *D. melanogaster* sites with different numbers of bins.** For Rwandan (RG) *D. melanogaster* autosomal 4-fold degenerate sites, with 20 GC content bins (Panel A) and reduced to 10 GC content bins (Panel B). *γ* was calculated using the method of Zeng and Charlesworth (2009) under equilibrium – yellow circles; the method of Zeng and Charlesworth (2009) incorporating a one-step change in population size – green circles; and the method of Glémin et al. (2015), not incorporating polarisation errors – pink squares. Filled points – bins where a model with $\gamma\neq0$ fitted best; open points – bins where a model with $\gamma=0$fitted best.

| 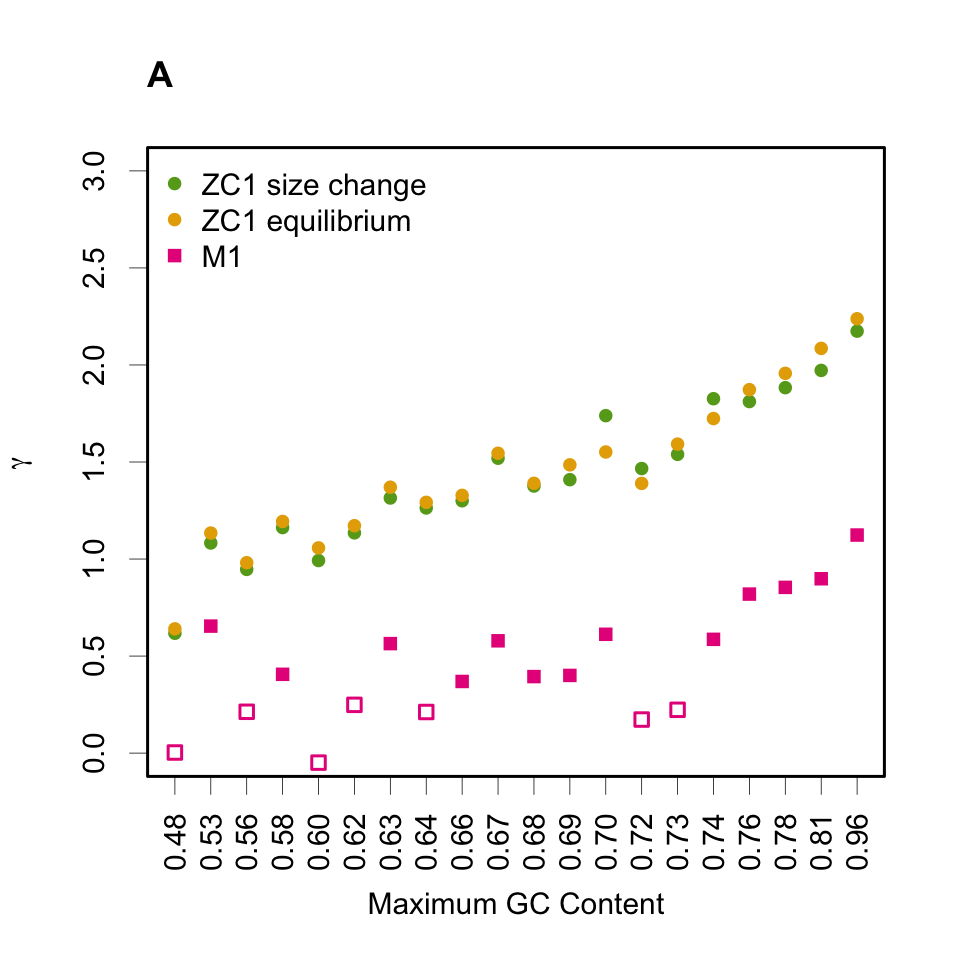 | 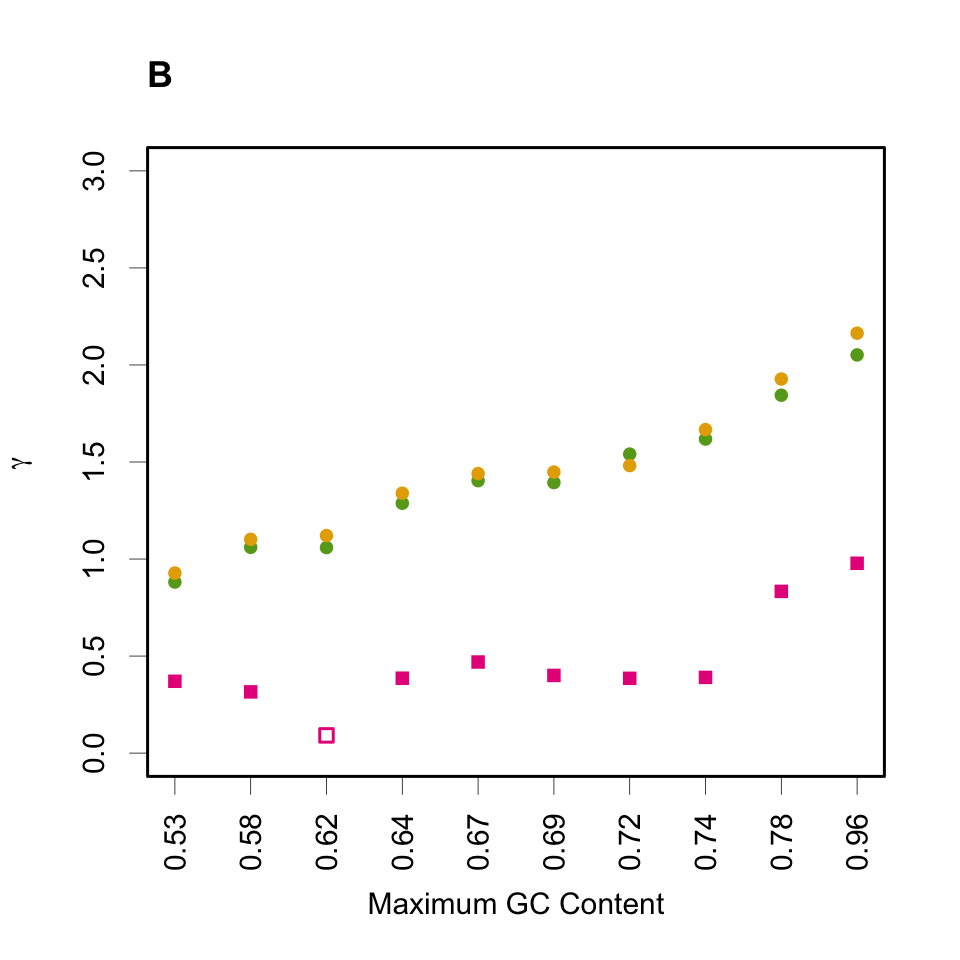 |
| --- | --- |

**Figure S14. The proportions of singletons in the SFSs of S>W and W>S mutations after a population size reduction.** We assumed that the population was initially at equilibrium. The parameters scaled by this population size were *γ* = 0.5, *κ* = 2.5, *θ* = 0.02 (cf., the estimates based on the Glémin method in Figure 4). At time 0, its size reduced instantly 40-fold. We used the matrix method developed by Zeng and Charlesworth (2009) to obtain patterns of polymorphism after the size reduction. The mean polarisation error rates, as estimated by the Glémin method from the *D. melanogaster* dataset, were *e_SW_* = 0.65% and *e_WS_* = 3.85% (black curve), where *e_SW_* or *e_WS_* is the rate that the ancestral state of a $S\to W$ or $W\to S$ mutation is wrongly identified. Because these error rates were based on ancestral sequences reconstructed by the GTR-NH_b_ model, which is likely to be more accurate than those reconstructed by maximum parsimony, we also examined the effects of higher error rates with *e_SW_* = 1.3%, and *e_WS_* = 7.7% (red curve). It can be seen that, after the population size reduction, the proportion of singletons in the SFS for $S\to W$mutations becomes very close to that in the SFS for $W\to S$ mutations in the absence of polarisation error (blue curve), as a result of the reduction in selective pressure. This, when combined with a higher probability of incorrectly identifying the ancestral states for $W\to S$ mutations, can lead to the proportion of singletons in the SFS for $S\to W$ changes being lower than that for $W\to S$ changes, even though there has been weak selection favouring *S* (see the black and red curves for low and high rates of polarisation errors, respectively). These results highlight the importance of taking into account the effects of demography when SFS-based summary statistics are used. The fluctuations that appeared shortly after the reduction in population size are due to complex interactions involving non-equilibrium base composition, mutational bias, and relaxed selection, as noted previously by Zeng and Charlesworth (2009).

| **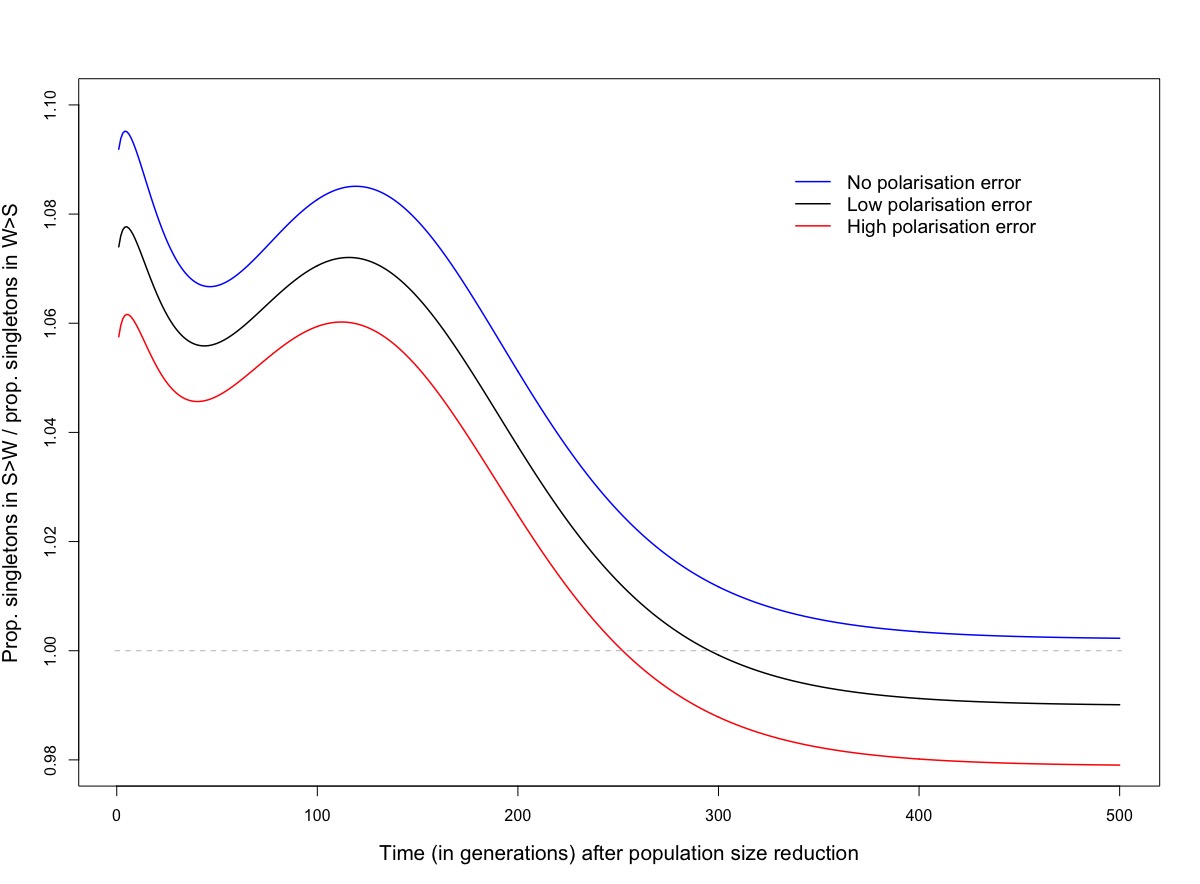** |
| --- |

# References

Glémin S, Arndt PF, Messer PW, Petrov D, Galtier N, Duret L. 2015. Quantification of GC-biased gene conversion in the human genome. Genome Res. 25:1215–1228.

Langley SA, Karpen GH, Langley CH. 2014. Nucleosomes Shape DNA Polymorphism and Divergence. PLoS Genet. 10:e1004457.

Messer PW, Petrov DA. 2013. Frequent adaptation and the McDonald-Kreitman test. Proc. Natl. Acad. Sci. U. S. A. 110:8615–8620.

Schrider DR, Houle D, Lynch M, Hahn MW. 2013. Rates and genomic consequences of spontaneous mutational events in Drosophila melanogaster. Genetics 194:937–954.

Sousa VC, Carneiro M, Ferrand N, Hey J. 2013. Identifying loci under selection against gene flow in isolation-with-migration models. Genetics 194:211–233.

Zeng K, Charlesworth B. 2009. Estimating selection intensity on synonymous codon usage in a nonequilibrium population. Genetics 183:651–662.

Zeng K, Charlesworth B. 2010. The effects of demography and linkage on the estimation of selection and mutation parameters. Genetics 186:1411–1424.
