# Supplementary material for: Variation in the Intensity of Selection on Codon Bias over Time Causes Contrasting Patterns of Base Composition Evolution in Drosophila
Source: Genome Biol Evol. 2017 Jan 12;9(1):102–23. doi: 10.1093/gbe/evw291 (PMC5381600; doi:10.1093/gbe/evw291)
Supplement: Supplementary Data [file evw291_Supp.zip › reply.R2.pdf]

Reviewers' Comments:

Referee: 2

Comments to the Author
The authors have satisfactorily addressed my main recommendation regarding the action of biased gene conversion. The results of the new analyses, as it happens, are more interesting than I had anticipated.

Referee: 1

Comments to the Author
The revised manuscript by Jackson et al has addressed most of the issues that were brought forth in the initial reviews and I think this work should be well worth publication in GBE with some important modifications/clarifications.

1) Evidence for AT-selection in the Dmel lineage. The discussion is important as it brings to light some caveats for inferring selection from SFS more generally. The revision includes some useful analysis and discussion of the previous findings in Poh et al (general fitness advantages for S->W mutations in dmel). p40, Supplementary Figure S14 is an important result: "we used parameter values realistic for D. melanogaster to show that, with demography and polarisation error, it is possible for the proportion of singletons in the SFS for SW changes to be lower than that for WS changes in the presence of weak selection favouring S." The figure should show "actual" ratios as well as "inferred" to make it clear that BOTH the demographic scenario and polarization error under parsimony contribute to the bias. Also the separate contributions should be stated more explicitly in the text. The multiple changes in sign of slope in S14 seems peculiar and should be discussed (explained). Finally, would SFS analysis (rather than comparison of singletons only) be more robust to the effects shown in S14?.

Reply: As suggested, the new Figure S14 has an additional curve in blue showing the effects of demography alone (i.e., the “actual” ratios). This should help the reader to appreciate the relative contributions of the two factors.

We have also added the following passages to the legend:

“It can be seen that, after the population size reduction, the proportion of singletons in the SFS for $S \to W$ mutations becomes very close to that in the SFS for $W \to S$ mutations in the absence of polarisation error (blue curve), as a result of the reduction in selective pressure. This, when combined with a higher probability of incorrectly identifying the ancestral states for $W \to S$ mutations, can lead to the proportion of singletons in the SFS for $S \to W$ changes being lower than that for $W \to S$ changes, even though there has been weak selection favouring S (see the black and red curves for low and high rates of polarisation errors, respectively). These results highlight the importance of taking into account the effects of demography when SFS-based summary statistics are used. The fluctuations that appeared shortly after the reduction in population size are

due to complex interactions involving non-equilibrium base composition, mutational bias, and relaxed selection, as noted previously by Zeng and Charlesworth (2009)."

We have added "(see the legend to Figure S14 for further discussion of this issue)" to p. 42 of the main text.

We have decided to present the above in the supplement because we feel that the Discussion is already rather long and complex. Adding such a section would harm the flow and reduce readability.

Finally, we believe that all SFS-based analyses should be carried out with caution in the presence of recent demographic changes. However, which statistic/approach is more robust would probably be case-specific, and it is beyond the scope of this study to examine this issue in detail.

2) Ancestral inference. Some aspects have been clarified but some information may be missing. "Reference" sequences are chosen from each species in order to infer ancestral sequences at the Dmel-Dsim ancestral node. These states are assumed to also reflect ancestral states at polymorphc sites. The same inferences (at the Dmel-Dsim ancestor) are used to infer "substitutions" on the lineage. How are substitutions counted: e.g. comparison to the reference sequence, comparisons to the full within-species sample (excluding reference seq)?

Reply: We have added the following to the Materials and Methods section (p. 17):

"We did this along each of the *D. melanogaster* and *D. simulans* lineages by (probabilistically) comparing the reconstructed ancestral states at the *ms* node with the reference genomes. This is reasonable because the branch length is much higher than the level of within-species polymorphism (see Results)."

3) The analyses at the beginning of the discussion (p35-37) take a similar approach to reach very similar findings to those in Akashi et al. 2007 and this should be noted (the work is cited elsewhere in the paper for other aspects). E.g, the main result: "negative relationship between Rn and GC content is consistent with a genome-wide reduction in the intensity of selection in both species" (also stated on p26,27) is shown in Fig 1 and is discussed in the previous work.

Reply: We have added citations to Akashi et al. (2007) to p. 36 and p. 38 in the Discussion.

4) The notation for mutation classes is given as SW, WS and "neutral" for SS/WW (P17 and elsewhere). The "neutral" designation seems presumptuous and is confusing in the SI analyses as all three classes are "neutral" with respect to fitness under BGC.

Reply: The labelling of variants as $S \rightarrow W$, $W \rightarrow S$ and putatively neutral (p. 17) stems from the fact that a main focus of the study is to estimate the strength of selection on codon usage bias. Using this notation, in the first half of the Results section, the data from the putatively neutral sites in short introns (the SI sites) are presented alongside data from 4-fold degenerate sites for comparison (Figures 1 – 4 and Table 2). For the sake of consistency, we have adopted the same system when the more detailed analyses of the SI sites are shown (Figure 5). To help avoid any confusion, we have added the following sentence to that section of the Results (p. 32):

"In contrast to selection on CUB at 4-fold sites, all alleles have equal fitness under the gBGC model, and the selection-like pattern is created by the preferential transmission of the S allele in SW heterozygotes to the next generation (Duret and Galtier 2009). The $S \rightarrow S$ and $W \rightarrow W$ mutations are "neutral" in the sense that they are should be unaffected by gBGC."

A simpler version has also been added to the Figure 5's legend to remind the reader:

"AT → AT or GC → GC mutations were labelled as neutral to signify that they should be unaffected by gBGC."
